# Identification and validation of genetic variants predictive of gait in standardbred horses

**DOI:** 10.1371/journal.pgen.1008146

**Published:** 2019-05-28

**Authors:** Annette M. McCoy, Samantha K. Beeson, Carl-Johan Rubin, Leif Andersson, Paul Caputo, Sigrid Lykkjen, Alison Moore, Richard J. Piercy, James R. Mickelson, Molly E. McCue

**Affiliations:** 1 Department of Veterinary Clinical Medicine, University of Illinois, Urbana, Illinois, United States of America; 2 Veterinary Population Medicine Department, University of Minnesota, St. Paul, Minnesota, United States of America; 3 Department of Medical Biochemistry and Microbiology, Uppsala University, Uppsala, Sweden; 4 Department of Animal Breeding and Genetics, Swedish University of Agricultural Sciences, Uppsala, Sweden; 5 Department of Veterinary Integrative Biosciences, Texas A&M University, College Station, Texas, United States of America; 6 Paul Caputo, DVM, Pompano Beach, Florida, United States of America; 7 Faculty of Veterinary Medicine, Norwegian University of Life Sciences, Oslo, Norway; 8 Moore Equine Services, Cambridge, Ontario, Canada; 9 Department of Clinical Sciences and Services, Royal Veterinary College, London, United Kingdom; 10 Veterinary and Biomedical Sciences Department, University of Minnesota, St. Paul, Minnesota, United States of America; The University of North Carolina at Chapel Hill, UNITED STATES

## Abstract

Several horse breeds have been specifically selected for the ability to exhibit alternative patterns of locomotion, or gaits. A premature stop codon in the gene *DMRT3* is permissive for “gaitedness” across breeds. However, this mutation is nearly fixed in both American Standardbred trotters and pacers, which perform a diagonal and lateral gait, respectively, during harness racing. This suggests that modifying alleles must influence the preferred gait at racing speeds in these populations. A genome-wide association analysis for the ability to pace was performed in 542 Standardbred horses (n = 176 pacers, n = 366 trotters) with genotype data imputed to ~74,000 single nucleotide polymorphisms (SNPs). Nineteen SNPs on nine chromosomes (ECA1, 2, 6, 9, 17, 19, 23, 25, 31) reached genome-wide significance (p < 1.44 x 10^−6^). Variant discovery in regions of interest was carried out via whole-genome sequencing. A set of 303 variants from 22 chromosomes with putative modifying effects on gait was genotyped in 659 Standardbreds (n = 231 pacers, n = 428 trotters) using a high-throughput assay. Random forest classification analysis resulted in an out-of-box error rate of 0.61%. A conditional inference tree algorithm containing seven SNPs predicted status as a pacer or trotter with 99.1% accuracy and subsequently performed with 99.4% accuracy in an independently sampled population of 166 Standardbreds (n = 83 pacers, n = 83 trotters). This highly accurate algorithm could be used by owners/trainers to identify Standardbred horses with the potential to race as pacers or as trotters, according to the genotype identified, prior to initiating training and would enable fine-tuning of breeding programs with designed matings. Additional work is needed to determine both the algorithm’s utility in other gaited breeds and whether any of the predictive SNPs play a physiologically functional role in the tendency to pace or tag true functional alleles.

## Author summary

Certain horse breeds have been developed over generations specifically for the ability to perform alternative patterns of movement, or gaits. Current understanding of the genetic basis for these gaits is limited to one known mutation apparently necessary, but not sufficient, for explaining variability in “gaitedness.” The Standardbred breed includes two distinct groups, trotters, which exhibit a two-beat gait in which the opposite forelimb and hind limb move together, and pacers, which exhibit an alternative two-beat gait where the legs on the same side of the body move together. Our long-term objective is to identify variants underlying the ability of certain Standardbreds to pace. In this study, we were able to identify several regions of the genome highly associated with pacing and, within these regions, a number of specific highly associated variants. Although the biological function of these variants has yet to be determined, we developed a model based on seven variants that was > 99% accurate in predicting whether an individual was a pacer or a trotter in two independent populations. This predictive model can be used by horse owners to make breeding and training decisions related to this economically important trait, and by scientists interested in understanding the biology of coordinated gait development.

## Introduction

Gait refers to a pattern of limb movement during locomotion, and can be defined by patterns of footfall and symmetry or asymmetry, among other factors. In quadrupeds, a limited number of gaits are conserved among species, including the walk (4-beat, symmetric), trot (two-beat, symmetrical, diagonal), and gallop (4-beat, asymmetric). Deviations from normal gait patterns are suggestive of underlying musculoskeletal or neurologic abnormalities. However, certain breeds of horses, including the Standardbred, Icelandic horse, Tennessee Walking Horse, and Paso Fino, have been specifically selected over generations of breeding for their ability to perform alternative patterns of locomotion. These alternative gaits are typically of intermediate speed and replace the trot. For example, the pace is a 2-beat lateral symmetrical gait, and the tolt is a 4-beat lateral symmetrical gait (**S1 Fig**).[1] There are strong signatures of selection evident when comparing gaited and non-gaited breeds[2], and the trait is highly heritable; for example, heritabilities of the pace and tölt in the Icelandic horse have been estimated to range between 0.53 and 0.73.[3] However, until recently, the specific genetic determinants underlying these alternative gaits were completely unknown.

In 2012, a genome-wide association study (GWAS) in four-gaited (walk, trot, tölt, and gallop) and five-gaited (walk, trot, tölt, gallop, and pace) Icelandic horses revealed a strongly associated SNP on equine (ECA) chromosome 23.[1] Deep (30x coverage) whole-genome sequencing of one four-gaited and one five-gaited individual revealed a premature stop codon in the last exon of *DMRT3* (an isoform of the doublesex and mab-3 related transcription factor). Subsequent genotyping of additional Icelandic horses revealed that nearly all five-gaited individuals were homozygous for the mutation, compared to only a third of the four-gaited horses. Of even greater interest, when horses of other breeds were genotyped for the mutation, it was found to be nearly fixed in gaited breeds (e.g. Paso Fino, Peruvian Paso, Tennessee Walking Horse, Standardbred.), but absent in non-gaited breeds (e.g. Arabian, Thoroughbred).[1] The functional importance of *DMRT3* was confirmed in a mouse model, where mice null for *DMRT3* exhibited an abnormal gait characterized by an increased stride, prolonged, stance and swing phases of both the thoracic and pelvic limbs, and near absence of coordinated pelvic limb movements. Further, *DMRT3* expression was localized to the spinal cord both pre- and postnatally, and null mice had fewer commissural interneurons, suggesting that this gene is important for the development of normal locomotor coordination.[1]

Although the *DMRT3* mutation appears to be necessary for “gaitedness” in horses it is not sufficient to explain the variation of this trait, as demonstrated by the fact that it is nearly fixed in Standardbreds, although not all individuals exhibit that breed’s alternative gait, pacing.[1] It is noteworthy that approximately 20% of the offspring of Standardbred trotter stallions go on to race as pacers.[4] It is unknown whether this is due to genetic predisposition, training, or a combination of the two, but it is likely that modifying genetic factors segregate in the Standardbred population and determine an individual’s ability to pace. The purpose of this study was to identify putative modifying alleles associated with gait in a large cohort of Standardbred pacers and trotters using a combination of GWAS and variant discovery via whole-genome sequencing.

## Results

### GWAS analysis

**Fig 1:**
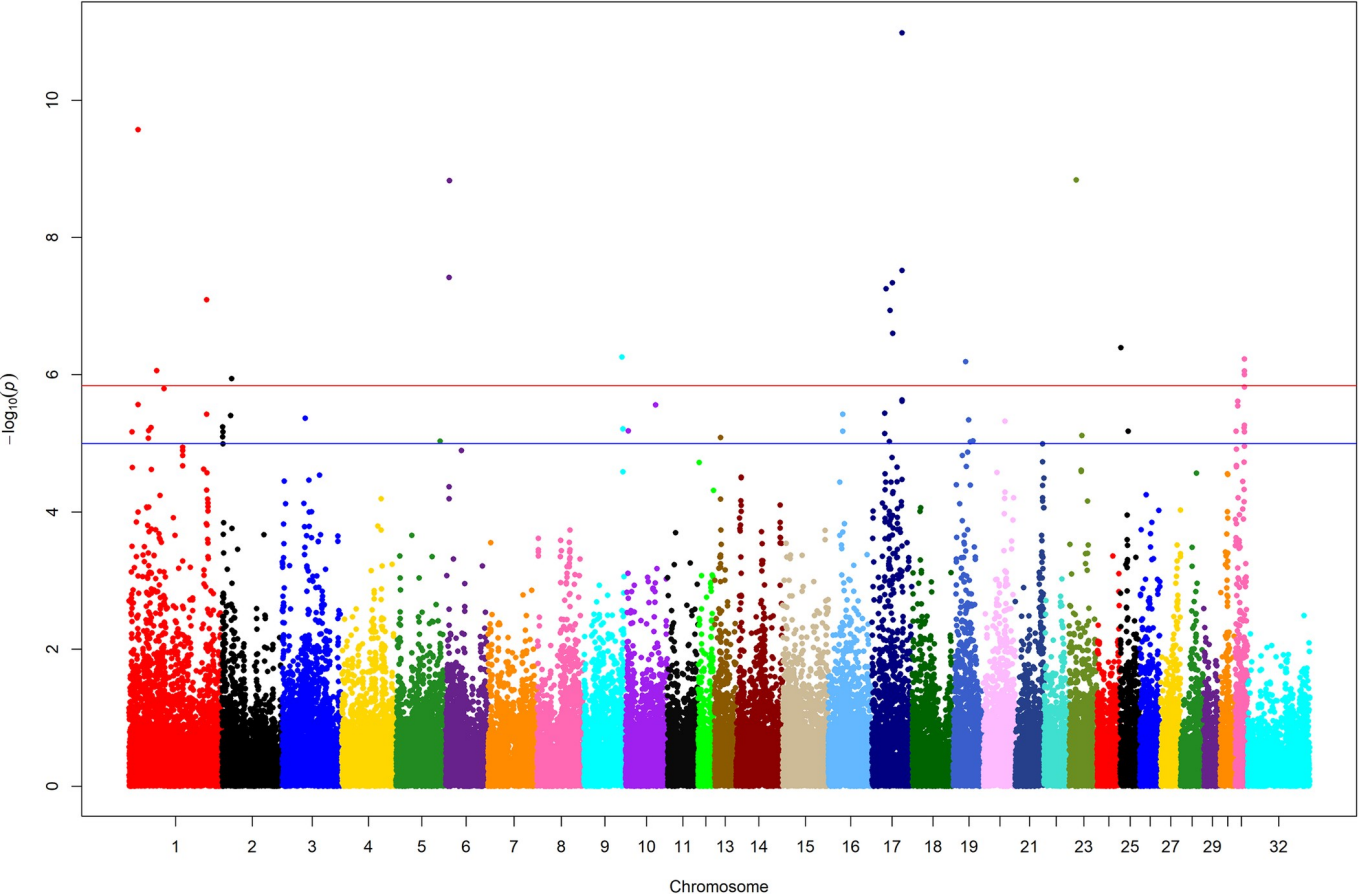
Manhattan plot of results from mixed model analysis using GEMMA. The 31 autosomal and X chromosome (32) are represented in different colors along the x-axis and the–log(p-value) is on the y-axis. Each colored dot represents a SNP. Genome-wide significant hits are on ECA1, 2, 6, 9, 17, 23, 25, and 31. See **Table 1** for specific SNPs and p-values. The red horizontal line represents the level of genome-wide significance (p < 1.44 x 10^−6-^); the blue line represents a cutoff for moderate association (p < 1 x 10^−5^).

Horses included in the GWAS cohort (n = 542) were genotyped on either the first generation (Equine SNP50; n = 306) or second generation (Equine SNP70; n = 236) Illumina equine beadchip. After genotype imputation, 73,691 markers were available for analysis; after pruning, 62,901 SNPs were included in the mixed model association analysis. After correction for relatedness and population structure, mixed model association analysis in GEMMA[5] revealed 19 SNPs on nine chromosomes that reached genome-wide significance (p < 1.44 x 10^−6^, as determined by the likelihood ratio test) (**Fig 1, Table 1**). Seven SNPs were located on equine (ECA) chromosome 17, three on ECA1, two on ECA6, two on ECA31, and one each on ECA2, 9, 19, 23, and 25. The ECA17 SNPs were from two distinct regions, 28.4–41.9Mb (n = 4) and 60.50–60.55Mb (n = 3) (**Table 1**). Only eight of the genome-wide significant SNPs were found within protein coding genes; all were intronic (**Table 1**). An additional 37 SNPs on 14 chromosomes reached significance considered to be moderately associated with gait (p < 1 x 10^−5^) [6] (**S1 Table**).

**Table 1:** Genome-wide significant (p < 1.44 x 10^−6^) SNPs from GEMMA mixed model analysis in 542 Standardbred pacers and trotters (sex and origin covariates). After pruning, analysis included 62,901 SNPs. Uncorrected p-values are presented for the Likelihood ratio test (LRT). CHR = chromosome; BP = base pair. Original gene annotations are from Ensembl (Equ Cab 2; GCA_000002305.1). Genes that were predicted, but unnamed, in Ensembl were identified via BLAST (https://blast.ncbi.nlm.nih.gov/Blast.cgi) when possible. SNPs were subsequently re-mapped to Equ Cab 3.0 using BLAST (NCBI).

CHR	Region	EquCab2	EquCab3	P_LRT	Location	Genes in Region
1	18.1Mb	18091577	18209783	9.1E-10	intergenic	*CASP7*, *NRAP*
1	55.3Mb	55259288	55705873	1.4E-06	intron 3	*CTNNA3*
1	155.2Mb	155226154	156822538	2.0E-07	intergenic	*OR4Q3*, *OR11H1*-like, *OR11G2*-like
2	19.7Mb	19755735	19803462	1.4E-06	intron 2	*SFA3*
6	7.0–7.4Mb	7000504	6774154	3.3E-08	intergenic	no named or predicted genes
		7372690	7146326	9.6E-10	intergenic	
9	76.3Mb	76324169	78430548	8.2E-07	intergenic	no named or predicted genes
17	28.4–41.9 Mb	28460851	28363078	2.7E-07	intron 26	*VWA8*
		36291973	36195182	2.8E-07	intergenic	*NAA16*, *MTRF1*, *KBTBD7*, *WBP4*, *ELF1*, *SUGT1*, *CNMD*, *PCDH8*, *OLFM4*, *PCGF5*, *RGCC*, *KBTBD6*, *PCDH17*, *RPL7A*, *DIAPH3*, *TDRD3*, *PCDH20*, *TDGF1*, *ATP6V1G3*, *PCDH9*, *ENSECAG00000001575*, *ENSECAG00000016249*, *ENSECAG00000016703*, *ENSECAG00000001672*, *ENSECAG00000001728*, *ENSECAG00000001792*
		40999944	40904434	1.3E-07	intergenic	
		41905502	41810361	2.0E-07	intergenic	
17	60.50–60.55 Mb	60503138	60393995	5.2E-08	intergenic	*ENSECAG00000002865*
		60523882	60414731	5.2E-08	intergenic	
		60554458	60446633	3.9E-11	intergenic	
19	24.64Mb	24644810	27049559	4.6E-07	intron 5	*DNAJB11*
23	14.6Mb	14650375	14017592	1.9E-09	intron 1	*PRUNE2*
25	2Mb	2021044	2054596	2.2E-07	intron 8	*PAX5*
31	18.19–18.20 Mb	18194086	18237471	9.6E-07	intron 2	*SASH1*
		18200337	18243722	7.1E-07	intron 1	

### Whole-genome sequencing

We have previously reported the results of whole-genome sequencing in this cohort.[7] Briefly, 12 individuals (6 pacers, 6 trotters) were sequenced at an average coverage depth of 6.4x (range 4.7x-7.9x). Six individuals (3 pacers, 3 trotters) were sequenced at an average coverage depth of 12.2x (range 10x-13.1x). After filtering, 14,588,812 variants were called, of which 13,157,608 were SNPs, 671,144 were insertions, and 760,060 were deletions. Of these variants, 99.1% were predicted to have no functional effect, 0.5% (85,916) were predicted to have minor functional effect, 0.4% (57,122) were predicted to have moderate functional effect, and 0.07% (9,662) were predicted to have major functional effect.

### Pooled whole-genome sequencing

Two pools of genomic DNA (20 pacers, 20 trotters) were sequenced at a target depth of 30x. A total of eighty-nine 50kb regions met one of the filtering criteria of either high differentiation between pacers and trotters (F_ST_ ≥ 0.35) or a combination of low pool heterozygosity (Hp < 0.1) in one of the groups and high differentiation (F_ST_ ≥ 0.30). Some of these regions were contiguous or overlapping, creating larger regions of interest ranging in size from 75-300kb (**S2 Table**). A total of 1,885 SNPs were called across all regions of interest. Of these, 1,273 were annotated by Ensembl (Equ Cab 2; GCA_000002305.1) as intergenic, 184 were located upstream of a gene, 138 were located downstream of a gene, 270 were located within an intron, and 20 were located within an exon (**S3 Table**).

### Sequenom genotyping in the discovery cohort

Approximately 62,000 SNPs were evaluated from regions on 13 chromosomes identified as being of interest based on our GWA analysis (regions from 9 chromosomes that contained genome-wide significant SNPs, and regions from an additional 4 chromosomes that contained SNPs approaching genome-wide significance). An additional 1,885 SNPs were identified within regions of interest on 19 chromosomes from the pooled sequencing data. Three hundred three SNPs were included in the final Sequenom assay, including 190 SNPs from the whole-genome sequencing regions of interest (based on the GWA analysis) and 113 SNPs from the pooled sequencing regions of interest (**S4 Table**). Additionally, 98 ancestry informative markers (AIMs) were included on the assay to help control for population structure during downstream analysis (see **Methods** and [7]).

Genotyping was performed in 720 individuals (n = 458 trotters; n = 262 pacers). After pruning, 245 SNPs were available for mixed model association analysis in GEMMA. With Bonferroni correction for multiple testing, statistical significance was set at p < 2 x 10^−4^; after correcting for relatedness and population structure, 177 SNPs met this criteria for statistical significance (**Table 2, S5 Table**). Pacers were more likely than trotters to carry the derived alleles (compared to the reference, a Thoroughbred) (**Table 2**). Nearly all of the trotters carried only a single copy of the alternate allele in each case, while it was more common for pacers to be homozygous for the alternate allele.

**Table 2:** Summary of top 40 SNPs from GEMMA mixed model analysis in 720 standardbred pacers and trotters genotyped on the custom sequenom assay, including frequency of the alternate allele in pacers and trotters at each SNP. After pruning, analysis included 245 SNPs. Uncorrected p-values are presented for the Likelihood ratio test (lrt). A summary of all statistically significant SNPs from this analysis, including results from all three frequentist tests performed, can be found in **S5 Table**. CHR = chromosome. SNPs were subsequently re-mapped to Equ Cab 3.0 using BLAST (NCBI).

Rank	CHR		EquCab 2	EquCab3	p_lrt	Pacer Freq	Trotter Freq
**1**	30	14067984		14903592	1.4E-38	0.93	0.22
**2**	30	14107178		14942789	2.2E-38	0.08	0.79
**3**	17	28540291		28442518	2.3E-38	0.70	0.07
**4**	17	28361747		28263936	4.0E-37	0.58	0.02
**5**	23	14640812		14008017	1.0E-36	0.20	0.92
**6**	23	14648590		14015807	1.1E-36	0.80	0.09
**7**	23	14649864		14017081	1.2E-36	0.21	0.92
**8**	30	14947553		15783205	3.7E-33	0.07	0.74
**9**	1	35729338		35978275	9.7E-33	0.70	0.08
**10**	1	35731849		35980730	1.1E-32	0.70	0.08
**11**	1	35731283		35980220	1.3E-32	0.70	0.08
**12**	1	35726345		35975239	1.9E-32	0.71	0.09
**13**	1	35720250		35969132	1.9E-32	0.70	0.08
**14**	30	15055793		15891465	2.3E-32	0.93	0.25
**15**	30	15068782		15904456	2.5E-32	0.93	0.26
**16**	17	28347510		28249699	8.1E-31	0.56	0.02
**17**	30	14936139		15771790	1.1E-29	0.91	0.26
**18**	1	17945265		18064350	2.7E-29	0.93	0.24
**19**	1	35721326		35970208	5.3E-29	0.70	0.09
**20**	17	28458432		28360659	1.9E-28	0.71	0.15
**21**	30	15124747		15960395	1.4E-27	0.86	0.26
**22**	3	3051017		3178607	1.2E-26	0.76	0.15
**23**	17	28658966		28561034	2.5E-26	0.56	0.07
**24**	23	20652865		20036611	4.0E-26	0.82	0.14
**25**	23	20662320		20046132	6.6E-26	0.82	0.14
**26**	20	27691110		28595503	2.1E-24	0.63	0.07
**27**	1	48896092		49259272	6.6E-24	0.54	0.08
**28**	25	11811829		11842351	7.2E-22	0.77	0.16
**29**	1	38573734		38823333	9.2E-22	0.23	0.83
**30**	17	29271555		29173660	1.3E-21	0.55	0.05
**31**	25	11783623		11832785	1.6E-21	0.77	0.16
**32**	30	14059751		14895359	3.6E-21	0.15	0.69
**33**	17	28658850		28560918	4.5E-21	0.54	0.07
**34**	25	11800074		11849233	7.5E-21	0.76	0.16
**35**	1	38591441		38841042	2.6E-20	0.77	0.21
**36**	1	38592542		38842143	4.2E-19	0.77	0.22
**37**	23	14645077		14012301	1.8E-18	0.16	0.78
**38**	25	15839070		16233979	3.0E-18	0.83	0.21
**39**	1	38306816		38556327	7.0E-18	0.71	0.13
**40**	3	2494992		2627642	9.0E-18	0.70	0.18

### Random forest classification analysis

Random forest classification analysis of genotyping data from the Sequenom assay in 659 Standardbreds with racing records (n = 428 trotters; n = 231 pacers) yielded an out-of-box (OOB) error rate of 0.61%, with a total of four misclassified individuals (three trotters misclassified as pacers, and one pacer misclassified as a trotter). Interestingly, one of the trotters who was predicted to be a pacer was in fact out of a line of pacing Standardbreds; it is not known whether this horse was trained as a pacer at any point before starting its racing career. There were 21 SNPs with a mean reduction of node impurity score (GINI index) > 5 (**Table 3**). When the random forest analysis was repeated, the relative importance of these SNPs varied only slightly over multiple iterations. The most important SNPs for classification as a pacer or trotter according to this analysis were located on ECA1, 17, 23, and 30. Ten-fold cross-validation of this data using linear discriminate analysis resulted in a misclassification error of 0.0106.

**Table 3:** Results of random forest analysis of 303 SNPs in 659 standardbreds with race records. Importance scores are reported as a GINI index, reflecting reduction in node impurity. The higher the GINI index, the more misclassification is introduced by random permutation of the variable. CHR = chromosome. SNPs were subsequently re-mapped to Equ Cab 3.0 using BLAST (NCBI).

CHR	EquCab2	EquCab3	Allele	MeanDecreaseGini
**17**	28347510	28249699	G	15.05209
**30**	14947553	15783205	G	13.06787
**30**	14067984	14903592	G	13.03264
**1**	17945265	18064350	C	12.19836
**17**	28361747	28263936	G	11.95995
**30**	14107178	14942789	A	11.37835
**23**	14648590	14015807	A	10.82176
**23**	14649864	14017081	C	10.18182
**30**	15068782	15904456	G	9.61353
**23**	14640812	14008017	G	9.09522
**1**	35731283	35980220	T	7.759352
**23**	20652865	20036611	A	7.559141
**17**	28540291	28442518	A	7.443625
**30**	15055793	15891465	C	6.630535
**1**	35731849	35980730	G	6.608307
**1**	35729338	35978275	T	6.585548
**30**	14936139	15771790	A	6.334448
**23**	20662320	20046132	A	5.789609
**1**	35720250	35969132	C	5.701313
**1**	35721326	35970208	C	5.494415
**1**	35726345	35975239	T	5.341378

**Fig 2:**
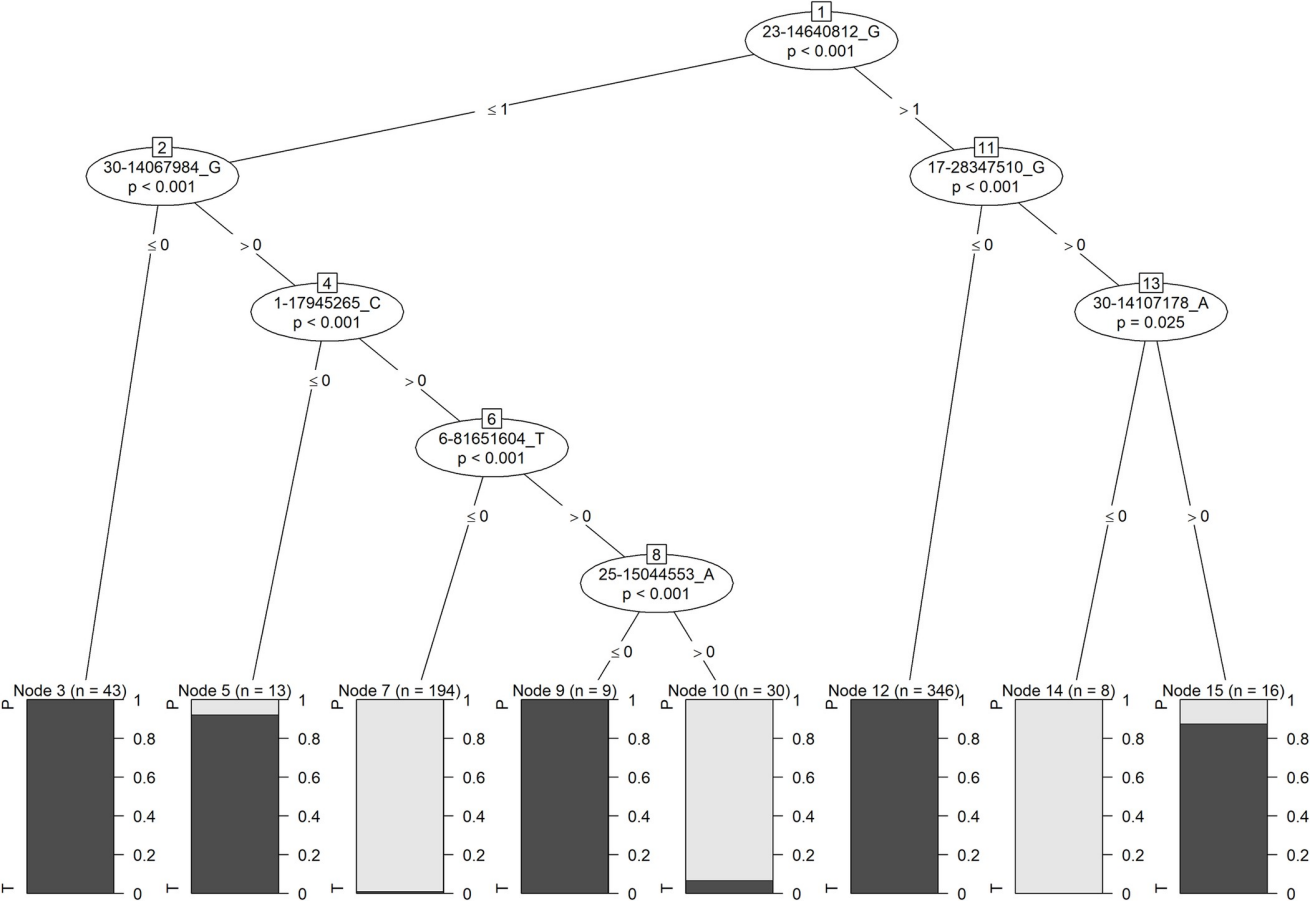
Conditional inference tree based on genotyping results in 659 standardbreds with race records. Grey nodes are pacers; black nodes are trotters. Nodes 5, 7, 10, and 15 contain misclassified individuals (total n = 6).

A conditional inference tree was constructed to determine the hierarchical organization of the most informative SNPs identified by random forest analysis. A tree composed of seven SNPs predicted status as a pacer or trotter among the 659 genotyped individuals with 99.1% accuracy, with only six horses misclassified (three pacers and three trotters). Again, one of these misclassified trotters came from a line of pacers. Considering pacing as the outcome of interest, this prediction model demonstrated a sensitivity of 98.7% (95% CI 96.25% - 99.73%) and a specificity of 99.3% (95% CI 97.97% - 99.86%). The seven SNPs were located on six chromosomes (ECA1, 6, 17, 23, 15, and 30) (**Fig 2**). For four SNPs, the alternate allele was more common in pacers, and in three SNPs the alternate allele was more common in trotters. In either case, the group with the lower allele frequency included very few homozygotes (**Table 4**).

**Table 4:** Alternate allele frequencies for each of the 7 SNPs in the conditional inference tree. CHR = chromosome. SNPs were subsequently re-mapped to Equ Cab 3.0 using BLAST (NCBI).

CHR	EquCab2	EquCab3	Allele	Pacer Freq	Trotter Freq
**23**	14640812	14008017	G	0.21	0.92
**17**	28347510	28249699	G	0.57	0.02
**30**	14107178	14942789	A	0.08	0.79
**30**	14067984	14903592	G	0.93	0.23
**1**	17945265	18064350	C	0.93	0.23
**6**	81651604	82815419	T	0.07	0.52
**25**	15044553	15446284	A	0.78	0.18

### Performance of the prediction algorithm in the validation cohort

One hundred sixty-six horses (n = 83 trotters, n = 83 pacers) were genotyped on the same custom Sequenom assay as the discovery cohort. The genotypes for the seven SNPs included in the conditional inference tree were extracted. Two individuals had missing genotypes at one or more of the SNPs and could not be classified. Of the remaining 164 horses, 163 were correctly classified as pacers or trotters, resulting in an overall accuracy of 99.4%. The single misclassified horse was a pacer.

## Discussion

The horse is unique among quadrupeds in that certain breeds have been strongly selected for the ability to exhibit alternative patterns of locomotion as a physiologic adaptation. Beyond giving insight into an economically important trait, improved understanding of the pathways that underlie alternative gaits in the horse may also provide insight into pathways that are dysregulated with disease in other species, as well as basic insight into the underlying neurobiology of locomotion. GWA analysis in our population identified 19 SNP markers that were associated with gait at a level of genome-wide significance. These SNPs defined regions of interest on nine chromosomes that contained more than two dozen named genes. However, a challenge arises in identifying biologically compelling candidate genes for gait because there is still much that is not known about the development of normal limb coordination. It is likely that many genes that play a role in the development of alternative gaits have not previously been associated with any aspect of neurobiology. This is aptly illustrated by *DMRT3* which had initially been described as primarily playing a role in gonadal development and sexual differentiation.[8] The *DMRT3* nonsense mutation originally reported by Andersson et al. in 2012 [1] has now been reported to occur in 68 out of 141 breeds tested from around the world, and at high frequency (>50%) in all “gaited” breeds.[9] This example demonstrates that a strongly associated mutation cannot be ruled out as having a functional role in the development of alternative gaits simply because it falls within a gene that does not have a described role in neural development or locomotion.

As SNPs are chosen for inclusion in genotyping panels based on their distribution and frequency, rather than on predicted effect, it is unlikely that any of the markers in the GWA analysis play a functional role in gait. Rather, it is more likely that they are “tagging” truly functional variants with which they are in linkage disequilibrium (LD).[10, 11] Standardbreds have been reported to have the greatest long-range LD (> 1,200kb) among horse breeds; thus, it is not unreasonable to expect that a significant SNP in a GWA might be “tagging” a functional sequence variant up to 1Mb distant (or further). Given this, we chose whole-genome sequencing as the most efficient way to catalogue variants within 1Mb of the regions defined by our genome-wide significant SNPs. This approach also allowed variant discovery in a larger cohort of individuals (9 trotters and 9 pacers) than would have been feasible using a traditional candidate gene approach, giving us a better picture of the alleles present in our population, as well as the segregation of these alleles with gait status. Pooled whole-genome sequencing offered a complementary approach to identifying regions and variants of interest based on population parameters of differentiation (F_ST_) and heterozygosity (Hp). Pooled whole-genome sequencing has previously been used to identify genomic regions under selection, and identify candidate variants within those regions, in a number of plant and animal species.[12–14]

Of the tens of thousands of SNPs discovered within regions of interest via whole-genome sequencing, only a small fraction could be selected for follow-up. Thus, it is likely that we have not identified any truly functional alleles despite our prioritization process. Indeed, of the top 40 variants from GEMMA analysis of Sequenom genotyping in 720 individuals, only two resulted in amino acid changes. However, given the strength of association of our selected variants with gait in this large population, it is highly likely that one or more of these genotyped variants are “tagging” specific functional alleles, and additional investigation of nearby variants is warranted. It must be noted that given the strong population structure of our cohorts, inherent to the history of this breed which has undergone strong selection for our trait of interest over many generations, it is also possible that one or more of these variants are reflecting some other aspect of population structure unrelated to gait. Future work to address the issue of functionality will include tissue expression profiles of the cerebellum and specific regions of the proximal cervical spinal cord that contain neuronal tracts known to play a role in coordinated locomotion.

Random forest classification analysis was selected to help prioritize among the numerous statistically significant variants in our Sequenom assay. In a random forest approach to a binary trait, the predicted probability of an individual expressing or not expressing a trait (in this case, being able to pace) is based on the aggregation of a number of decision trees.[15, 16] Within these decision trees, each node is an attribute–in this case, the genotype at a given SNP. The importance of each SNP is determined by quantifying the increase of misclassified individuals when the genotype at that SNP is randomly permuted.[15] This approach requires no prior knowledge of gene function and can accommodate multiple variants within the same gene. Random forest analysis has previously been successfully used to identify SNPs associated with feed intake in dairy cattle[17] as well as pathway-phenotype associations in human bladder cancer.[16] In our population, random forest analysis revealed that just a few SNPs were of large importance in correctly classifying individuals, while a large number of SNPs were of minor to minimal importance. This suggested that an accurate prediction algorithm might be constructed, despite not knowing the functional importance of the individual SNPs. In fact, we were able to develop a prediction algorithm consisting of only seven SNPs that was > 99% accurate in two independently sampled cohorts of Standardbreds. The small number of misclassified individuals may have had an environmental component (i.e. training) that explains why they were competing at a different gait than expected, or it may simply reflect room for refinement of the algorithm by the identification of truly functional SNPs. This is the first time that a prediction algorithm for gait has been reported and it could be used by owners/breeders/trainers for both marker-assisted selection and making training decisions by identifying young horses that have the genetic background to race successfully at the pace. This model will need to be tested in other breeds to determine if its predictive value is specific to Standardbreds, to breeds that pace (e.g. Icelandic Horses), or if it is universally applicable across gaited breeds.

## Materials and methods

### Study population

#### Ethics statement

This study was conducted under the approval of the University of Illinois (protocol #15031) and University of Minnesota (protocol #1111B07193) Institutional Animal Care and Use Committees.

#### GWAS cohort

The cohort for the genome-wide association study consisted of 542 Standardbred trotters (n = 366) and pacers (n = 176). All of the pacers and 153 trotters were from North America, while the remaining trotters were from Europe (Sweden, n = 66; Norway, n = 147). Horses were classified as pacers or trotters based on race records; if a horse never raced, their gait was assigned based on the race records of the sire and/or dam. Horses that raced as both pacers and trotters were classified as pacers. The North American and European trotters were related to each other. The pacers were genetically distinct from the trotters, with minimal admixture between groups (**S2 Fig**).

#### Sequenom assay discovery cohort

Initially, 720 Standardbreds from North America and Europe were genotyped on the custom Sequenom assay (see **Materials and Methods** - **Sequenom Assay**), including the entire GWAS cohort. Horses were classified as pacers or trotters based on race records; if a horse raced at both gaits, it was classified with the gait at which it raced most successfully. Horses without a race record were excluded from downstream analysis, resulting in a final cohort comprised of 659 Standardbred trotters (n = 428) and pacers (n = 231) from North America (n = 374) and Europe (n = 285).

#### Validation cohort

The validation cohort was comprised of 166 independently sampled Standardbred trotters (n = 83) and pacers (n = 83) from North America. Horses were classified as pacers or trotters based on race records. These horses were genotyped on the custom Sequenom assay as described above, and the genotypes at the SNPs included in the prediction algorithm were extracted for analysis. Approximately 10% of the horses in the discovery and validation cohorts had some degree of admixture (between pacer and trotter) found within a 4-generation pedigree.

### DNA isolation and whole-genome genotyping

DNA was isolated from whole blood samples using the Gentra Puregene Blood Kit (Qiagen, Valencia, CA) per manufacturer recommendations. Briefly, RBC lysis solution was added to samples at a 3:1 ratio, incubated, and centrifuged. After discarding the supernatant, Cell lysis solution was added to the white blood cell pellet and the cells were re-suspended, after which protein was precipitated and discarded. DNA was precipitated in isopropanol and subsequently washed in ethanol prior to final hydration. Quantity and purity of extracted DNA were assessed using spectrophotometric readings at 260 and 280nm (NanoDrop 1000, Thermo Scientific, Wilmington, DE).

Genome-wide genotyping of single nucleotide polymorphism (SNP) markers was performed by Neogen GeneSeek (Lincoln, NE) using an Illumina Custom Infinum SNP genotyping platform. Horses were genotyped at either 54,602 SNPs using the first generation Illumina Equine SNP50 chip (n = 306), or at 65,157 SNPs using the second generation Illumina Equine SNP70 chip (n = 236).

### Genotype imputation

The two genotyping platforms used in the GWAS cohort share 45,703 SNPs. As an alternative to losing information from tens of thousands of SNPs by pruning to this shared marker list prior to merging files, genotype imputation may be used. This technique statistically estimates genotypes from non-assayed SNPs based on a comparison of haplotype blocks between one population and a second, more densely genotyped reference population. An established pipeline for imputation of equine genotyping data [18] was used to impute the ~18,000 markers unique to the SNP70 chip in those horses genotyped on the SNP50 chip, and likewise to impute the ~9,000 markers unique to the SNP50 chip in those horses genotyped on the SNP70 chip. Imputed files were merged for subsequent analysis using the—merge command in PLINK.[19]

### Genome-wide association (GWA) analysis

A GWA analysis with gait as the phenotype of interest was performed after genotype imputation using GEMMA (Genome-Wide Mixed Model Analysis) software.[5] A centered relatedness matrix (-gk 2) was constructed using a LD-pruned set of approximately 6600 markers (100 SNP windows, sliding by 25 SNPs along the genome, pruned at r^2^ > 0.2; PLINK command—indep-pairwise 100 25 0.2).[20] All three possible frequentists tests were performed: Wald, likelihood ratio, and score (-fa 4). A covariate file including sex and origin (North America or Europe) was incorporated into the mixed model (-c) and SNPs were pruned according to GEMMA default parameters (MAF <1%, missingness <95%). Association plots were generated using the base graphics package in the R statistical computing environment.[21] Genome-wide significance was set at p < 1.44 x 10^−6^ based on the effective number of independent tests in our data.[22]

### Whole-genome sequencing

We have previously reported whole-genome sequencing in this cohort.[7] For the purposes of the previous study, horses were selected for sequencing based on haplotypes in regions of interest associated with osteochondrosis; however, they were also selected in a balanced manner for their gait phenotype, with 9 pacers and 9 trotters included. Briefly, genomic DNA (2–6μg) from the 18 horses was submitted to the University of Minnesota Biomedical Genomics Center (UMGC) for quality control, library preparation, and sequencing. Samples were subjected to library preparation including fragmentation, polishing, and adaptor ligation, and were prepared with an indexed barcode for a 100bp paired-end run on the Illumina HiSeq sequencer, per standard protocols. Targeted depth of coverage was 12x for six horses and 6x for twelve horses, with each group balanced for gait.

Data analysis, including quality control, alignment, and variant detection, was carried out following published best practices[23, 24] within the Galaxy framework hosted by the Minnesota Supercomputing Institute. Briefly, reads that passed quality control were mapped to the reference sequence (EquCab 2.0, Sept. 2007 [25]) using BWA for Illumina[26]. Ambiguously mapped reads, low quality reads, and PCR duplicates were removed, after which reads were realigned around indels. Base quality recalibration was performed to remove systematic bias. This process was completed for the reads from each of the eight lanes for every individual before merging the mapped and recalibrated “lane-level” BAM files into a single “sample-level” file. Removal of duplicates and realignment around indels was repeated on the merged file. The eighteen sample-level files were merged into three groups of six, evenly divided between pacers and trotters, for the purposes of variant calling using the UnifiedGenotyper utility of the Broad Institute’s Genome Analysis ToolKit (GATK)[27] with a threshold phred-scale score of 20.0. Variants were filtered using the following thresholds: Quality Depth (QD) < 2.0 (assesses variant quality score taking into account depth of coverage at that variant), Read Position Rank Sum < -20.0 (Mann-Whitney Rank Sum test on the distance of the variant from the end of each read covering it), Fisher Strand (FS) > 200.0 (phred-scaled p-value to detect strand bias). Filtered variant lists from the three groups were combined into a single variant calling file (VCF) for subsequent analysis. Predicted functional effect for each called variant was determined based on the current equine reference genome annotation using SnpEff.[28] Frequency of variants within cases and controls, and the significance of frequency differences, was calculated using SnpSift CaseControl.[29] Variants from particular chromosomal regions of interest were selected using SnpSift Intervals and converted into Excel format for further evaluation.

### Pooled whole-genome sequencing

Genomic DNA from 20 pacers and 20 trotters were combined into two pools, each comprising equimolar amounts of DNA from all individuals, for the purpose of whole-genome sequencing. These individuals were selected from the GWAS cohort and were chosen to be as unrelated as possible on the basis of coancestry coefficient generated from the whole-genome genotyping data (PLINK command—genome). Selected pacers had coancestry coefficients <0.06 (no more closely related than first cousins); selected trotters had coancestry coefficients <0.14 (one pair of half-siblings, the rest less closely related).

The two DNA pools were sequenced to 30X average depth of coverage using an Illumina HiSeq2500 sequencer at Uppsala University. The resulting paired reads were subjected to sequencing adaptor trimming and were subsequently aligned to the horse reference genome (EquCab2.1)[25] using the Burrows-Wheeler alignment algorithm as implemented in BWA for Illumina [26] (bwa sampe) using default alignment settings. Aligned reads were subjected to duplicate removal using the algorithm MarkDuplicates implemented in the Picard-tools software (https://broadinstitute.github.io/picard/). SNP and small insertion/deletion calling was performed using the UnifiedGenotyper algorithm of the Genome Analysis Toolkit (GATK) [27], and the resulting SNP calls were filtered using published best practice variant filtration settings.[23, 24] Numbers of sequence reads corresponding to the reference and variant alleles at filtered SNP sites were determined and were used to estimate allele frequencies for the pacer and trotter pools at each SNP locus. The allele counts and frequencies were then used to calculate the fixation index (F_ST_) for the contrast between the two pools and to calculate estimated pool heterozygosity (Hp) within each pool for 50% overlapping sliding windows of 50 kilobases along the genome as previously described.[12] Distributions of the genome-wide F_ST_ and Hp values were consulted to determine the genomic intervals displaying the most strongly differentiated loci between the pools and the most strongly fixed loci within each pool, respectively. From these distributions we used a strategy where windows fulfilling one of two criteria, (1) F_ST_ ≥ 0.35 or (2) F_ST_ ≥ 0.30 with the additional criteria of at least one pool showing Hp <0.1, were selected as regions of interest.

### Sequenom assay

A custom Sequenom genotyping assay was designed for high-throughput evaluation of prioritized variants. Variants were selected from top regions of interest identified in the GWA, as well as from regions from the pooled sequencing data with high differentiation between pacers and trotters (F_ST_ ≥ 0.35) or a combination of low pool heterozygosity (Hp < 0.1) in one of the groups and high differentiation (F_ST_ ≥ 0.30). SNPs discovered via whole-genome sequencing that passed quality control filters were prioritized according to the following parameters: 1) segregation with gait (preferentially with the alternate allele found in all or nearly all pacers and less than half of the trotters); 2) not intergenic; 3) non-synonymous, then synonymous changes; 4) if intronic, close to the exon-intron border (preferably < 100bp); 5) coding genes preferred over non-coding; and 6) if upstream/downstream, as close as possible to start/stop codon. Variants from pooled sequencing data were prioritized according to criteria 2–6.

When possible, at least one variant was selected from each coding gene within each region of interest. Among adjacent variants with equal magnitude of predicted functional effect, the one with the higher genomic p-value was selected for inclusion. Ancestry informative markers (AIMs) were also included in the assay to help control for population structure.[30] These 98 markers have previously been reported [7]; they describe more than 97% of the genetic variation captured by principal components from genome-wide genotyping data in the Standardbred breed.

### Variant analysis

#### Mixed model association analysis

Genotyping data from the Sequenom assay were pruned using default parameters in GEMMA (MAF < 1%, missingness <95%). The mixed model included a sex covariate (-c) and a relatedness matrix constructed from the AIMs (-gk 2). All three possible frequentist tests were calculated as described under **Genome-Wide Association (GWA) Analysis**. Statistical significance was set at p<0.05 with a Bonferroni correction for multiple testing.

#### Random forest classification analysis

Genotyping data from the Sequenom assay from 659 horses with race records (see **Sequenom Assay Discovery Cohort**) were subjected to random forest classification analysis using the ‘randomForest’ function of the ‘randomForest’ package in R.[31] The default parameters were used for number of trees (500) and number of variables tried at each split (√*n*, where n is the total number of variables). SNPs were pruned for missingness <90% prior to analysis. These data were subsequently subjected to 10-fold cross-validation using linear discriminate analysis to estimate misclassification error using the ‘errorest’ function of the ‘ipred’ package in R.[32] To determine the hierarchical organization of the most informative SNPs identified by random forest analysis, a conditional inference tree was constructed using the ‘ctree’ function of the ‘randomForest’ package.

#### Validation of predictive algorithm

As described under **Validation Cohort,** 166 independently sampled Standardbreds were genotyped on the custom Sequenom assay, and the genotypes at the SNPs included in the conditional inference tree were extracted for analysis. Horses were classified as pacers or trotters, observing the hierarchical relationships of the seven predictive SNPs as dictated by the conditional inference tree, by an individual blinded to their true gait status. Subsequently, the predicted gait was compared to the true gait, and sensitivity, specificity, and overall accuracy of the prediction algorithm was calculated.

## Supporting information

S1 Table SNPs from GEMMA mixed model analysis in 542 standardbred pacers and trotters that reached genome-wide significance. (DOCX)

S2 Table Regions of interest identified from pooled whole-genome sequencing in 20 pacers and 20 trotters. (DOCX)

S3 Table SNPs called within the regions of interest in S2 Table. (DOCX)

S4 Table Summary of SNPs putatively associated with gait that were selected for inclusion in the sequenom assay. (DOCX)

S5 Table Summary of statistically significant SNPs from GEMMA mixed model analysis in 720 standardbred pacers and trotters genotyped on the custom sequenom assay. (DOCX)

S1 Fig Diagram of footfall pattern of the trot, pace, and tölt. (DOCX)

S2 Fig MDS plot of GWA population. (DOCX)
