# Supplementary material for: Identification and validation of genetic variants predictive of gait in standardbred horses
Source: PLoS Genet. 2019 May 28;15(5):e1008146. doi: 10.1371/journal.pgen.1008146 (PMC6555539; doi:10.1371/journal.pgen.1008146)
Supplement: S1 Table — (DOCX) [file pgen.1008146.s001.docx]

**Supplemental Table 1:** Single nucleotide polymorphisms (SNPs) from GEMMA mixed model analysis in 542 Standardbred pacers and trotters (sex and origin covariates) that reached genome-wide significance (p < 1.44 x 10^-6^, as determined by the likelihood ratio test; in **bold**), or were moderately associated with gait (p < 1 x 10^-5^). After pruning, analysis included 62,901 SNPs. Uncorrected p-values are presented for the Wald test, the Likelihood ratio test (lrt) and the Score test. CHR = chromosome. SNPs were subsequently remapped to EquCab3.0 using BLAST (NCBI).

| RANK | CHR | EquCab2 | EquCab3 | p_wald | p_lrt | p_score |
| --- | --- | --- | --- | --- | --- | --- |
| 1 | 17 | 60554458 | 60446633 | 4.7E-11 | **3.9E-11** | 2.1E-10 |
| 2 | 1 | 18091577 | 18209783 | 1.1E-09 | **9.1E-10** | 3.2E-09 |
| 3 | 6 | 7372690 | 7146326 | 1.1E-09 | **9.6E-10** | 3.4E-09 |
| 4 | 23 | 14650375 | 14017592 | 2.3E-09 | **1.9E-09** | 6.3E-09 |
| 5 | 6 | 7000504 | 6774154 | 3.8E-08 | **3.3E-08** | 7.8E-08 |
| 6 | 17 | 60503138 | 60393995 | 5.9E-08 | **5.2E-08** | 1.2E-07 |
| 7 | 17 | 60523882 | 60414731 | 5.9E-08 | **5.2E-08** | 1.2E-07 |
| 8 | 17 | 40999944 | 40904434 | 1.4E-07 | **1.3E-07** | 2.6E-07 |
| 9 | 17 | 41905502 | 41810361 | 2.2E-07 | **2.0E-07** | 3.9E-07 |
| 10 | 1 | 155226154 | 156822538 | 2.2E-07 | **2.0E-07** | 3.9E-07 |
| 11 | 25 | 2021044 | 2054596 | 2.4E-07 | **2.2E-07** | 4.2E-07 |
| 12 | 17 | 28460851 | 28363078 | 3.1E-07 | **2.7E-07** | 5.3E-07 |
| 13 | 17 | 36291973 | 36195182 | 3.1E-07 | **2.8E-07** | 5.3E-07 |
| 14 | 19 | 24644810 | 27049559 | 5.2E-07 | **4.6E-07** | 8.5E-07 |
| 15 | 31 | 18200337 | 18243722 | 7.9E-07 | **7.1E-07** | 1.3E-06 |
| 16 | 9 | 76324169 | 78430548 | 9.1E-07 | **8.2E-07** | 1.4E-06 |
| 17 | 31 | 18194086 | 18237471 | 1.1E-06 | **9.6E-07** | 1.7E-06 |
| 18 | 1 | 55259288 | 55705873 | 1.6E-06 | **1.4E-06** | 2.3E-06 |
| 19 | 2 | 19755735 | 19803462 | 1.6E-06 | **1.4E-06** | 2.4E-06 |
| 20 | 31 | 18163586 | 18206959 | 1.7E-06 | 1.5E-06 | 2.5E-06 |
| 21 | 31 | 18207378 | 18250763 | 1.7E-06 | 1.5E-06 | 2.5E-06 |
| 22 | 31 | 18263790 | 18307152 | 1.7E-06 | 1.5E-06 | 2.5E-06 |
| 23 | 1 | 70111131 | 70657100 | 2.3E-06 | 2.1E-06 | 3.3E-06 |
| 24 | 31 | 18083836 | 18127380 | 2.3E-06 | 2.1E-06 | 3.4E-06 |
| 25 | 17 | 25963835 | 25865544 | 3.1E-06 | 2.88E-06 | 4.5E-06 |
| 26 | 31 | 5160203 | 5504779 | 3.2E-06 | 2.9E-06 | 4.6E-06 |
| 27 | 10 | 60356610 | 61493135 | 3.6E-06 | 3.3E-06 | 5.2E-06 |
| 28 | 25 | 16689693 | 17098831 | 3.9E-06 | 3.6E-06 | 5.5E-06 |
| 29 | 23 | 26109618 | 25502464 | 4.3E-06 | 3.9E-06 | 6.0E-06 |
| 30 | 1 | 43852372 | 44193733 | 4.3E-06 | 3.9E-06 | 6.0E-06 |
| 31 | 2 | 17532819 | 17580102 | 5.1E-06 | 4.7E-06 | 7.1E-06 |
| 32 | 20 | 43319164 | 44218220 | 5.3E-06 | 4.9E-06 | 7.4E-06 |
| 33 | 2 | 1673079 | 1685724 | 5.5E-06 | 5.0E-06 | 7.5E-06 |
| 34 | 2 | 1674613 | 1687258 | 5.5E-06 | 5.0E-06 | 7.5E-06 |
| 35 | 1 | 155323247 | 156919611 | 5.7E-06 | 5.2E-06 | 7.9E-06 |
| 36 | 3 | 46116569 | 47496680 | 5.8E-06 | 5.3E-06 | 8.0E-06 |
| 37 | 1 | 38250747 | 38500240 | 5.8E-06 | 5.4E-06 | 8.1E-06 |
| 38 | 2 | 1919026 | 1931765 | 5.9E-06 | 5.4E-06 | 8.1E-06 |
| 39 | 17 | 60468135 | 60358988 | 5.9E-06 | 5.4E-06 | 8.2E-06 |
| 40 | 17 | 60468732 | 60359585 | 6.1E-06 | 5.6E-06 | 8.4E-06 |
| 41 | 1 | 39331337 | 39667373 | 6.3E-06 | 5.8E-06 | 8.6E-06 |
| 42 | 5 | 88412158 | 85395371 | 6.9E-06 | 6.4E-06 | 9.4E-06 |
| 43 | 10 | 5679757 | 5801222 | 7.1E-06 | 6.5E-06 | 9.7E-06 |
| 44 | 19 | 30643317 | 33175656 | 7.4E-06 | 6.8E-06 | 1.0E-05 |
| 45 | 2 | 2150549 | 2163319 | 7.9E-06 | 7.2E-06 | 1.1E-05 |
| 46 | 31 | 17759802 | 17803543 | 7.9E-06 | 7.3E-06 | 1.1E-05 |
| 47 | 31 | 18562346 | 18607605 | 8.1E-06 | 7.5E-06 | 1.1E-05 |
| 48 | 31 | 5160132 | 5504850 | 8.2E-06 | 7.5E-06 | 1.1E-05 |
| 49 | 2 | 1543256 | 1538800 | 8.5E-06 | 7.8E-06 | 1.2E-05 |
| 50 | 9 | 78229420 | 80336648 | 9.1E-06 | 8.3E-06 | 1.2E-05 |
| 51 | 31 | \| 18266165 \| \| --- \| | \| 18309527 \| \| --- \| | 9.1E-06 | 8.4E-06 | 1.2E-05 |
| 52 | 12 | 2155432 | 2163996 | 9.8E-06 | 9.0E-06 | 1.3E-05 |
| 53 | 1 | 18091709 | 18209915 | 1.0E-05 | 9.2E-06 | 1.3E-05 |
| 54 | 30 | \| 15254104 \| \| --- \| | \| 16089829 \| \| --- \| | \| 1.0E-05 \| \| --- \| | 9.4E-06 | 1.4E-05 |
| 55 | 1 | \| 5649300 \| \| --- \| | \| 5676220 \| \| --- \| | \| 1.0E-05 \| \| --- \| | 9.6E-06 | \| 1.4E-05 \| \| --- \| |
| 56 | 19 | 28402195 | 30800326 | 1.1E-05 | 9.9E-06 | 1.4E-05 |
