# Supplementary material for: Identification and validation of genetic variants predictive of gait in standardbred horses
Source: PLoS Genet. 2019 May 28;15(5):e1008146. doi: 10.1371/journal.pgen.1008146 (PMC6555539; doi:10.1371/journal.pgen.1008146)
Supplement: S2 Table — (DOCX) [file pgen.1008146.s002.docx]

**Supplemental Table 2:** Regions of interest identified from pooled whole-genome sequencing in 20 pacers and 20 trotters. Regions were either highly differentiated between pacers and trotters (F_ST_ ≥ 0.35) or had both low pool heterozygosity (Hp < 0.1) in one of the groups and high differentiation (F_ST_ ≥ 0.30). CHR = chromosome; BP = base pair; nSNPs = number of single nucleotide polymorphisms; Het = heterozygosity; P = pacers; T = trotters. Intervals were remapped to EquCab3.0 using BLAST (NCBI); these are shown in parentheses beneath the EquCab2 locations in the table. There was one interval that did not map to the new reference due to an alignment gap.

| Criteria | CHR | BP  start | BP  end | nSNPs | Het P | Het (T) | FST  P vs. T |
| --- | --- | --- | --- | --- | --- | --- | --- |
| FST | 1 | 38550001  (38799648) | 38600000  (38849603) | 137 | 0.37 | 0.15 | 0.36 |
| FST | 1 | 38800001  (39050918) | 38850000  (39100850) | 162 | 0.41 | 0.16 | 0.35 |
| FST+Het (T) | 1 | 106900001  (107814628) | 106950000  (107864591) | 323 | 0.40 | 0.09 | 0.34 |
| FST+Het (T) | 1 | 106925001  (107839628) | 106975000  (107889648) | 229 | 0.42 | 0.62 | 0.35 |
| FST | 3 | 2475001  (2607721) | 2525000  (2657637) | 521 | 0.35 | 0.18 | 0.35 |
| FST | 3 | 36325001  (37044709) | 36375000  (37094434) | 49 | 0.12 | 0.45 | 0.37 |
| FST | 3 | 52275001  (53748519) | 52325000  (53798521) | 306 | 0.20 | 0.41 | 0.35 |
| FST+Het (T) | 4 | 8975001  (8975583) | 9025000  (9025603) | 335 | 0.27 | 0.10 | 0.33 |
| FST | 4 | 9050001  (9050617) | 9100000  (9100616) | 546 | 0.31 | 0.22 | 0.45 |
| FST | 4 | 9075001  (9075618) | 9125000  (9125621) | 659 | 0.29 | 0.23 | 0.42 |
| FST | 5 | 55275001  (51854606) | 55325000  (51904614) | 206 | 0.31 | 0.26 | 0.38 |
| FST | 5 | 55300001  (51879605) | 55350000  (51929614) | 186 | 0.33 | 0.24 | 0.37 |
| FST | 5 | 61125001  (57707180) | 61175000  (57757172) | 362 | 0.17 | 0.35 | 0.38 |
| FST | 5 | 66150001  (62979383) | 66200000  (63029378) | 187 | 0.31 | 0.17 | 0.40 |
| FST | 5 | 66175001  (63004381) | 66225000  (63054378) | 220 | 0.32 | 0.21 | 0.38 |
| FST | 6 | 81275001  (82439170) | 81325000  (82487568) | 164 | 0.13 | 0.36 | 0.37 |
| FST+Het (P) | 6 | 81525001  (82688791) | 81575000  (82738800) | 101 | 0.07 | 0.34 | 0.38 |
| FST+Het (P) | 6 | 81625001  (82788817) | 81675000  (82838816) | 93 | 0.08 | 0.37 | 0.31 |
| FST | 9 | 29125001  (29906127) | 29175000  (29956151) | 280 | 0.25 | 0.40 | 0.38 |
| FST | 9 | 29150001  (29931142) | 29200000  (29981143) | 282 | 0.25 | 0.40 | 0.37 |
| FST | 9 | 29175001  (29956153) | 29225000  (30006143) | 253 | 0.23 | 0.40 | 0.35 |
| FST+Het (P) | 9 | 44550001  (46206312) | 44600000  (46256398) | 109 | 0.05 | 0.39 | 0.34 |
| FST+Het (P) | 9 | 44575001  (46231360) | 44625000  (46281356) | 86 | 0.05 | 0.39 | 0.36 |
| FST+Het (P) | 11 | 29475001  (29734805) | 29525000  (29784776) | 261 | 0.08 | 0.44 | 0.32 |
| FST | 11 | 29500001  (29759805) | 29550000  (29809785) | 490 | 0.11 | 0.41 | 0.38 |
| FST | 11 | 29525001  (29784778) | 29575000  (29835462) | 469 | 0.20 | 0.36 | 0.38 |
| FST+Het (P) | 11 | 31275001  (31573660) | 31325000  (31663818) | 171 | 0.06 | 0.38 | 0.32 |
| FST+Het (P) | 11 | 31450001  (31748719) | 31500000  (31798714) | 75 | 0.07 | 0.38 | 0.30 |
| FST+Het (P) | 11 | 36625001  (36920225) | 36675000  (36970186) | 74 | 0.04 | 0.39 | 0.33 |
| FST+Het (P) | 11 | 36650001  (36945244) | 36700000  (36995189) | 116 | 0.02 | 0.38 | 0.32 |
| FST+Het (P) | 11 | 36675001  (36970188) | 36725000  (37020174) | 114 | 0.02 | 0.37 | 0.32 |
| FST+Het (P) | 11 | 36700001  (36995191) | 36750000  (37045173) | 79 | 0.02 | 0.33 | 0.33 |
| FST+Het (P) | 11 | 36725001  (37020176) | 36775000  (37070168) | 78 | 0.04 | 0.32 | 0.37 |
| FST+Het (P) | 11 | 36750001  (37045175) | 36800000  (37095167) | 130 | 0.03 | 0.37 | 0.48 |
| FST+Het (P) | 11 | 36775001  (37070170) | 36825000  (37120167) | 137 | 0.04 | 0.36 | 0.47 |
| FST+Het (T) | 12 | 14200001  (15204395) | 14250000  (15240003) | 35 | 0.34 | 0.01 | 0.37 |
| FST | 12 | 16250001  (19680380) | 16300000  (19730386) | 366 | 0.16 | 0.42 | 0.36 |
| FST | 12 | 16375001  (19805396) | 16425000  (19855352) | 379 | 0.18 | 0.37 | 0.38 |
| FST | 12 | 16400001  (19830345) | 16450000  (19880356) | 351 | 0.17 | 0.36 | 0.36 |
| FST | 14 | 1350001  (591156) | 1400000  (641174) | 472 | 0.30 | 0.23 | 0.40 |
| FST | 14 | 1375001  (616173) | 1425000  (666173) | 459 | 0.33 | 0.17 | 0.50 |
| FST | 14 | 1400001  (641176) | 1450000  (691173) | 289 | 0.32 | 0.18 | 0.51 |
| FST | 14 | 1475001  (716173) | 1525000  (766137) | 596 | 0.39 | 0.25 | 0.36 |
| FST | 14 | 1500001  (741173) | 1550000  (791335) | 745 | 0.42 | 0.20 | 0.39 |
| FST | 14 | 1525001  (766139) | 1575000  (816335) | 436 | 0.41 | 0.17 | 0.40 |
| FST+Het (T) | 14 | 5450001  (4684550) | 5500000  (4734581) | 164 | 0.38 | 0.03 | 0.31 |
| FST | 15 | 10100001  (10371492) | 10150000  (10421490) | 391 | 0.40 | 0.19 | 0.35 |
| FST | 16 | 59350001  (60929845) | 59400000  (60979776) | 163 | 0.29 | 0.35 | 0.37 |
| FST | 17 | 50950001  (50828482) | 51000000  (50878481) | 99 | 0.33 | 0.10 | 0.38 |
| FST+Het (T) | 17 | 50975001  (50853483) | 51025000  (50903481) | 175 | 0.34 | 0.08 | 0.37 |
| FST+Het (T) | 17 | 51000001  (50878483) | 51050000  (50928481) | 215 | 0.32 | 0.09 | 0.30 |
| FST | 17 | 61700001  (61592627) | 61750000  (61642637) | 213 | 0.17 | 0.37 | 0.37 |
| FST | 17 | 65625001  (65526031) | 65675000  (65576028) | 217 | 0.32 | 0.24 | 0.36 |
| FST+Het (T) | 18 | 75675001  (not aligned) | 75725000  (not aligned) | 4 | 0.26 | 0.09 | 0.50 |
| FST+Het (T) | 20 | 25100001  (25963160) | 25150000  (26013157) | 592 | 0.44 | 0.08 | 0.35 |
| FST+Het (T) | 20 | 27675001  (28579429) | 27725000  (28629392) | 1095 | 0.41 | 0.05 | 0.45 |
| FST | 20 | 27700001  (28604393) | 27750000  (28654392) | 912 | 0.37 | 0.10 | 0.42 |
| FST | 20 | 27725001  (28629394) | 27775000  (28679390) | 720 | 0.35 | 0.19 | 0.37 |
| FST | 20 | 46925001  (47919318) | 46975000  (47969368) | 330 | 0.36 | 0.24 | 0.37 |
| FST | 20 | 47050001  (48044375) | 47100000  (48094371) | 241 | 0.25 | 0.27 | 0.42 |
| FST | 20 | 47075001  (48069375) | 47125000  (48119380) | 224 | 0.26 | 0.29 | 0.41 |
| FST | 23 | 14600001  (13967204) | 14650000  (14017217) | 158 | 0.30 | 0.18 | 0.36 |
| FST | 23 | 14625001  (13992206) | 14675000  (14042211) | 186 | 0.31 | 0.21 | 0.36 |
| FST | 23 | 20625001  (20008730) | 20675000  (20058818) | 371 | 0.23 | 0.35 | 0.36 |
| FST+Het (P) | 23 | 50450001  (50260812) | 50500000  (50310654) | 43 | 0.05 | 0.41 | 0.32 |
| FST | 24 | 6700001  (6562469) | 6750000  (6612429) | 265 | 0.13 | 0.40 | 0.37 |
| FST | 24 | 10275001  (10131967) | 10325000  (10181822) | 367 | 0.20 | 0.33 | 0.36 |
| FST | 25 | 3650001  (3697137) | 3700000  (3747133) | 409 | 0.23 | 0.29 | 0.40 |
| FST | 25 | 3675001  (3722137) | 3725000  (3772118) | 470 | 0.24 | 0.27 | 0.41 |
| FST | 25 | 3700001  (3747135) | 3750000  (3791181) | 474 | 0.24 | 0.28 | 0.41 |
| FST | 25 | 3800001  (3848533) | 3850000  (3897404) | 397 | 0.21 | 0.28 | 0.41 |
| FST | 25 | 3825001  (3873533) | 3875000  (3923569) | 685 | 0.21 | 0.30 | 0.48 |
| FST | 25 | 11775001  (11824176) | 11825000  (11874178) | 283 | 0.18 | 0.21 | 0.43 |
| FST | 25 | 11800001  (11849161) | 11850000  (11900037) | 361 | 0.18 | 0.22 | 0.37 |
| FST | 25 | 15025001  (15424701) | 15075000  (15476571) | 192 | 0.27 | 0.21 | 0.37 |
| FST+Het (T) | 29 | 3175001  (4189335) | 3225000  (4239637) | 275 | 0.42 | 0.09 | 0.38 |
| FST+Het (T) | 29 | 3250001  (4264639) | 3300000  (4314636) | 113 | 0.36 | 0.06 | 0.32 |
| FST+Het (T) | 29 | 3275001  (4289638) | 3325000  (4339638) | 147 | 0.40 | 0.007 | 0.34 |
| FST+Het (T) | 29 | 3300001  (4314638) | 3350000  (4364671) | 282 | 0.40 | 0.007 | 0.35 |
| FST+Het (T) | 29 | 3325001  (4339640) | 3375000  (4389684) | 303 | 0.39 | 0.007 | 0.37 |
| FST+Het (T) | 29 | 3350001  (4364673) | 3400000  (4414655) | 172 | 0.38 | 0.02 | 0.35 |
| FST+Het (T) | 29 | 3400001  (4414657) | 3450000  (4464629) | 227 | 0.32 | 0.01 | 0.30 |
| FST+Het (T) | 29 | 3425001  (4439662) | 3475000  (4489586) | 352 | 0.39 | 0.04 | 0.39 |
| FST+Het (T) | 29 | 10075001  (11098082) | 10125000  (11148079) | 310 | 0.39 | 0.10 | 0.34 |
| FST+Het (T) | 30 | 14075001  (14910612) | 14125000  (14960611) | 209 | 0.30 | 0.10 | 0.32 |
| FST | 30 | 14900001  (15735334) | 14950000  (15785653) | 382 | 0.38 | 0.11 | 0.37 |
| FST | 30 | 14925001  (15760648) | 14975000  (15810653) | 346 | 0.34 | 0.12 | 0.38 |
| FST | 30 | 14950001  (15785655) | 15000000  (15835659) | 283 | 0.32 | 0.13 | 0.35 |
| FST+Het (T) | 30 | 15075001  (15910678) | 15125000  (15960648) | 208 | 0.28 | 0.10 | 0.33 |
