# Supplementary material for: Identification and validation of genetic variants predictive of gait in standardbred horses
Source: PLoS Genet. 2019 May 28;15(5):e1008146. doi: 10.1371/journal.pgen.1008146 (PMC6555539; doi:10.1371/journal.pgen.1008146)
Supplement: S3 Table — (DOCX) [file pgen.1008146.s003.docx]

**Supplemental Table 3:** SNPs called within regions of interest in **Supplemental Table 2**. Gene annotations are from Ensembl (Equ Cab 2; GCA_000002305.1). Genes that were predicted, but unnamed, in Ensembl were identified via BLAST (https://blast.ncbi.nlm.nih.gov/Blast.cgi) when possible. CHR = chromosome; BP = base pair.

| CHR | BP | GENE | LOCATION | NOTES |
| --- | --- | --- | --- | --- |
| 1 | 38553622 | KIF20B | downstream | ~20kb downstream |
| 1 | 38562278 | KIF20B | downstream | ~10kb downstream |
| 1 | 38562941 | KIF20B | downstream | ~10kb downstream |
| 1 | 38563255 | KIF20B | downstream | ~10kb downstream |
| 1 | 38573734 | KIF20B | intron | ~130bp from jxn |
| 1 | 38578677 | KIF20B | intron | <1kb from jxn |
| 1 | 38592096 | KIF20B | intron | ~200bp from jxn |
| 1 | 38592686 | KIF20B | intron | ~250bp from jxn |
| 1 | 38592960 | KIF20B | intron | ~150bp from jxn |
| 1 | 38597071 | KIF20B | intron | middle of large intron |
| 1 | 38599156 | KIF20B | intron | ~800bp from jxn |
| 1 | 38599371 | KIF20B | intron | 600bp from jxn |
| 1 | 38819165 |  | intergenic |  |
| 1 | 38836285 |  | intergenic |  |
| 1 | 38837055 |  | intergenic |  |
| 1 | 38837069 |  | intergenic |  |
| 1 | 38837071 |  | intergenic |  |
| 1 | 38837073 |  | intergenic |  |
| 1 | 38837077 |  | intergenic |  |
| 1 | 38845979 |  | intergenic |  |
| 1 | 106883816 |  | intergenic |  |
| 1 | 106928193 |  | intergenic |  |
| 1 | 106928205 |  | intergenic |  |
| 3 | 2478863 | CBLN1 | intron | middle of large intron |
| 3 | 2478864 | CBLN1 | intron | middle of large intron |
| 3 | 2479058 | CBLN1 | intron | middle of large intron |
| 3 | 2479065 | CBLN1 | intron | middle of large intron |
| 3 | 2485799 | CBLN1 | intron | middle of large intron |
| 3 | 2489598 | CBLN1 | intron | middle of large intron |
| 3 | 2492536 | CBLN1 | intron | middle of large intron |
| 3 | 2495373 | CBLN1 | intron | middle of large intron |
| 3 | 2495399 | CBLN1 | intron | middle of large intron |
| 3 | 2501929 | CBLN1 | intron | middle of large intron |
| 3 | 2503495 | CBLN1 | intron | middle of large intron |
| 3 | 2504968 | CBLN1 | intron | middle of large intron |
| 3 | 2506253 | CBLN1 | intron | middle of large intron |
| 3 | 2506325 | CBLN1 | intron | middle of large intron |
| 3 | 2506748 | CBLN1 | intron | middle of large intron |
| 3 | 2507778 | CBLN1 | intron | middle of large intron |
| 3 | 2507781 | CBLN1 | intron | middle of large intron |
| 3 | 2508450 | CBLN1 | intron | middle of large intron |
| 3 | 2511003 | CBLN1 | intron | middle of large intron |
| 3 | 2512162 | CBLN1 | intron | middle of large intron |
| 3 | 2512762 | CBLN1 | intron | middle of large intron |
| 3 | 2518412 | CBLN1 | intron | middle of large intron |
| 3 | 2521133 | CBLN1 | intron | middle of large intron |
| 3 | 2521213 | CBLN1 | intron | middle of large intron |
| 3 | 2521217 | CBLN1 | intron | middle of large intron |
| 3 | 2521535 | CBLN1 | intron | middle of large intron |
| 3 | 2521561 | CBLN1 | intron | middle of large intron |
| 3 | 2523989 | CBLN1 | intron | middle of large intron |
| 3 | 52318025 |  | intergenic |  |
| 4 | 8955197 |  | intergenic |  |
| 4 | 8956182 |  | intergenic |  |
| 4 | 8956988 |  | intergenic |  |
| 4 | 8957134 |  | intergenic |  |
| 4 | 8957435 |  | intergenic |  |
| 4 | 8957441 |  | intergenic |  |
| 4 | 8957485 |  | intergenic |  |
| 4 | 8957527 |  | intergenic |  |
| 4 | 8957866 |  | intergenic |  |
| 4 | 8957970 |  | intergenic |  |
| 4 | 8958835 |  | intergenic |  |
| 4 | 8959008 |  | intergenic |  |
| 4 | 8959886 |  | intergenic |  |
| 4 | 8960906 |  | intergenic |  |
| 4 | 8960933 |  | intergenic |  |
| 4 | 8960940 |  | intergenic |  |
| 4 | 8962032 |  | intergenic |  |
| 4 | 8962035 |  | intergenic |  |
| 4 | 8963139 |  | intergenic |  |
| 4 | 8972922 |  | intergenic |  |
| 4 | 8974199 | ENSECAG00000002028 | downstream | ~3kb downstream |
| 4 | 8980469 | ENSECAG00000002028 | upstream | ~3kb upstream |
| 4 | 8983343 |  | intergenic |  |
| 4 | 8983626 |  | intergenic |  |
| 4 | 8987092 |  | intergenic |  |
| 4 | 8987743 |  | intergenic |  |
| 4 | 8989774 |  | intergenic |  |
| 4 | 8990160 |  | intergenic |  |
| 4 | 8995099 | ENSECAG00000017866 | downstream | ~1kb downstream |
| 4 | 8995335 | ENSECAG00000017866 | downstream | ~1kb downstream |
| 4 | 8995628 | ENSECAG00000017866 | downstream | ~1kb downstream |
| 4 | 8995695 | ENSECAG00000017866 | downstream | ~1kb downstream |
| 4 | 8996339 | ENSECAG00000017866 | downstream | 400bp downstream |
| 4 | 8996364 | ENSECAG00000017866 | downstream | 400bp downstream |
| 4 | 8996598 | ENSECAG00000017866 | downstream | 100bp downstream |
| 4 | 8996710 | ENSECAG00000017866 | downstream | 5bp downstream |
| 4 | 8996716 | ENSECAG00000017866 | exon | exon 2 |
| 4 | 8996717 | ENSECAG00000017866 | exon | exon 2 |
| 4 | 8996753 | ENSECAG00000017866 | exon | exon 2 |
| 4 | 8996922 | ENSECAG00000017866 | exon | exon 2 |
| 4 | 8997798 | ENSECAG00000017866 | upstream | 600bp upstream |
| 4 | 8997801 | ENSECAG00000017866 | upstream | 600bp upstream |
| 4 | 8997814 | ENSECAG00000017866 | upstream | 600bp upstream |
| 4 | 8997900 | ENSECAG00000017866 | upstream | 700bp upstream |
| 4 | 8997934 | ENSECAG00000017866 | upstream | 700bp upstream |
| 4 | 8998441 | ENSECAG00000017866 | upstream | ~1kb upstream |
| 4 | 8998562 | ENSECAG00000017866 | upstream | ~1kb upstream |
| 4 | 8998566 | ENSECAG00000017866 | upstream | ~1kb upstream |
| 4 | 8998594 | ENSECAG00000017866 | upstream | ~1kb upstream |
| 4 | 8998595 | ENSECAG00000017866 | upstream | ~1kb upstream |
| 4 | 8998596 | ENSECAG00000017866 | upstream | ~1kb upstream |
| 4 | 8998883 | ENSECAG00000017866 | upstream | ~1kb upstream |
| 4 | 8999055 | ENSECAG00000017866 | upstream | ~2kb upstream |
| 4 | 8999107 | ENSECAG00000017866 | upstream | ~2kb upstream |
| 4 | 8999837 | ENSECAG00000017866 | upstream | ~2kb upstream |
| 4 | 8999971 | ENSECAG00000017866 | upstream | ~2kb upstream |
| 4 | 9000695 | ENSECAG00000017866 | upstream | ~3kb upstream |
| 4 | 9001202 | ENSECAG00000017866 | upstream | ~4kb upstream |
| 4 | 9001547 | ENSECAG00000017866 | upstream | ~4kb upstream |
| 4 | 9003830 |  | intergenic |  |
| 4 | 9004962 |  | intergenic |  |
| 4 | 9005175 |  | intergenic |  |
| 4 | 9007488 |  | intergenic |  |
| 4 | 9007823 |  | intergenic |  |
| 4 | 9008489 |  | intergenic |  |
| 4 | 9010223 |  | intergenic |  |
| 4 | 9012516 |  | intergenic |  |
| 4 | 9014221 |  | intergenic |  |
| 4 | 9016426 |  | intergenic |  |
| 4 | 9016833 |  | intergenic |  |
| 4 | 9016867 |  | intergenic |  |
| 4 | 9018336 |  | intergenic |  |
| 4 | 9018741 |  | intergenic |  |
| 4 | 9020578 |  | intergenic |  |
| 4 | 9020579 |  | intergenic |  |
| 4 | 9021901 |  | intergenic |  |
| 4 | 9023318 |  | intergenic |  |
| 4 | 9026877 |  | intergenic |  |
| 4 | 9028735 |  | intergenic |  |
| 4 | 9028758 |  | intergenic |  |
| 4 | 9028783 |  | intergenic |  |
| 4 | 9028819 |  | intergenic |  |
| 4 | 9028827 |  | intergenic |  |
| 4 | 9028901 |  | intergenic |  |
| 4 | 9028937 |  | intergenic |  |
| 4 | 9029196 |  | intergenic |  |
| 4 | 9029216 |  | intergenic |  |
| 4 | 9029237 |  | intergenic |  |
| 4 | 9029307 |  | intergenic |  |
| 4 | 9029309 |  | intergenic |  |
| 4 | 9029347 |  | intergenic |  |
| 4 | 9029509 |  | intergenic |  |
| 4 | 9031644 |  | intergenic |  |
| 4 | 9031676 |  | intergenic |  |
| 4 | 9032458 |  | intergenic |  |
| 4 | 9032824 |  | intergenic |  |
| 4 | 9042626 |  | intergenic |  |
| 4 | 9054167 |  | intergenic |  |
| 4 | 9063166 |  | intergenic |  |
| 4 | 9066542 |  | intergenic |  |
| 4 | 9077261 |  | intergenic |  |
| 4 | 9083538 |  | intergenic |  |
| 4 | 9087152 |  | intergenic |  |
| 4 | 9087394 |  | intergenic |  |
| 4 | 9087405 |  | intergenic |  |
| 4 | 9087409 |  | intergenic |  |
| 4 | 9087413 |  | intergenic |  |
| 4 | 9087669 |  | intergenic |  |
| 4 | 9088713 |  | intergenic |  |
| 4 | 9088749 |  | intergenic |  |
| 4 | 9088754 |  | intergenic |  |
| 4 | 9090378 |  | intergenic |  |
| 4 | 9090552 |  | intergenic |  |
| 4 | 9090554 |  | intergenic |  |
| 4 | 9090655 |  | intergenic |  |
| 4 | 9090662 |  | intergenic |  |
| 4 | 9090668 |  | intergenic |  |
| 4 | 9090681 |  | intergenic |  |
| 4 | 9090730 |  | intergenic |  |
| 4 | 9090822 |  | intergenic |  |
| 4 | 9090848 |  | intergenic |  |
| 4 | 9090942 |  | intergenic |  |
| 4 | 9090964 |  | intergenic |  |
| 4 | 9090965 |  | intergenic |  |
| 4 | 9091038 |  | intergenic |  |
| 4 | 9091041 |  | intergenic |  |
| 4 | 9091075 |  | intergenic |  |
| 4 | 9091107 |  | intergenic |  |
| 4 | 9091117 |  | intergenic |  |
| 4 | 9091120 |  | intergenic |  |
| 4 | 9091283 |  | intergenic |  |
| 4 | 9091499 |  | intergenic |  |
| 4 | 9091500 |  | intergenic |  |
| 4 | 9091516 |  | intergenic |  |
| 4 | 9091863 |  | intergenic |  |
| 4 | 9092154 |  | intergenic |  |
| 4 | 9092167 |  | intergenic |  |
| 4 | 9092168 |  | intergenic |  |
| 4 | 9092420 |  | intergenic |  |
| 4 | 9092424 |  | intergenic |  |
| 4 | 9092426 |  | intergenic |  |
| 4 | 9092493 |  | intergenic |  |
| 4 | 9092501 |  | intergenic |  |
| 4 | 9092517 |  | intergenic |  |
| 4 | 9092528 |  | intergenic |  |
| 4 | 9092535 |  | intergenic |  |
| 4 | 9092536 |  | intergenic |  |
| 4 | 9092541 |  | intergenic |  |
| 4 | 9092550 |  | intergenic |  |
| 4 | 9092557 |  | intergenic |  |
| 4 | 9092612 |  | intergenic |  |
| 4 | 9092620 |  | intergenic |  |
| 4 | 9092663 |  | intergenic |  |
| 4 | 9092672 |  | intergenic |  |
| 4 | 9092754 |  | intergenic |  |
| 4 | 9092773 |  | intergenic |  |
| 4 | 9092866 |  | intergenic |  |
| 4 | 9092908 |  | intergenic |  |
| 4 | 9092936 |  | intergenic |  |
| 4 | 9093028 |  | intergenic |  |
| 4 | 9093049 |  | intergenic |  |
| 4 | 9093289 |  | intergenic |  |
| 4 | 9093291 |  | intergenic |  |
| 4 | 9093295 |  | intergenic |  |
| 4 | 9093298 |  | intergenic |  |
| 4 | 9093371 |  | intergenic |  |
| 4 | 9093376 |  | intergenic |  |
| 4 | 9093401 |  | intergenic |  |
| 4 | 9093409 |  | intergenic |  |
| 4 | 9093425 |  | intergenic |  |
| 4 | 9093427 |  | intergenic |  |
| 4 | 9093435 |  | intergenic |  |
| 4 | 9093733 |  | intergenic |  |
| 4 | 9093954 |  | intergenic |  |
| 4 | 9096565 |  | intergenic |  |
| 4 | 9096894 |  | intergenic |  |
| 4 | 9098356 |  | intergenic |  |
| 4 | 9098365 |  | intergenic |  |
| 4 | 9098379 |  | intergenic |  |
| 4 | 9098381 |  | intergenic |  |
| 4 | 9098530 |  | intergenic |  |
| 4 | 9098670 |  | intergenic |  |
| 4 | 9098703 |  | intergenic |  |
| 4 | 9098738 |  | intergenic |  |
| 4 | 9098759 |  | intergenic |  |
| 4 | 9098762 |  | intergenic |  |
| 4 | 9099801 |  | intergenic |  |
| 4 | 9099802 |  | intergenic |  |
| 4 | 9099803 |  | intergenic |  |
| 4 | 9099847 |  | intergenic |  |
| 4 | 9101245 |  | intergenic |  |
| 4 | 9101330 |  | intergenic |  |
| 4 | 9104503 |  | intergenic |  |
| 4 | 9105007 |  | intergenic |  |
| 4 | 9105325 |  | intergenic |  |
| 4 | 9106613 |  | intergenic |  |
| 4 | 9107819 |  | intergenic |  |
| 4 | 9109892 |  | intergenic |  |
| 4 | 9110389 |  | intergenic |  |
| 4 | 9110675 |  | intergenic |  |
| 4 | 9110791 |  | intergenic |  |
| 4 | 9110818 |  | intergenic |  |
| 4 | 9112845 |  | intergenic |  |
| 4 | 9116614 |  | intergenic |  |
| 4 | 9122606 |  | intergenic |  |
| 5 | 55286919 |  | intergenic | ~60kb from MAGI3 |
| 5 | 55291504 |  | intergenic | ~60kb from MAGI3 |
| 5 | 55291520 |  | intergenic | ~60kb from MAGI3 |
| 5 | 55291788 |  | intergenic | ~60kb from MAGI3 |
| 5 | 55293804 |  | intergenic | ~60kb from MAGI3 |
| 5 | 55294037 |  | intergenic | ~60kb from MAGI3 |
| 5 | 55294215 |  | intergenic | ~60kb from MAGI3 |
| 5 | 55294261 |  | intergenic | ~60kb from MAGI3 |
| 5 | 55294311 |  | intergenic | ~60kb from MAGI3 |
| 5 | 55297702 |  | intergenic | ~60kb from MAGI3 |
| 5 | 55300001 |  | intergenic | ~60kb from MAGI3 |
| 5 | 55301141 |  | intergenic | ~60kb from MAGI3 |
| 5 | 55302566 |  | intergenic | ~60kb from MAGI3 |
| 5 | 55304289 |  | intergenic | ~60kb from MAGI3 |
| 5 | 55304358 |  | intergenic | ~60kb from MAGI3 |
| 5 | 55317127 |  | intergenic | ~60kb from MAGI3 |
| 5 | 55318313 |  | intergenic | ~60kb from MAGI3 |
| 5 | 55318322 |  | intergenic | ~60kb from MAGI3 |
| 5 | 55318679 |  | intergenic | ~60kb from MAGI3 |
| 5 | 55320893 |  | intergenic | ~60kb from MAGI3 |
| 5 | 55321485 |  | intergenic | ~60kb from MAGI3 |
| 5 | 55322633 |  | intergenic | ~60kb from MAGI3 |
| 5 | 55326652 |  | intergenic | ~60kb from MAGI3 |
| 5 | 55327510 |  | intergenic | ~60kb from MAGI3 |
| 5 | 55333664 |  | intergenic | ~60kb from MAGI3 |
| 5 | 61126642 |  | intergenic |  |
| 5 | 61128939 |  | intergenic |  |
| 5 | 61128959 |  | intergenic |  |
| 5 | 61129436 |  | intergenic |  |
| 5 | 61130488 |  | intergenic |  |
| 5 | 61133992 |  | intergenic |  |
| 5 | 61136963 |  | intergenic |  |
| 5 | 61138434 |  | intergenic |  |
| 5 | 61138523 |  | intergenic |  |
| 5 | 61142264 |  | intergenic |  |
| 5 | 61142969 |  | intergenic |  |
| 5 | 61143062 |  | intergenic |  |
| 5 | 61144633 |  | intergenic |  |
| 5 | 61144680 |  | intergenic |  |
| 5 | 61145777 |  | intergenic |  |
| 5 | 61146280 |  | intergenic |  |
| 5 | 61146784 |  | intergenic |  |
| 5 | 61148875 |  | intergenic |  |
| 5 | 61149234 |  | intergenic |  |
| 5 | 61162570 |  | intergenic |  |
| 5 | 61162599 |  | intergenic |  |
| 5 | 61163608 |  | intergenic |  |
| 5 | 61163756 |  | intergenic |  |
| 5 | 61163875 |  | intergenic |  |
| 5 | 61163899 |  | intergenic |  |
| 5 | 61163978 |  | intergenic |  |
| 5 | 61164090 |  | intergenic |  |
| 5 | 61171791 |  | intergenic |  |
| 5 | 66151352 |  | intergenic | ~20kb from AGL |
| 5 | 66164707 |  | intergenic |  |
| 5 | 66168377 |  | intergenic |  |
| 5 | 66170038 |  | intergenic |  |
| 5 | 66171249 |  | intergenic |  |
| 5 | 66181399 | FRRS1 | upstream | ~20kb upstream |
| 5 | 66181445 | FRRS1 | upstream | ~20kb upstream |
| 5 | 66182723 | FRRS1 | upstream | ~20kb upstream |
| 5 | 66184792 | FRRS1 | upstream | ~15kb upstream |
| 5 | 66185718 | FRRS1 | upstream | ~15kb upstream |
| 5 | 66186194 | FRRS1 | upstream | ~15kb upstream |
| 5 | 66186777 | FRRS1 | upstream | ~15kb upstream |
| 5 | 66187039 | FRRS1 | upstream | ~15kb upstream |
| 5 | 66187303 | FRRS1 | upstream | ~15kb upstream |
| 5 | 66189804 | FRRS1 | upstream | ~10kb upstream |
| 5 | 66190553 | FRRS1 | upstream | ~10kb upstream |
| 5 | 66190626 | FRRS1 | upstream | ~10kb upstream |
| 5 | 66190733 | FRRS1 | upstream | ~10kb upstream |
| 5 | 66191023 | FRRS1 | upstream | ~10kb upstream |
| 5 | 66191193 | FRRS1 | upstream | ~10kb upstream |
| 5 | 66191646 | FRRS1 | upstream | 8kb upstream |
| 5 | 66191716 | FRRS1 | upstream | 8kb upstream |
| 5 | 66192232 | FRRS1 | upstream | 7kb upstream |
| 5 | 66193342 | FRRS1 | upstream | 6kb upstream |
| 5 | 66195600 | FRRS1 | upstream | 4kb upstream |
| 5 | 66196685 | FRRS1 | upstream | 3kb upstream |
| 5 | 66197898 | FRRS1 | upstream | 2kb upstream |
| 5 | 66198298 | FRRS1 | upstream | 2kb upstream |
| 5 | 66199061 | FRRS1 | upstream | ~800bp upstream |
| 5 | 66199239 | FRRS1 | upstream | ~550bp upstream |
| 5 | 66199885 | FRRS1 | exon | exon 1 |
| 5 | 66221515 | FRRS1 | exon | exon 9 |
| 6 | 81289305 | HMGA2 | intron | middle of large intron |
| 6 | 81289486 | HMGA2 | intron | middle of large intron |
| 6 | 81289563 | HMGA2 | intron | middle of large intron |
| 6 | 81290709 | HMGA2 | intron | middle of large intron |
| 6 | 81291276 | HMGA2 | intron | middle of large intron |
| 6 | 81291944 | HMGA2 | intron | middle of large intron |
| 6 | 81291991 | HMGA2 | intron | middle of large intron |
| 6 | 81291992 | HMGA2 | intron | middle of large intron |
| 6 | 81294018 | HMGA2 | intron | middle of large intron |
| 6 | 81294149 | HMGA2 | intron | middle of large intron |
| 6 | 81295806 | HMGA2 | intron | middle of large intron |
| 6 | 81299222 | HMGA2 | intron | middle of large intron |
| 6 | 81299233 | HMGA2 | intron | middle of large intron |
| 6 | 81299480 | HMGA2 | intron | middle of large intron |
| 6 | 81302238 | HMGA2 | intron | middle of large intron |
| 6 | 81302326 | HMGA2 | intron | middle of large intron |
| 6 | 81303605 | HMGA2 | intron | middle of large intron |
| 6 | 81306187 | HMGA2 | intron | middle of large intron |
| 6 | 81515618 |  | intergenic |  |
| 6 | 81526654 |  | intergenic |  |
| 6 | 81533130 |  | intergenic |  |
| 6 | 81533415 |  | intergenic |  |
| 6 | 81541746 |  | intergenic |  |
| 6 | 81551859 |  | intergenic |  |
| 6 | 81554686 |  | intergenic |  |
| 6 | 81556148 |  | intergenic |  |
| 6 | 81562316 |  | intergenic |  |
| 6 | 81565125 |  | intergenic |  |
| 6 | 81566097 |  | intergenic |  |
| 6 | 81566780 |  | intergenic |  |
| 6 | 81567455 |  | intergenic |  |
| 6 | 81583251 |  | intergenic |  |
| 6 | 81616193 |  | intergenic |  |
| 6 | 81628792 |  | intergenic |  |
| 6 | 81636830 |  | intergenic |  |
| 6 | 81651604 | LLPH | intron | middle of 5kb intron |
| 6 | 81668230 | ENSECAG00000006721 | intron | 10bp from jxn |
| 6 | 81672724 | ENSECAG00000006721 | intron | 42bp from jxn |
| 6 | 81693743 |  | intergenic |  |
| 6 | 81698889 |  | intergenic |  |
| 9 | 29138245 |  | intergenic |  |
| 9 | 29141598 |  | intergenic |  |
| 9 | 29141611 |  | intergenic |  |
| 9 | 29146150 |  | intergenic |  |
| 9 | 29146151 |  | intergenic |  |
| 9 | 29150200 |  | intergenic |  |
| 9 | 29150826 |  | intergenic |  |
| 9 | 29155084 |  | intergenic |  |
| 9 | 29155106 |  | intergenic |  |
| 9 | 29164356 |  | intergenic |  |
| 9 | 29168790 |  | intergenic |  |
| 9 | 29168833 |  | intergenic |  |
| 9 | 29175626 |  | intergenic |  |
| 9 | 29183183 |  | intergenic |  |
| 9 | 29189764 | MRPL15 | downstream | ~10kb downstream |
| 9 | 29210827 | MRPL15 | intron | ~2kb into a 126+kb intron |
| 9 | 29211591 | MRPL15 | intron | ~2kb into a 126+kb intron |
| 9 | 44603446 | STK3 | intron | 57+kb intron |
| 9 | 44625404 | STK3 | intron | 500bp from jxn |
| 9 | 44702634 | STK3 | intron | 74+kb intron |
| 11 | 29529313 |  | intergenic |  |
| 11 | 29530232 |  | intergenic |  |
| 11 | 29530279 |  | intergenic |  |
| 11 | 29532466 |  | intergenic |  |
| 11 | 29534034 |  | intergenic |  |
| 11 | 29534065 |  | intergenic |  |
| 11 | 29534525 |  | intergenic |  |
| 11 | 29534884 |  | intergenic |  |
| 11 | 29535132 |  | intergenic |  |
| 11 | 29535956 |  | intergenic |  |
| 11 | 29536673 |  | intergenic |  |
| 11 | 29536765 |  | intergenic |  |
| 11 | 29536855 |  | intergenic |  |
| 11 | 29536891 |  | intergenic |  |
| 11 | 29538149 |  | intergenic |  |
| 11 | 29539902 |  | intergenic |  |
| 11 | 29539963 |  | intergenic |  |
| 11 | 29541709 |  | intergenic |  |
| 11 | 29541854 |  | intergenic |  |
| 11 | 29547329 |  | intergenic |  |
| 11 | 29549013 |  | intergenic |  |
| 11 | 29550642 |  | intergenic |  |
| 11 | 29551686 |  | intergenic |  |
| 11 | 29552157 |  | intergenic |  |
| 11 | 29552913 |  | intergenic |  |
| 11 | 29559192 |  | intergenic |  |
| 11 | 29559211 |  | intergenic |  |
| 11 | 29559276 |  | intergenic |  |
| 11 | 29559459 |  | intergenic |  |
| 11 | 29559553 |  | intergenic |  |
| 11 | 29564206 |  | intergenic |  |
| 11 | 29571044 |  | intergenic |  |
| 11 | 29571518 |  | intergenic |  |
| 11 | 29577046 |  | intergenic |  |
| 11 | 29577607 |  | intergenic |  |
| 11 | 29583829 |  | intergenic |  |
| 11 | 29586222 |  | intergenic |  |
| 11 | 29589172 |  | intergenic |  |
| 11 | 29590250 |  | intergenic |  |
| 11 | 29590823 |  | intergenic |  |
| 11 | 29591125 |  | intergenic |  |
| 11 | 29591231 |  | intergenic |  |
| 11 | 29591236 |  | intergenic |  |
| 11 | 29592760 |  | intergenic |  |
| 11 | 29595348 |  | intergenic |  |
| 11 | 29598331 |  | intergenic |  |
| 11 | 29599837 |  | intergenic |  |
| 11 | 31303355 | TRIM25 | downstream | ~5kb downstream |
| 11 | 31305385 | TRIM25 | downstream | ~3kb downstream |
| 11 | 31319004 | TRIM25 | intron | 450bp from jxn |
| 11 | 31353485 |  | intergenic |  |
| 11 | 31414482 |  | intergenic |  |
| 11 | 31470618 |  | intergenic |  |
| 11 | 36608682 | TADA2A | intron | middle of 9kb intron |
| 11 | 36653512 |  | intergenic |  |
| 11 | 36667395 | SYNRG | intron | 150bp from jxn |
| 11 | 36669528 | SYNRG | intron | 50bp from jxn |
| 11 | 36670834 | SYNRG | intron | middle of 14kb intron |
| 11 | 36677118 | SYNRG | intron | middle of 14kb intron |
| 11 | 36686818 | SYNRG | intron | ~250bp from jxn |
| 11 | 36687658 | SYNRG | intron | 500bp from jxn |
| 11 | 36688936 | SYNRG | intron | 40bp from jxn |
| 11 | 36710954 | SYNRG | intron | 200bp from jxn |
| 11 | 36714209 | SYNRG | intron | 400bp from jxn |
| 11 | 36714823 | SYNRG | intron | 73bp from jxn |
| 11 | 36721118 | SYNRG | intron | 21+kb intron |
| 11 | 36722416 | SYNRG | intron | 21+kb intron |
| 11 | 36728557 | SYNRG | intron | 21+kb intron |
| 11 | 36747584 | SYNRG | intron | 5kb intron |
| 11 | 36770599 | DDX52 | intron | 4.5kb intron |
| 11 | 36775488 | DDX52 | upstream | 500kb upstream |
| 11 | 36775489 | DDX52 | upstream | 500kb upstream |
| 11 | 36786229 |  | intergenic |  |
| 11 | 36788199 |  | intergenic |  |
| 11 | 36791629 |  | intergenic |  |
| 11 | 36792412 |  | intergenic |  |
| 11 | 36792455 |  | intergenic |  |
| 11 | 36792590 |  | intergenic |  |
| 11 | 36792649 |  | intergenic |  |
| 11 | 36792750 |  | intergenic |  |
| 11 | 36792751 |  | intergenic |  |
| 11 | 36792787 |  | intergenic |  |
| 11 | 36793429 |  | intergenic |  |
| 11 | 36793434 |  | intergenic |  |
| 11 | 36793443 |  | intergenic |  |
| 11 | 36794678 |  | intergenic |  |
| 11 | 36794771 |  | intergenic |  |
| 11 | 36796850 |  | intergenic |  |
| 11 | 36796891 |  | intergenic |  |
| 12 | 16254700 | ENSECAG00000013095 | upstream | ~7kb upstream |
| 12 | 16262259 | ENSECAG00000013095 | intron | ~1kb from jxn |
| 12 | 16262260 | ENSECAG00000013095 | intron | ~1kb from jxn |
| 12 | 16270318 | ENSECAG00000013095 | intron | ~150bp from jxn |
| 12 | 16275176 | ENSECAG00000013095 | intron | ~3kb from jxn |
| 12 | 16284405 | ENSECAG00000013095 | intron | ~100bp from jxn |
| 12 | 16380392 | ENSECAG00000013249 | upstream | ~5kb upstream |
| 12 | 16404844 |  | intergenic |  |
| 12 | 16407155 |  | intergenic |  |
| 12 | 16408579 |  | intergenic |  |
| 12 | 16409486 |  | intergenic |  |
| 12 | 16410696 |  | intergenic |  |
| 12 | 16410926 |  | intergenic |  |
| 12 | 16411655 |  | intergenic |  |
| 12 | 16411692 |  | intergenic |  |
| 12 | 16412024 |  | intergenic |  |
| 12 | 16412280 |  | intergenic |  |
| 12 | 16412374 |  | intergenic |  |
| 12 | 16419205 |  | intergenic |  |
| 12 | 16420600 |  | intergenic |  |
| 12 | 16420867 |  | intergenic |  |
| 12 | 16420877 |  | intergenic |  |
| 12 | 16421318 |  | intergenic |  |
| 14 | 1354626 | ENSECAG00000002862 | upstream | ~14kb upstream |
| 14 | 1356082 | ENSECAG00000002862 | upstream | ~12kb upstream |
| 14 | 1357335 | ENSECAG00000002862 | upstream | ~11kb upstream |
| 14 | 1357744 | ENSECAG00000002862 | upstream | ~11kb upstream |
| 14 | 1359270 | ENSECAG00000002862 | upstream | ~9kb upstream |
| 14 | 1363873 | ENSECAG00000002862 | upstream | ~5kb upstream |
| 14 | 1364344 | ENSECAG00000002862 | upstream | ~4kb upstream |
| 14 | 1364574 | ENSECAG00000002862 | upstream | ~4kb upstream |
| 14 | 1364794 | ENSECAG00000002862 | upstream | ~4kb upstream |
| 14 | 1365404 | ENSECAG00000002862 | upstream | ~3kb upstream |
| 14 | 1365670 | ENSECAG00000002862 | upstream | ~3kb upstream |
| 14 | 1365947 | ENSECAG00000002862 | upstream | ~3kb upstream |
| 14 | 1366107 | ENSECAG00000002862 | upstream | ~2kb upstream |
| 14 | 1366410 | ENSECAG00000002862 | upstream | ~2kb upstream |
| 14 | 1367543 | ENSECAG00000002862 | upstream | ~700bp upstream |
| 14 | 1368061 | ENSECAG00000002862 | upstream | 200bp upstream |
| 14 | 1368081 | ENSECAG00000002862 | upstream | 200bp upstream |
| 14 | 1369366 | ENSECAG00000002862 | downstream | 120bp downstream |
| 14 | 1369406 | ENSECAG00000002862 | downstream | 200bp downstream |
| 14 | 1369409 | ENSECAG00000002862 | downstream | 200bp downstream |
| 14 | 1369612 | ENSECAG00000002862 | downstream | 400bp downstream |
| 14 | 1369641 | ENSECAG00000002862 | downstream | 400bp downstream |
| 14 | 1369689 | ENSECAG00000002862 | downstream | 400bp downstream |
| 14 | 1369705 | ENSECAG00000002862 | downstream | 500bp downstream |
| 14 | 1370996 | ENSECAG00000002862 | downstream | ~1kb downstream |
| 14 | 1371249 | ENSECAG00000002862 | downstream | ~2kb downstream |
| 14 | 1372129 | ENSECAG00000002862 | downstream | ~3 kb downstream |
| 14 | 1372338 | ENSECAG00000002862 | downstream | ~3 kb downstream |
| 14 | 1372522 | ENSECAG00000002862 | downstream | ~3 kb downstream |
| 14 | 1372972 | ENSECAG00000002862 | downstream | ~3 kb downstream |
| 14 | 1378249 | ENSECAG00000002862 | downstream | ~9kb downstream |
| 14 | 1378304 | ENSECAG00000002862 | downstream | ~9kb downstream |
| 14 | 1378419 | ENSECAG00000002862 | downstream | ~9kb downstream |
| 14 | 1380058 | ENSECAG00000003177 | upstream | ~8kb upstream |
| 14 | 1380260 | ENSECAG00000003177 | upstream | ~8kb upstream |
| 14 | 1380637 | ENSECAG00000003177 | upstream | ~8kb upstream |
| 14 | 1380658 | ENSECAG00000003177 | upstream | ~8kb upstream |
| 14 | 1380836 | ENSECAG00000003177 | upstream | ~8kb upstream |
| 14 | 1381144 | ENSECAG00000003177 | upstream | ~7kb upstream |
| 14 | 1381807 | ENSECAG00000003177 | upstream | ~7kb upstream |
| 14 | 1382350 | ENSECAG00000003177 | upstream | ~6kb upstream |
| 14 | 1382361 | ENSECAG00000003177 | upstream | ~6kb upstream |
| 14 | 1382915 | ENSECAG00000003177 | upstream | ~6kb upstream |
| 14 | 1383866 | ENSECAG00000003177 | upstream | ~5kb upstream |
| 14 | 1384679 | ENSECAG00000003177 | upstream | ~4kb upstream |
| 14 | 1384723 | ENSECAG00000003177 | upstream | ~4kb upstream |
| 14 | 1384752 | ENSECAG00000003177 | upstream | ~4kb upstream |
| 14 | 1384822 | ENSECAG00000003177 | upstream | ~4kb upstream |
| 14 | 1385733 | ENSECAG00000003177 | upstream | ~3kb upstream |
| 14 | 1386060 | ENSECAG00000003177 | upstream | ~2kb upstream |
| 14 | 1386145 | ENSECAG00000003177 | upstream | ~2kb upstream |
| 14 | 1386449 | ENSECAG00000003177 | upstream | ~2kb upstream |
| 14 | 1386702 | ENSECAG00000003177 | upstream | ~2kb upstream |
| 14 | 1386737 | ENSECAG00000003177 | upstream | ~2kb upstream |
| 14 | 1387335 | ENSECAG00000003177 | upstream | ~1kb upstream |
| 14 | 1387425 | ENSECAG00000003177 | upstream | ~1kb upstream |
| 14 | 1387465 | ENSECAG00000003177 | upstream | ~1kb upstream |
| 14 | 1388861 | ENSECAG00000003177 | exon | single exon |
| 14 | 1388939 | ENSECAG00000003177 | exon | single exon |
| 14 | 1389681 | ENSECAG00000003177 | downstream | 350bp downstream |
| 14 | 1389713 | ENSECAG00000003177 | downstream | 350bp downstream |
| 14 | 1390727 | ENSECAG00000003177 | downstream | ~1kb downstream |
| 14 | 1390926 | ENSECAG00000003177 | downstream | ~1kb downstream |
| 14 | 1391074 | ENSECAG00000003177 | downstream | ~2kb downstream |
| 14 | 1391594 | ENSECAG00000003177 | downstream | ~2kb downstream |
| 14 | 1392437 | ENSECAG00000003177 | downstream | ~3kb downstream |
| 14 | 1392552 | ENSECAG00000003177 | downstream | ~3kb downstream |
| 14 | 1392569 | ENSECAG00000003177 | downstream | ~3kb downstream |
| 14 | 1392575 | ENSECAG00000003177 | downstream | ~3kb downstream |
| 14 | 1392694 | ENSECAG00000003177 | downstream | ~3kb downstream |
| 14 | 1392998 | ENSECAG00000003177 | downstream | ~3kb downstream |
| 14 | 1393017 | ENSECAG00000003177 | downstream | ~4kb downstream |
| 14 | 1393169 | ENSECAG00000003177 | downstream | ~4kb downstream |
| 14 | 1393479 | ENSECAG00000003177 | downstream | ~4kb downstream |
| 14 | 1393484 | ENSECAG00000003177 | downstream | ~4kb downstream |
| 14 | 1393763 | ENSECAG00000003177 | downstream | ~4kb downstream |
| 14 | 1394023 | ENSECAG00000003177 | downstream | ~5kb downstream |
| 14 | 1394208 | ENSECAG00000003177 | downstream | ~5kb downstream |
| 14 | 1394296 | ENSECAG00000003177 | downstream | ~5kb downstream |
| 14 | 1394448 | ENSECAG00000003177 | downstream | ~5kb downstream |
| 14 | 1394586 | ENSECAG00000003177 | downstream | ~5kb downstream |
| 14 | 1394765 | ENSECAG00000003177 | downstream | ~5kb downstream |
| 14 | 1394911 | ENSECAG00000003177 | downstream | ~5kb downstream |
| 14 | 1395105 | ENSECAG00000011328 (pseudogene) | upstream | ~5kb upstream |
| 14 | 1395130 | ENSECAG00000011328 (pseudogene) | upstream | ~5kb upstream |
| 14 | 1395131 | ENSECAG00000011328 (pseudogene) | upstream | ~5kb upstream |
| 14 | 1395159 | ENSECAG00000011328 (pseudogene) | upstream | ~5kb upstream |
| 14 | 1395354 | ENSECAG00000011328 (pseudogene) | upstream | ~5kb upstream |
| 14 | 1395430 | ENSECAG00000011328 (pseudogene) | upstream | ~5kb upstream |
| 14 | 1395561 | ENSECAG00000011328 (pseudogene) | upstream | ~5kb upstream |
| 14 | 1395581 | ENSECAG00000011328 (pseudogene) | upstream | ~5kb upstream |
| 14 | 1395591 | ENSECAG00000011328 (pseudogene) | upstream | ~5kb upstream |
| 14 | 1395612 | ENSECAG00000011328 (pseudogene) | upstream | ~5kb upstream |
| 14 | 1395613 | ENSECAG00000011328 (pseudogene) | upstream | ~5kb upstream |
| 14 | 1395834 | ENSECAG00000011328 (pseudogene) | upstream | ~5kb upstream |
| 14 | 1395878 | ENSECAG00000011328 (pseudogene) | upstream | ~5kb upstream |
| 14 | 1395925 | ENSECAG00000011328 (pseudogene) | upstream | ~5kb upstream |
| 14 | 1396517 | ENSECAG00000011328 (pseudogene) | upstream | ~4kb upstream |
| 14 | 1397480 | ENSECAG00000011328 (pseudogene) | upstream | ~3kb upstream |
| 14 | 1398575 | ENSECAG00000011328 (pseudogene) | upstream | ~2kb upstream |
| 14 | 1399069 | ENSECAG00000011328 (pseudogene) | upstream | ~1kb upstream |
| 14 | 1399660 | ENSECAG00000011328 (pseudogene) | upstream | ~1kb upstream |
| 14 | 1399894 | ENSECAG00000011328 (pseudogene) | upstream | ~1kb upstream |
| 14 | 1402723 | ENSECAG00000011328 (pseudogene) | downstream | ~2kb downstream |
| 14 | 1403046 | ENSECAG00000011328 (pseudogene) | downstream | ~3kb downstream |
| 14 | 1403073 | ENSECAG00000011328 (pseudogene) | downstream | ~3kb downstream |
| 14 | 1403134 | ENSECAG00000011328 (pseudogene) | downstream | ~3kb downstream |
| 14 | 1403161 | ENSECAG00000011328 (pseudogene) | downstream | ~3kb downstream |
| 14 | 1403341 | ENSECAG00000011328 (pseudogene) | downstream | ~3kb downstream |
| 14 | 1403357 | ENSECAG00000011328 (pseudogene) | downstream | ~3kb downstream |
| 14 | 1403420 | ENSECAG00000011328 (pseudogene) | downstream | ~3kb downstream |
| 14 | 1403627 | ENSECAG00000011328 (pseudogene) | downstream | ~3kb downstream |
| 14 | 1403859 | ENSECAG00000011328 (pseudogene) | downstream | ~3kb downstream |
| 14 | 1403901 | ENSECAG00000011328 (pseudogene) | downstream | ~3kb downstream |
| 14 | 1404116 | ENSECAG00000011328 (pseudogene) | downstream | ~4kb downstream |
| 14 | 1404472 | ENSECAG00000011328 (pseudogene) | downstream | ~4kb downstream |
| 14 | 1404546 | ENSECAG00000011328 (pseudogene) | downstream | ~4kb downstream |
| 14 | 1404604 | ENSECAG00000011328 (pseudogene) | downstream | ~4kb downstream |
| 14 | 1404605 | ENSECAG00000011328 (pseudogene) | downstream | ~4kb downstream |
| 14 | 1404675 | ENSECAG00000011328 (pseudogene) | downstream | ~4kb downstream |
| 14 | 1404769 | ENSECAG00000011328 (pseudogene) | downstream | ~4kb downstream |
| 14 | 1404805 | ENSECAG00000011328 (pseudogene) | downstream | ~4kb downstream |
| 14 | 1406063 |  | intergenic |  |
| 14 | 1406064 |  | intergenic |  |
| 14 | 1406088 |  | intergenic |  |
| 14 | 1406571 |  | intergenic |  |
| 14 | 1406860 |  | intergenic |  |
| 14 | 1406870 |  | intergenic |  |
| 14 | 1406888 |  | intergenic |  |
| 14 | 1406937 |  | intergenic |  |
| 14 | 1407808 |  | intergenic |  |
| 14 | 1407938 |  | intergenic |  |
| 14 | 1408353 |  | intergenic |  |
| 14 | 1408574 |  | intergenic |  |
| 14 | 1409018 |  | intergenic |  |
| 14 | 1409094 |  | intergenic |  |
| 14 | 1409127 |  | intergenic |  |
| 14 | 1409143 |  | intergenic |  |
| 14 | 1409292 |  | intergenic |  |
| 14 | 1409724 |  | intergenic |  |
| 14 | 1409962 |  | intergenic |  |
| 14 | 1410021 |  | intergenic |  |
| 14 | 1410060 |  | intergenic |  |
| 14 | 1410445 |  | intergenic |  |
| 14 | 1410877 |  | intergenic |  |
| 14 | 1410889 |  | intergenic |  |
| 14 | 1410990 |  | intergenic |  |
| 14 | 1411434 |  | intergenic |  |
| 14 | 1411463 |  | intergenic |  |
| 14 | 1411719 |  | intergenic |  |
| 14 | 1411905 |  | intergenic |  |
| 14 | 1411958 |  | intergenic |  |
| 14 | 1412188 |  | intergenic |  |
| 14 | 1412576 |  | intergenic |  |
| 14 | 1412767 |  | intergenic |  |
| 14 | 1412865 |  | intergenic |  |
| 14 | 1413229 |  | intergenic |  |
| 14 | 1413293 |  | intergenic |  |
| 14 | 1413415 |  | intergenic |  |
| 14 | 1413479 |  | intergenic |  |
| 14 | 1413515 |  | intergenic |  |
| 14 | 1413600 |  | intergenic |  |
| 14 | 1413854 |  | intergenic |  |
| 14 | 1414002 |  | intergenic |  |
| 14 | 1414226 |  | intergenic |  |
| 14 | 1414387 |  | intergenic |  |
| 14 | 1414627 |  | intergenic |  |
| 14 | 1415626 |  | intergenic |  |
| 14 | 1416049 |  | intergenic |  |
| 14 | 1416859 |  | intergenic |  |
| 14 | 1419612 |  | intergenic |  |
| 14 | 1420163 |  | intergenic |  |
| 14 | 1420228 |  | intergenic |  |
| 14 | 1420654 |  | intergenic |  |
| 14 | 1420714 |  | intergenic |  |
| 14 | 1420784 |  | intergenic |  |
| 14 | 1421169 |  | intergenic |  |
| 14 | 1423038 |  | intergenic |  |
| 14 | 1423120 |  | intergenic |  |
| 14 | 1423249 |  | intergenic |  |
| 14 | 1423561 |  | intergenic |  |
| 14 | 1424085 |  | intergenic |  |
| 14 | 1424296 |  | intergenic |  |
| 14 | 1424380 |  | intergenic |  |
| 14 | 1427118 |  | intergenic |  |
| 14 | 1427353 |  | intergenic |  |
| 14 | 1427565 |  | intergenic |  |
| 14 | 1439828 |  | intergenic |  |
| 14 | 1441191 |  | intergenic |  |
| 14 | 1441987 |  | intergenic |  |
| 14 | 1442081 |  | intergenic |  |
| 14 | 1442174 |  | intergenic |  |
| 14 | 1487783 |  | intergenic |  |
| 14 | 1488074 |  | intergenic |  |
| 14 | 1509762 |  | intergenic |  |
| 14 | 1509797 |  | intergenic |  |
| 14 | 1511711 |  | intergenic |  |
| 14 | 1511841 |  | intergenic |  |
| 14 | 1517194 |  | intergenic |  |
| 14 | 1517198 |  | intergenic |  |
| 14 | 1517200 |  | intergenic |  |
| 14 | 1518900 |  | intergenic |  |
| 14 | 1518937 |  | intergenic |  |
| 14 | 1519699 |  | intergenic |  |
| 14 | 1519700 |  | intergenic |  |
| 14 | 1519701 |  | intergenic |  |
| 14 | 1519707 |  | intergenic |  |
| 14 | 1519745 |  | intergenic |  |
| 14 | 1520564 |  | intergenic |  |
| 14 | 1534306 |  | intergenic |  |
| 14 | 1540915 |  | intergenic |  |
| 14 | 1540916 |  | intergenic |  |
| 14 | 1546595 |  | intergenic |  |
| 14 | 1546610 |  | intergenic |  |
| 14 | 1546611 |  | intergenic |  |
| 14 | 1546622 |  | intergenic |  |
| 14 | 1546638 |  | intergenic |  |
| 14 | 1546665 |  | intergenic |  |
| 14 | 1546888 |  | intergenic |  |
| 14 | 1546918 |  | intergenic |  |
| 14 | 1546921 |  | intergenic |  |
| 14 | 1546927 |  | intergenic |  |
| 14 | 1546939 |  | intergenic |  |
| 14 | 1547009 |  | intergenic |  |
| 14 | 1547013 |  | intergenic |  |
| 14 | 1547257 |  | intergenic |  |
| 14 | 1547387 |  | intergenic |  |
| 14 | 1554309 |  | intergenic |  |
| 14 | 1568570 |  | intergenic |  |
| 14 | 1568614 |  | intergenic |  |
| 14 | 1569039 |  | intergenic |  |
| 14 | 1569312 |  | intergenic |  |
| 14 | 1569485 |  | intergenic |  |
| 14 | 1570134 |  | intergenic |  |
| 14 | 1570169 |  | intergenic |  |
| 14 | 1570578 |  | intergenic |  |
| 14 | 1571659 |  | intergenic |  |
| 14 | 1571731 |  | intergenic |  |
| 14 | 1572371 |  | intergenic |  |
| 14 | 1572639 |  | intergenic |  |
| 14 | 1573354 |  | intergenic |  |
| 14 | 5442438 |  | intergenic |  |
| 15 | 10100242 |  | intergenic |  |
| 15 | 10100262 |  | intergenic |  |
| 15 | 10100285 |  | intergenic |  |
| 15 | 10100293 |  | intergenic |  |
| 15 | 10100301 |  | intergenic |  |
| 15 | 10100575 |  | intergenic |  |
| 15 | 10101673 |  | intergenic |  |
| 15 | 10101676 |  | intergenic |  |
| 15 | 10102671 |  | intergenic |  |
| 15 | 10102974 |  | intergenic |  |
| 15 | 10103000 |  | intergenic |  |
| 15 | 10103035 |  | intergenic |  |
| 15 | 10103037 |  | intergenic |  |
| 15 | 10103733 |  | intergenic |  |
| 15 | 10103783 |  | intergenic |  |
| 15 | 10103787 |  | intergenic |  |
| 15 | 10105521 |  | intergenic |  |
| 15 | 10107096 |  | intergenic |  |
| 15 | 10108198 |  | intergenic |  |
| 15 | 10108201 |  | intergenic |  |
| 15 | 10108226 |  | intergenic |  |
| 16 | 59352686 |  | intergenic |  |
| 16 | 59378775 |  | intergenic |  |
| 16 | 59382124 |  | intergenic |  |
| 16 | 59384565 |  | intergenic |  |
| 16 | 59391893 |  | intergenic |  |
| 16 | 59392328 |  | intergenic |  |
| 16 | 59392331 |  | intergenic |  |
| 17 | 50982245 |  | intergenic |  |
| 17 | 50982252 |  | intergenic |  |
| 17 | 50983052 |  | intergenic |  |
| 17 | 50983097 |  | intergenic |  |
| 17 | 50984304 |  | intergenic |  |
| 17 | 50985015 |  | intergenic |  |
| 17 | 50985593 |  | intergenic |  |
| 17 | 50985595 |  | intergenic |  |
| 17 | 50986998 |  | intergenic |  |
| 17 | 50988970 |  | intergenic |  |
| 17 | 50993146 |  | intergenic |  |
| 17 | 51004074 |  | intergenic |  |
| 17 | 51017875 |  | intergenic |  |
| 17 | 51021511 |  | intergenic |  |
| 17 | 51021512 |  | intergenic |  |
| 17 | 51023973 |  | intergenic |  |
| 17 | 51030197 |  | intergenic |  |
| 17 | 51034257 |  | intergenic |  |
| 17 | 51037363 |  | intergenic |  |
| 17 | 51038061 |  | intergenic |  |
| 17 | 51044268 |  | intergenic |  |
| 17 | 61715349 |  | intergenic |  |
| 17 | 61716538 |  | intergenic |  |
| 17 | 61716597 |  | intergenic |  |
| 17 | 61717590 |  | intergenic |  |
| 17 | 61717643 |  | intergenic |  |
| 17 | 61717843 |  | intergenic |  |
| 17 | 61718000 |  | intergenic |  |
| 17 | 61720143 |  | intergenic |  |
| 17 | 61721545 |  | intergenic |  |
| 17 | 61721785 |  | intergenic |  |
| 17 | 61723469 |  | intergenic |  |
| 17 | 61723809 |  | intergenic |  |
| 17 | 61725157 |  | intergenic |  |
| 17 | 61725200 |  | intergenic |  |
| 17 | 61725344 |  | intergenic |  |
| 17 | 61725897 |  | intergenic |  |
| 17 | 61727157 |  | intergenic |  |
| 17 | 61727633 |  | intergenic |  |
| 17 | 61728019 |  | intergenic |  |
| 17 | 61728223 |  | intergenic |  |
| 17 | 61744016 |  | intergenic |  |
| 17 | 61749334 |  | intergenic |  |
| 17 | 65640738 | UGGT2 | intron | 12kb intron |
| 17 | 65641810 | UGGT2 | intron | 12kb intron |
| 17 | 65643305 | UGGT2 | intron | 12kb intron |
| 17 | 65643362 | UGGT2 | intron | 12kb intron |
| 17 | 65644154 | UGGT2 | intron | 12kb intron |
| 17 | 65645147 | UGGT2 | intron | 12kb intron |
| 17 | 65646106 | UGGT2 | intron | 12kb intron |
| 17 | 65649022 | UGGT2 | intron | 12kb intron |
| 17 | 65649952 | UGGT2 | intron | 12kb intron |
| 17 | 65656166 | UGGT2 | intron | 9kb intron |
| 17 | 65656483 | UGGT2 | intron | 9kb intron |
| 17 | 65659030 | UGGT2 | intron | 9kb intron |
| 17 | 65659694 | UGGT2 | intron | 9kb intron |
| 17 | 65661479 | UGGT2 | intron | 9kb intron |
| 20 | 25103297 |  | intergenic |  |
| 20 | 25103556 |  | intergenic |  |
| 20 | 25103570 |  | intergenic |  |
| 20 | 25113918 |  | intergenic |  |
| 20 | 25127274 |  | intergenic |  |
| 20 | 27650699 | ENSECAG00000004173 | downstream | ~5kb downstream |
| 20 | 27677564 |  | intergenic |  |
| 20 | 27678118 |  | intergenic |  |
| 20 | 27678421 |  | intergenic |  |
| 20 | 27678432 |  | intergenic |  |
| 20 | 27679522 |  | intergenic |  |
| 20 | 27679621 |  | intergenic |  |
| 20 | 27679622 |  | intergenic |  |
| 20 | 27680819 |  | intergenic |  |
| 20 | 27681133 |  | intergenic |  |
| 20 | 27682366 |  | intergenic |  |
| 20 | 27682374 |  | intergenic |  |
| 20 | 27682680 |  | intergenic |  |
| 20 | 27682699 |  | intergenic |  |
| 20 | 27683200 |  | intergenic |  |
| 20 | 27683465 |  | intergenic |  |
| 20 | 27683777 |  | intergenic |  |
| 20 | 27684873 |  | intergenic |  |
| 20 | 27685050 |  | intergenic |  |
| 20 | 27685448 |  | intergenic |  |
| 20 | 27686620 |  | intergenic |  |
| 20 | 27686713 |  | intergenic |  |
| 20 | 27686733 |  | intergenic |  |
| 20 | 27687540 |  | intergenic |  |
| 20 | 27687571 |  | intergenic |  |
| 20 | 27688883 | ENSECAG00000004281 | downstream | ~2kb downstream |
| 20 | 27689576 | ENSECAG00000004281 | downstream | ~1kb downstream |
| 20 | 27691110 | ENSECAG00000004281 | exon | exon 1 |
| 20 | 27692730 | ENSECAG00000004281 | upstream | ~1kb upstream |
| 20 | 27692740 | ENSECAG00000004281 | upstream | ~1kb upstream |
| 20 | 27692751 | ENSECAG00000004281 | upstream | ~1kb upstream |
| 20 | 27692861 | ENSECAG00000004281 | upstream | ~1kb upstream |
| 20 | 27692869 | ENSECAG00000004281 | upstream | ~1kb upstream |
| 20 | 27694234 | ENSECAG00000004281 | upstream | ~3kb upstream |
| 20 | 27694252 | ENSECAG00000004281 | upstream | ~3kb upstream |
| 20 | 27694321 | ENSECAG00000004281 | upstream | ~3kb upstream |
| 20 | 27695249 | ENSECAG00000004281 | upstream | ~4kb upstream |
| 20 | 27695624 | ENSECAG00000004281 | upstream | ~4kb upstream |
| 20 | 27696803 | ENSECAG00000004281 | upstream | ~5kb upstream |
| 20 | 27696843 | ENSECAG00000004281 | upstream | ~5kb upstream |
| 20 | 27696849 | ENSECAG00000004281 | upstream | ~5kb upstream |
| 20 | 27696887 | ENSECAG00000004281 | upstream | ~5kb upstream |
| 20 | 27696929 | ENSECAG00000004281 | upstream | ~5kb upstream |
| 20 | 27697193 |  | intergenic |  |
| 20 | 27698202 |  | intergenic |  |
| 20 | 27701333 |  | intergenic |  |
| 20 | 27705270 |  | intergenic |  |
| 20 | 27707260 |  | intergenic |  |
| 20 | 27707961 |  | intergenic |  |
| 20 | 27710614 | ENSECAG00000004382 | downstream | 280bp downstream |
| 20 | 27711111 | ENSECAG00000004382 | exon | single exon |
| 20 | 27711142 | ENSECAG00000004382 | exon | single exon |
| 20 | 27711170 | ENSECAG00000004382 | exon | single exon |
| 20 | 27711193 | ENSECAG00000004382 | exon | single exon |
| 20 | 27712561 | ENSECAG00000004382 | upstream | ~700bp upstream |
| 20 | 27712604 | ENSECAG00000004382 | upstream | ~800bp upstream |
| 20 | 27713230 | ENSECAG00000004382 | upstream | ~2kb upstream |
| 20 | 27714181 |  | intergenic |  |
| 20 | 27714361 |  | intergenic |  |
| 20 | 27716125 |  | intergenic |  |
| 20 | 27716177 |  | intergenic |  |
| 20 | 27716556 |  | intergenic |  |
| 20 | 27716561 |  | intergenic |  |
| 20 | 27716583 |  | intergenic |  |
| 20 | 27718205 |  | intergenic |  |
| 20 | 27718244 |  | intergenic |  |
| 20 | 27718447 |  | intergenic |  |
| 20 | 27718507 |  | intergenic |  |
| 20 | 27718526 |  | intergenic |  |
| 20 | 27718537 |  | intergenic |  |
| 20 | 27718558 |  | intergenic |  |
| 20 | 27718867 |  | intergenic |  |
| 20 | 27718875 |  | intergenic |  |
| 20 | 27718899 |  | intergenic |  |
| 20 | 27718903 |  | intergenic |  |
| 20 | 27718918 |  | intergenic |  |
| 20 | 27718930 |  | intergenic |  |
| 20 | 27719196 |  | intergenic |  |
| 20 | 27720858 |  | intergenic |  |
| 20 | 27720865 |  | intergenic |  |
| 20 | 27722002 |  | intergenic |  |
| 20 | 27722009 |  | intergenic |  |
| 20 | 27722038 |  | intergenic |  |
| 20 | 27722066 |  | intergenic |  |
| 20 | 27722081 |  | intergenic |  |
| 20 | 27722616 |  | intergenic |  |
| 20 | 27722625 |  | intergenic |  |
| 20 | 27722945 |  | intergenic |  |
| 20 | 27723194 |  | intergenic |  |
| 20 | 27724444 | ENSECAG00000004474 | downstream | ~2kb downstream |
| 20 | 27724452 | ENSECAG00000004474 | downstream | ~2kb downstream |
| 20 | 27724468 | ENSECAG00000004474 | downstream | ~2kb downstream |
| 20 | 27724722 | ENSECAG00000004474 | downstream | ~2kb downstream |
| 20 | 27725322 | ENSECAG00000004474 | downstream | ~1kb downstream |
| 20 | 27725405 | ENSECAG00000004474 | downstream | ~1kb downstream |
| 20 | 27725412 | ENSECAG00000004474 | downstream | ~1kb downstream |
| 20 | 27725440 | ENSECAG00000004474 | downstream | ~1kb downstream |
| 20 | 27725643 | ENSECAG00000004474 | downstream | ~1kb downstream |
| 20 | 27725702 | ENSECAG00000004474 | downstream | ~1kb downstream |
| 20 | 27727102 | ENSECAG00000004474 | exon | single exon |
| 20 | 27727105 | ENSECAG00000004474 | exon | single exon |
| 20 | 27728083 | ENSECAG00000004474 | upstream | ~1kb upstream |
| 20 | 27728456 | ENSECAG00000004474 | upstream | ~1kb upstream |
| 20 | 27728546 | ENSECAG00000004474 | upstream | ~1kb upstream |
| 20 | 27728568 | ENSECAG00000004474 | upstream | ~1kb upstream |
| 20 | 27728678 | ENSECAG00000004474 | upstream | ~1kb upstream |
| 20 | 27728814 | ENSECAG00000004474 | upstream | ~1kb upstream |
| 20 | 27728959 | ENSECAG00000004474 | upstream | ~1kb upstream |
| 20 | 27729198 | ENSECAG00000004474 | upstream | ~2kb upstream |
| 20 | 27729248 | ENSECAG00000004474 | upstream | ~2kb upstream |
| 20 | 27730687 |  | intergenic |  |
| 20 | 27731494 |  | intergenic |  |
| 20 | 27732252 |  | intergenic |  |
| 20 | 27733466 |  | intergenic |  |
| 20 | 27733474 |  | intergenic |  |
| 20 | 27734364 |  | intergenic |  |
| 20 | 27734395 |  | intergenic |  |
| 20 | 27750246 |  | intergenic |  |
| 20 | 27750247 |  | intergenic |  |
| 20 | 27753344 |  | intergenic |  |
| 20 | 27753377 |  | intergenic |  |
| 20 | 27755613 |  | intergenic |  |
| 20 | 27755618 |  | intergenic |  |
| 20 | 27755669 |  | intergenic |  |
| 20 | 27756799 |  | intergenic |  |
| 20 | 27765139 | ENSECAG00000004766 | downstream | ~2kb downstream |
| 20 | 27766454 | ENSECAG00000004766 | downstream | ~1kb downstream |
| 20 | 27766622 | ENSECAG00000004766 | downstream | ~1kb downstream |
| 20 | 27766626 | ENSECAG00000004766 | downstream | ~1kb downstream |
| 20 | 27768899 | ENSECAG00000004766 | exon | single exon |
| 20 | 27768928 | ENSECAG00000004766 | upstream | 22bp upstream |
| 20 | 27772292 | ENSECAG00000004766 | upstream | ~4kb upstream |
| 20 | 27779466 |  | intergenic |  |
| 20 | 27779467 |  | intergenic |  |
| 20 | 27779678 |  | intergenic |  |
| 20 | 27779679 |  | intergenic |  |
| 20 | 46929785 |  | intergenic |  |
| 20 | 46938119 |  | intergenic |  |
| 20 | 46938194 |  | intergenic |  |
| 20 | 46940840 |  | intergenic |  |
| 20 | 46948920 |  | intergenic |  |
| 20 | 46954407 |  | intergenic |  |
| 20 | 46959352 |  | intergenic |  |
| 20 | 46961424 |  | intergenic |  |
| 20 | 46963301 |  | intergenic |  |
| 20 | 47055413 |  | intergenic |  |
| 20 | 47057963 |  | intergenic |  |
| 20 | 47062579 |  | intergenic |  |
| 20 | 47072002 |  | intergenic |  |
| 20 | 47072639 |  | intergenic |  |
| 20 | 47076296 |  | intergenic |  |
| 20 | 47076312 |  | intergenic |  |
| 20 | 47079207 |  | intergenic |  |
| 20 | 47081583 |  | intergenic |  |
| 20 | 47082505 |  | intergenic |  |
| 20 | 47083944 |  | intergenic |  |
| 20 | 47084164 |  | intergenic |  |
| 20 | 47085775 |  | intergenic |  |
| 20 | 47086109 |  | intergenic |  |
| 20 | 47086123 |  | intergenic |  |
| 20 | 47086342 |  | intergenic |  |
| 20 | 47086358 |  | intergenic |  |
| 20 | 47086683 |  | intergenic |  |
| 20 | 47086747 |  | intergenic |  |
| 20 | 47087205 |  | intergenic |  |
| 20 | 47087589 |  | intergenic |  |
| 20 | 47088259 |  | intergenic |  |
| 20 | 47088816 |  | intergenic |  |
| 20 | 47089630 |  | intergenic |  |
| 20 | 47089928 |  | intergenic |  |
| 20 | 47090966 |  | intergenic |  |
| 20 | 47091020 |  | intergenic |  |
| 20 | 47091147 |  | intergenic |  |
| 20 | 47091847 |  | intergenic |  |
| 20 | 47091892 |  | intergenic |  |
| 20 | 47091927 |  | intergenic |  |
| 20 | 47092658 |  | intergenic |  |
| 23 | 14636314 | ENSECAG00000010004 | upstream | ~10kb upstream |
| 23 | 14636912 | ENSECAG00000010004 | upstream | ~10kb upstream |
| 23 | 14637525 | ENSECAG00000010004 | upstream | ~9kb upstream |
| 23 | 14638362 | ENSECAG00000010004 | upstream | ~8kb upstream |
| 23 | 14639375 | ENSECAG00000010004 | upstream | ~7kb upstream |
| 23 | 14639424 | ENSECAG00000010004 | upstream | ~7kb upstream |
| 23 | 14639877 | ENSECAG00000010004 | upstream | ~7kb upstream |
| 23 | 14639969 | ENSECAG00000010004 | upstream | ~7kb upstream |
| 23 | 14640048 | ENSECAG00000010004 | upstream | ~6kb upstream |
| 23 | 14640325 | ENSECAG00000010004 | upstream | ~6kb upstream |
| 23 | 14640812 | ENSECAG00000010004 | upstream | ~6kb upstream |
| 23 | 14640960 | ENSECAG00000010004 | upstream | ~6kb upstream |
| 23 | 14641003 | ENSECAG00000010004 | upstream | ~5kb upstream |
| 23 | 14641205 | ENSECAG00000010004 | upstream | ~5kb upstream |
| 23 | 14641662 | ENSECAG00000010004 | upstream | ~5kb upstream |
| 23 | 14642089 | ENSECAG00000010004 | upstream | ~4kb upstream |
| 23 | 14643656 | ENSECAG00000010004 | upstream | ~3kb upstream |
| 23 | 14643895 | ENSECAG00000010004 | upstream | ~3kb upstream |
| 23 | 14643915 | ENSECAG00000010004 | upstream | ~3kb upstream |
| 23 | 14645077 | ENSECAG00000010004 | upstream | ~1kb upstream |
| 23 | 14646865 | ENSECAG00000010004 | intron | 48+ kb intron |
| 23 | 14648247 | ENSECAG00000010004 | intron | 48+ kb intron |
| 23 | 14648590 | ENSECAG00000010004 | intron | 48+ kb intron |
| 23 | 14648801 | ENSECAG00000010004 | intron | 48+ kb intron |
| 23 | 14648823 | ENSECAG00000010004 | intron | 48+ kb intron |
| 23 | 14649783 | ENSECAG00000010004 | intron | 48+ kb intron |
| 23 | 14649864 | ENSECAG00000010004 | intron | 48+ kb intron |
| 23 | 14651935 | ENSECAG00000010004 | intron | 48+ kb intron |
| 23 | 14652482 | ENSECAG00000010004 | intron | 48+ kb intron |
| 23 | 14657752 | ENSECAG00000010004 | intron | 48+ kb intron |
| 23 | 20632496 | MAMDC2 | intron | 25+ kb intron |
| 23 | 20632711 | MAMDC2 | intron | 25+ kb intron |
| 23 | 20632722 | MAMDC2 | intron | 25+ kb intron |
| 23 | 20632768 | MAMDC2 | intron | 25+ kb intron |
| 23 | 20633497 | MAMDC2 | intron | 25+ kb intron |
| 23 | 20636966 | MAMDC2 | intron | 25+ kb intron |
| 23 | 20638245 | MAMDC2 | intron | 25+ kb intron |
| 23 | 20638320 | MAMDC2 | intron | 25+ kb intron |
| 23 | 20638572 | MAMDC2 | intron | 25+ kb intron |
| 23 | 20638576 | MAMDC2 | intron | 25+ kb intron |
| 23 | 20638832 | MAMDC2 | intron | ~500bp from jxn |
| 23 | 20639099 | MAMDC2 | intron | 284bp from jxn |
| 23 | 20639151 | MAMDC2 | intron | 132bp from jxn |
| 23 | 20640201 | MAMDC2 | intron | ~800bp from jxn |
| 23 | 20640290 | MAMDC2 | intron | ~800bp from jxn |
| 23 | 20641385 | MAMDC2 | intron | 130bp from jxn |
| 23 | 20641482 | MAMDC2 | intron | 230bp from jxn |
| 23 | 20645087 | MAMDC2 | intron | 17+kb intron |
| 23 | 20645096 | MAMDC2 | intron | 17+kb intron |
| 23 | 20645111 | MAMDC2 | intron | 17+kb intron |
| 23 | 20645997 | MAMDC2 | intron | 17+kb intron |
| 23 | 20647305 | MAMDC2 | intron | 17+kb intron |
| 23 | 20648419 | MAMDC2 | intron | 17+kb intron |
| 23 | 20648699 | MAMDC2 | intron | 17+kb intron |
| 23 | 20649455 | MAMDC2 | intron | 17+kb intron |
| 23 | 20650124 | MAMDC2 | intron | 17+kb intron |
| 23 | 20650567 | MAMDC2 | intron | 17+kb intron |
| 23 | 20650987 | MAMDC2 | intron | 17+kb intron |
| 23 | 20651027 | MAMDC2 | intron | 17+kb intron |
| 23 | 20651689 | MAMDC2 | intron | 17+kb intron |
| 23 | 20651754 | MAMDC2 | intron | 17+kb intron |
| 23 | 20651834 | MAMDC2 | intron | 17+kb intron |
| 23 | 20651994 | MAMDC2 | intron | 17+kb intron |
| 23 | 20652024 | MAMDC2 | intron | 17+kb intron |
| 23 | 20652060 | MAMDC2 | intron | 17+kb intron |
| 23 | 20652127 | MAMDC2 | intron | 17+kb intron |
| 23 | 20652473 | MAMDC2 | intron | 17+kb intron |
| 23 | 20652865 | MAMDC2 | intron | 17+kb intron |
| 23 | 20652902 | MAMDC2 | intron | 17+kb intron |
| 23 | 20653492 | MAMDC2 | intron | 17+kb intron |
| 23 | 20653967 | MAMDC2 | intron | 17+kb intron |
| 23 | 20653968 | MAMDC2 | intron | 17+kb intron |
| 23 | 20654330 | MAMDC2 | intron | 17+kb intron |
| 23 | 20654555 | MAMDC2 | intron | 17+kb intron |
| 23 | 20657023 | MAMDC2 | intron | 17+kb intron |
| 23 | 20657455 | MAMDC2 | intron | 17+kb intron |
| 23 | 20658007 | MAMDC2 | intron | 900bp from jxn |
| 23 | 20658482 | MAMDC2 | intron | 400bp from jxn |
| 23 | 20658637 | MAMDC2 | intron | 250bp from jxn |
| 23 | 20658692 | MAMDC2 | intron | 200bp from jxn |
| 23 | 20658789 | MAMDC2 | intron | 100bp from jxn |
| 23 | 20659360 | MAMDC2 | intron | 175bp from jxn |
| 23 | 20659526 | MAMDC2 | intron | 350bp from jxn |
| 23 | 20661210 | MAMDC2 | intron | ~1kb from jxn |
| 23 | 20661275 | MAMDC2 | intron | ~1kb from jxn |
| 23 | 20662320 | MAMDC2 | intron | 80bp from jxn |
| 23 | 20662429 | MAMDC2 | intron | 170bp from jxn |
| 24 | 6704886 |  | intergenic |  |
| 24 | 6712987 |  | intergenic |  |
| 24 | 6713012 |  | intergenic |  |
| 24 | 6713425 |  | intergenic |  |
| 24 | 6713671 |  | intergenic |  |
| 24 | 6717093 |  | intergenic |  |
| 24 | 6720493 |  | intergenic |  |
| 24 | 6736928 |  | intergenic |  |
| 24 | 10275207 | RHOJ | intron | middle of 2kb intron |
| 24 | 10275208 | RHOJ | intron | middle of 2kb intron |
| 24 | 10275222 | RHOJ | intron | middle of 2kb intron |
| 24 | 10275223 | RHOJ | intron | middle of 2kb intron |
| 24 | 10275786 | RHOJ | intron | middle of 2kb intron |
| 24 | 10275824 | RHOJ | intron | middle of 2kb intron |
| 24 | 10275827 | RHOJ | intron | middle of 2kb intron |
| 24 | 10275867 | RHOJ | intron | middle of 2kb intron |
| 24 | 10276151 | RHOJ | intron | 160kb from jxn |
| 24 | 10277393 | RHOJ | intron | ~1kb from jxn |
| 24 | 10280645 | RHOJ | intron | middle of 7.5kb intron |
| 24 | 10285906 | RHOJ | downstream | ~1kb downstream |
| 24 | 10287279 | RHOJ | downstream | ~3kb downstream |
| 24 | 10287539 | RHOJ | downstream | ~3kb downstream |
| 24 | 10294169 | GPHB5 | downstream | ~3kb downstream |
| 24 | 10294173 | GPHB5 | downstream | ~3kb downstream |
| 24 | 10294177 | GPHB5 | downstream | ~3kb downstream |
| 24 | 10296168 | GPHB5 | downstream | ~1.5kb downstream |
| 24 | 10296357 | GPHB5 | downstream | ~1.5kb downstream |
| 24 | 10296485 | GPHB5 | downstream | ~1.5kb downstream |
| 24 | 10296512 | GPHB5 | downstream | ~1.5kb downstream |
| 24 | 10296618 | GPHB5 | downstream | ~1kb downstream |
| 24 | 10296619 | GPHB5 | downstream | ~1kb downstream |
| 24 | 10296625 | GPHB5 | downstream | ~1kb downstream |
| 24 | 10297281 | GPHB5 | downstream | 500bp downstream |
| 24 | 10297297 | GPHB5 | downstream | 500bp downstream |
| 24 | 10297624 | GPHB5 | downstream | 200bp downstream |
| 24 | 10297627 | GPHB5 | downstream | 200bp downstream |
| 24 | 10298372 | GPHB5 | intron | 400bp from jxn |
| 24 | 10298380 | GPHB5 | intron | 400bp from jxn |
| 24 | 10298381 | GPHB5 | intron | 400bp from jxn |
| 24 | 10298389 | GPHB5 | intron | 400bp from jxn |
| 24 | 10298994 | GPHB5 | intron | 400bp from jxn |
| 24 | 10299287 | GPHB5 | intron | 4kb intron |
| 24 | 10299288 | GPHB5 | intron | 4kb intron |
| 24 | 10299296 | GPHB5 | intron | 4kb intron |
| 24 | 10299566 | GPHB5 | intron | 4kb intron |
| 24 | 10304373 | GPHB5 | upstream | ~2kb upstream |
| 24 | 10305005 | GPHB5 | upstream | ~3kb upstream |
| 24 | 10306201 | GPHB5 | upstream | ~4kb upstream |
| 24 | 10306202 | GPHB5 | upstream | ~4kb upstream |
| 24 | 10306231 | GPHB5 | upstream | ~4kb upstream |
| 24 | 10310632 |  | intergenic |  |
| 24 | 10310656 |  | intergenic |  |
| 25 | 3656586 | ENSECAG00000013976 | downstream | ~2kb downstream |
| 25 | 3657454 | ENSECAG00000013976 | downstream | ~1kb downstream |
| 25 | 3657591 | ENSECAG00000013976 | downstream | ~1kb downstream |
| 25 | 3662197 | ENSECAG00000013976 | intron | ~1kb from jxn |
| 25 | 3664078 | ENSECAG00000013976 | intron | 600bp from jxn |
| 25 | 3665245 | ENSECAG00000013976 | intron | 700bp from jxn |
| 25 | 3665395 | ENSECAG00000013976 | intron | 500bp from jxn |
| 25 | 3665407 | ENSECAG00000013976 | intron | 500bp from jxn |
| 25 | 3665458 | ENSECAG00000013976 | intron | 500bp from jxn |
| 25 | 3666056 | ENSECAG00000013976 | upstream | 56bp upstream |
| 25 | 3666078 | ENSECAG00000013976 | upstream | 78bp upstream |
| 25 | 3666516 | ENSECAG00000013976 | upstream | 500bp upstream |
| 25 | 3666755 | ENSECAG00000013976 | upstream | 750bp upstream |
| 25 | 3667391 | ENSECAG00000013976 | upstream | ~1kb upstream |
| 25 | 3667392 | ENSECAG00000013976 | upstream | ~1kb upstream |
| 25 | 3668864 | ENSECAG00000013976 | upstream | ~2kb upstream |
| 25 | 3669204 | ENSECAG00000013976 | upstream | ~3kb upstream |
| 25 | 3670342 | ENSECAG00000013976 | upstream | ~4kb upstream |
| 25 | 3670856 | ENSECAG00000013976 | upstream | ~4kb upstream |
| 25 | 3670864 | ENSECAG00000013976 | upstream | ~4kb upstream |
| 25 | 3670919 | ENSECAG00000013976 | upstream | ~4kb upstream |
| 25 | 3671227 | ENSECAG00000013976 | upstream | ~4kb upstream |
| 25 | 3672627 | ENSECAG00000013976 | upstream | ~5kb upstream |
| 25 | 3672938 | ENSECAG00000013976 | upstream | ~5kb upstream |
| 25 | 3675945 |  | intergenic |  |
| 25 | 3677007 |  | intergenic |  |
| 25 | 3677151 |  | intergenic |  |
| 25 | 3677795 |  | intergenic |  |
| 25 | 3678043 |  | intergenic |  |
| 25 | 3678044 |  | intergenic |  |
| 25 | 3678057 |  | intergenic |  |
| 25 | 3678076 |  | intergenic |  |
| 25 | 3678180 |  | intergenic |  |
| 25 | 3678455 |  | intergenic |  |
| 25 | 3679291 |  | intergenic |  |
| 25 | 3679417 |  | intergenic |  |
| 25 | 3681031 |  | intergenic |  |
| 25 | 3681891 |  | intergenic |  |
| 25 | 3682154 |  | intergenic |  |
| 25 | 3682188 |  | intergenic |  |
| 25 | 3685513 |  | intergenic |  |
| 25 | 3686042 |  | intergenic |  |
| 25 | 3686344 |  | intergenic |  |
| 25 | 3686488 |  | intergenic |  |
| 25 | 3686768 |  | intergenic |  |
| 25 | 3687971 |  | intergenic |  |
| 25 | 3688159 |  | intergenic |  |
| 25 | 3688495 |  | intergenic |  |
| 25 | 3688521 |  | intergenic |  |
| 25 | 3688764 |  | intergenic |  |
| 25 | 3688769 |  | intergenic |  |
| 25 | 3689834 |  | intergenic |  |
| 25 | 3691629 |  | intergenic |  |
| 25 | 3693346 |  | intergenic |  |
| 25 | 3694284 |  | intergenic |  |
| 25 | 3694890 |  | intergenic |  |
| 25 | 3696996 |  | intergenic |  |
| 25 | 3697154 |  | intergenic |  |
| 25 | 3698627 |  | intergenic |  |
| 25 | 3698722 |  | intergenic |  |
| 25 | 3698987 |  | intergenic |  |
| 25 | 3700326 | ENSECAG00000014333 (pseudogene) | exon |  |
| 25 | 3700463 | ENSECAG00000014333 (pseudogene) | exon |  |
| 25 | 3701968 |  | intergenic |  |
| 25 | 3702521 |  | intergenic |  |
| 25 | 3703111 |  | intergenic |  |
| 25 | 3703430 |  | intergenic |  |
| 25 | 3703801 |  | intergenic |  |
| 25 | 3703814 |  | intergenic |  |
| 25 | 3703833 |  | intergenic |  |
| 25 | 3703926 |  | intergenic |  |
| 25 | 3703976 |  | intergenic |  |
| 25 | 3703988 |  | intergenic |  |
| 25 | 3705108 |  | intergenic |  |
| 25 | 3705708 |  | intergenic |  |
| 25 | 3705964 |  | intergenic |  |
| 25 | 3706050 |  | intergenic |  |
| 25 | 3706062 |  | intergenic |  |
| 25 | 3706737 |  | intergenic |  |
| 25 | 3706965 |  | intergenic |  |
| 25 | 3707045 |  | intergenic |  |
| 25 | 3707050 |  | intergenic |  |
| 25 | 3708852 |  | intergenic |  |
| 25 | 3708859 |  | intergenic |  |
| 25 | 3709850 |  | intergenic |  |
| 25 | 3710266 |  | intergenic |  |
| 25 | 3710359 |  | intergenic |  |
| 25 | 3714425 |  | intergenic |  |
| 25 | 3714828 |  | intergenic |  |
| 25 | 3715844 |  | intergenic |  |
| 25 | 3716359 |  | intergenic |  |
| 25 | 3717569 |  | intergenic |  |
| 25 | 3717571 |  | intergenic |  |
| 25 | 3717673 |  | intergenic |  |
| 25 | 3717694 |  | intergenic |  |
| 25 | 3719040 |  | intergenic |  |
| 25 | 3720856 |  | intergenic |  |
| 25 | 3720895 |  | intergenic |  |
| 25 | 3720930 |  | intergenic |  |
| 25 | 3720944 |  | intergenic |  |
| 25 | 3723413 |  | intergenic |  |
| 25 | 3723583 |  | intergenic |  |
| 25 | 3723805 |  | intergenic |  |
| 25 | 3723862 |  | intergenic |  |
| 25 | 3723924 |  | intergenic |  |
| 25 | 3724120 |  | intergenic |  |
| 25 | 3724522 |  | intergenic |  |
| 25 | 3724550 |  | intergenic |  |
| 25 | 3724835 |  | intergenic |  |
| 25 | 3725023 |  | intergenic |  |
| 25 | 3727605 |  | intergenic |  |
| 25 | 3727635 |  | intergenic |  |
| 25 | 3728759 |  | intergenic |  |
| 25 | 3728761 |  | intergenic |  |
| 25 | 3729332 |  | intergenic |  |
| 25 | 3729672 |  | intergenic |  |
| 25 | 3729754 |  | intergenic |  |
| 25 | 3729893 |  | intergenic |  |
| 25 | 3730575 |  | intergenic |  |
| 25 | 3731330 |  | intergenic |  |
| 25 | 3731472 |  | intergenic |  |
| 25 | 3731475 |  | intergenic |  |
| 25 | 3732592 |  | intergenic |  |
| 25 | 3733984 |  | intergenic |  |
| 25 | 3735504 |  | intergenic |  |
| 25 | 3737435 |  | intergenic |  |
| 25 | 3737811 |  | intergenic |  |
| 25 | 3737881 |  | intergenic |  |
| 25 | 3742822 |  | intergenic |  |
| 25 | 3743590 |  | intergenic |  |
| 25 | 3745132 |  | intergenic |  |
| 25 | 3745856 |  | intergenic |  |
| 25 | 3806841 | ENSECAG00000014350 (pseudogene) | upstream | 500bp upstream |
| 25 | 3808633 | ENSECAG00000014350 (pseudogene) | downstream | 500bp downstream |
| 25 | 3821311 |  | intergenic |  |
| 25 | 3825063 |  | intergenic |  |
| 25 | 3825175 |  | intergenic |  |
| 25 | 3825922 |  | intergenic |  |
| 25 | 3828883 |  | intergenic |  |
| 25 | 3829194 |  | intergenic |  |
| 25 | 3829575 |  | intergenic |  |
| 25 | 3830853 |  | intergenic |  |
| 25 | 3830993 |  | intergenic |  |
| 25 | 3831043 |  | intergenic |  |
| 25 | 3831485 |  | intergenic |  |
| 25 | 3831737 |  | intergenic |  |
| 25 | 3832345 |  | intergenic |  |
| 25 | 3832490 |  | intergenic |  |
| 25 | 3832590 |  | intergenic |  |
| 25 | 3832942 |  | intergenic |  |
| 25 | 3833193 |  | intergenic |  |
| 25 | 3833203 |  | intergenic |  |
| 25 | 3833565 |  | intergenic |  |
| 25 | 3833769 |  | intergenic |  |
| 25 | 3833777 |  | intergenic |  |
| 25 | 3834041 |  | intergenic |  |
| 25 | 3834997 |  | intergenic |  |
| 25 | 3835069 |  | intergenic |  |
| 25 | 3835149 |  | intergenic |  |
| 25 | 3835341 |  | intergenic |  |
| 25 | 3835647 |  | intergenic |  |
| 25 | 3835760 |  | intergenic |  |
| 25 | 3835931 |  | intergenic |  |
| 25 | 3835991 |  | intergenic |  |
| 25 | 3836216 |  | intergenic |  |
| 25 | 3836635 |  | intergenic |  |
| 25 | 3836643 |  | intergenic |  |
| 25 | 3836667 |  | intergenic |  |
| 25 | 3837150 |  | intergenic |  |
| 25 | 3837838 |  | intergenic |  |
| 25 | 3838065 |  | intergenic |  |
| 25 | 3838771 |  | intergenic |  |
| 25 | 3838792 |  | intergenic |  |
| 25 | 3838841 |  | intergenic |  |
| 25 | 3839157 |  | intergenic |  |
| 25 | 3840088 |  | intergenic |  |
| 25 | 3840155 |  | intergenic |  |
| 25 | 3840197 |  | intergenic |  |
| 25 | 3840239 |  | intergenic |  |
| 25 | 3840349 |  | intergenic |  |
| 25 | 3840601 |  | intergenic |  |
| 25 | 3841435 |  | intergenic |  |
| 25 | 3841581 |  | intergenic |  |
| 25 | 3842030 |  | intergenic |  |
| 25 | 3842107 |  | intergenic |  |
| 25 | 3842115 |  | intergenic |  |
| 25 | 3842160 |  | intergenic |  |
| 25 | 3842369 |  | intergenic |  |
| 25 | 3842431 |  | intergenic |  |
| 25 | 3842820 |  | intergenic |  |
| 25 | 3843487 |  | intergenic |  |
| 25 | 3843885 |  | intergenic |  |
| 25 | 3844186 |  | intergenic |  |
| 25 | 3844212 |  | intergenic |  |
| 25 | 3844408 |  | intergenic |  |
| 25 | 3844541 |  | intergenic |  |
| 25 | 3844556 |  | intergenic |  |
| 25 | 3844681 |  | intergenic |  |
| 25 | 3845289 |  | intergenic |  |
| 25 | 3845598 |  | intergenic |  |
| 25 | 3846319 |  | intergenic |  |
| 25 | 3846478 |  | intergenic |  |
| 25 | 3846535 |  | intergenic |  |
| 25 | 3846668 |  | intergenic |  |
| 25 | 3846799 |  | intergenic |  |
| 25 | 3847033 |  | intergenic |  |
| 25 | 3847681 |  | intergenic |  |
| 25 | 3847888 |  | intergenic |  |
| 25 | 3850842 |  | intergenic |  |
| 25 | 3851047 |  | intergenic |  |
| 25 | 3851219 |  | intergenic |  |
| 25 | 3851670 |  | intergenic |  |
| 25 | 3852128 |  | intergenic |  |
| 25 | 3852381 |  | intergenic |  |
| 25 | 3854650 |  | intergenic |  |
| 25 | 3854791 |  | intergenic |  |
| 25 | 3854887 |  | intergenic |  |
| 25 | 3855178 |  | intergenic |  |
| 25 | 3855548 |  | intergenic |  |
| 25 | 3856407 |  | intergenic |  |
| 25 | 3856410 |  | intergenic |  |
| 25 | 3856444 |  | intergenic |  |
| 25 | 3856448 |  | intergenic |  |
| 25 | 3857345 |  | intergenic |  |
| 25 | 3857347 |  | intergenic |  |
| 25 | 3858376 |  | intergenic |  |
| 25 | 3858753 |  | intergenic |  |
| 25 | 3858768 |  | intergenic |  |
| 25 | 3858814 |  | intergenic |  |
| 25 | 3859117 |  | intergenic |  |
| 25 | 3859726 |  | intergenic |  |
| 25 | 3859943 |  | intergenic |  |
| 25 | 3859961 |  | intergenic |  |
| 25 | 3860032 |  | intergenic |  |
| 25 | 3860041 |  | intergenic |  |
| 25 | 3860105 |  | intergenic |  |
| 25 | 3860127 |  | intergenic |  |
| 25 | 3860132 |  | intergenic |  |
| 25 | 3860478 |  | intergenic |  |
| 25 | 3860673 |  | intergenic |  |
| 25 | 3861259 |  | intergenic |  |
| 25 | 3861680 |  | intergenic |  |
| 25 | 3861725 |  | intergenic |  |
| 25 | 3861749 |  | intergenic |  |
| 25 | 3861750 |  | intergenic |  |
| 25 | 3861756 |  | intergenic |  |
| 25 | 3862043 |  | intergenic |  |
| 25 | 3862296 |  | intergenic |  |
| 25 | 3862305 |  | intergenic |  |
| 25 | 3862628 |  | intergenic |  |
| 25 | 3862662 |  | intergenic |  |
| 25 | 3862920 |  | intergenic |  |
| 25 | 3862976 |  | intergenic |  |
| 25 | 3863046 |  | intergenic |  |
| 25 | 3863053 |  | intergenic |  |
| 25 | 3863149 |  | intergenic |  |
| 25 | 3863164 |  | intergenic |  |
| 25 | 3863179 |  | intergenic |  |
| 25 | 3863479 |  | intergenic |  |
| 25 | 3863615 |  | intergenic |  |
| 25 | 3863826 |  | intergenic |  |
| 25 | 3863832 |  | intergenic |  |
| 25 | 3863894 |  | intergenic |  |
| 25 | 3863969 |  | intergenic |  |
| 25 | 3864014 |  | intergenic |  |
| 25 | 3864085 |  | intergenic |  |
| 25 | 3864124 |  | intergenic |  |
| 25 | 3864125 |  | intergenic |  |
| 25 | 3864136 |  | intergenic |  |
| 25 | 3864386 |  | intergenic |  |
| 25 | 3864398 |  | intergenic |  |
| 25 | 3864768 |  | intergenic |  |
| 25 | 3864904 |  | intergenic |  |
| 25 | 3865184 |  | intergenic |  |
| 25 | 3865819 |  | intergenic |  |
| 25 | 3865901 |  | intergenic |  |
| 25 | 3866146 |  | intergenic |  |
| 25 | 3866554 |  | intergenic |  |
| 25 | 3866568 |  | intergenic |  |
| 25 | 3866821 |  | intergenic |  |
| 25 | 3866877 |  | intergenic |  |
| 25 | 3866909 |  | intergenic |  |
| 25 | 3866928 |  | intergenic |  |
| 25 | 3866931 |  | intergenic |  |
| 25 | 3866940 |  | intergenic |  |
| 25 | 3867210 |  | intergenic |  |
| 25 | 3867236 |  | intergenic |  |
| 25 | 3867265 |  | intergenic |  |
| 25 | 3867300 |  | intergenic |  |
| 25 | 3867730 |  | intergenic |  |
| 25 | 3867761 |  | intergenic |  |
| 25 | 3867809 |  | intergenic |  |
| 25 | 3868227 |  | intergenic |  |
| 25 | 3868230 |  | intergenic |  |
| 25 | 3868319 |  | intergenic |  |
| 25 | 3868362 |  | intergenic |  |
| 25 | 3869191 |  | intergenic |  |
| 25 | 3869530 |  | intergenic |  |
| 25 | 3869631 |  | intergenic |  |
| 25 | 3869837 |  | intergenic |  |
| 25 | 3869932 |  | intergenic |  |
| 25 | 3869946 |  | intergenic |  |
| 25 | 3869992 |  | intergenic |  |
| 25 | 3869993 |  | intergenic |  |
| 25 | 3870022 |  | intergenic |  |
| 25 | 3870069 |  | intergenic |  |
| 25 | 3870130 |  | intergenic |  |
| 25 | 3870273 |  | intergenic |  |
| 25 | 3870731 |  | intergenic |  |
| 25 | 3870964 |  | intergenic |  |
| 25 | 3870991 |  | intergenic |  |
| 25 | 3871086 |  | intergenic |  |
| 25 | 3871162 |  | intergenic |  |
| 25 | 3871449 |  | intergenic |  |
| 25 | 3872175 |  | intergenic |  |
| 25 | 3872302 |  | intergenic |  |
| 25 | 3872338 |  | intergenic |  |
| 25 | 3872717 |  | intergenic |  |
| 25 | 3872742 |  | intergenic |  |
| 25 | 3872861 |  | intergenic |  |
| 25 | 3872937 |  | intergenic |  |
| 25 | 3873159 |  | intergenic |  |
| 25 | 3874457 |  | intergenic |  |
| 25 | 11781861 | TMEM38B | upstream | ~3kb upstream |
| 25 | 11783623 | TMEM38B | upstream | ~1kb upstream |
| 25 | 11785232 | TMEM38B | intron | 132bp from jxn |
| 25 | 11785251 | TMEM38B | intron | 151bp from jxn |
| 25 | 11785357 | TMEM38B | intron | 257bp from jxn |
| 25 | 11785728 | TMEM38B | intron | 728bp from jxn |
| 25 | 11785946 | TMEM38B | intron | 9+kb intron |
| 25 | 11787079 | TMEM38B | intron | 9+kb intron |
| 25 | 11787236 | TMEM38B | intron | 9+kb intron |
| 25 | 11787257 | TMEM38B | intron | 9+kb intron |
| 25 | 11787280 | TMEM38B | intron | 9+kb intron |
| 25 | 11787376 | TMEM38B | intron | 9+kb intron |
| 25 | 11787637 | TMEM38B | intron | 9+kb intron |
| 25 | 11788232 | TMEM38B | intron | 9+kb intron |
| 25 | 11788233 | TMEM38B | intron | 9+kb intron |
| 25 | 11788268 | TMEM38B | intron | 9+kb intron |
| 25 | 11788701 | TMEM38B | intron | 9+kb intron |
| 25 | 11789258 | TMEM38B | intron | 9+kb intron |
| 25 | 11790106 | TMEM38B | intron | 9+kb intron |
| 25 | 11790843 | TMEM38B | intron | 9+kb intron |
| 25 | 11791562 | TMEM38B | intron | 9+kb intron |
| 25 | 11791872 | TMEM38B | intron | 9+kb intron |
| 25 | 11792134 | TMEM38B | intron | 9+kb intron |
| 25 | 11792396 | TMEM38B | intron | 9+kb intron |
| 25 | 11793228 | TMEM38B | intron | 9+kb intron |
| 25 | 11794790 | TMEM38B | intron | 50b from jxn |
| 25 | 11796037 | TMEM38B | intron | ~400bp from jxn |
| 25 | 11797092 | TMEM38B | intron | 4+kb intron |
| 25 | 11797880 | TMEM38B | intron | 4+kb intron |
| 25 | 11799319 | TMEM38B | intron | 4+kb intron |
| 25 | 11799738 | TMEM38B | intron | 4+kb intron |
| 25 | 11799848 | TMEM38B | intron | 250bp from jxn |
| 25 | 11800074 | TMEM38B | exon | exon 4 |
| 25 | 11800443 | TMEM38B | intron | ~400bp from jxn |
| 25 | 11801203 | TMEM38B | intron | 11+kb intron |
| 25 | 11801373 | TMEM38B | intron | 11+kb intron |
| 25 | 11801455 | TMEM38B | intron | 11+kb intron |
| 25 | 11801664 | TMEM38B | intron | 11+kb intron |
| 25 | 11802141 | TMEM38B | intron | 11+kb intron |
| 25 | 11802532 | TMEM38B | intron | 11+kb intron |
| 25 | 11802733 | TMEM38B | intron | 11+kb intron |
| 25 | 11803413 | TMEM38B | intron | 11+kb intron |
| 25 | 11803460 | TMEM38B | intron | 11+kb intron |
| 25 | 11804450 | TMEM38B | intron | 11+kb intron |
| 25 | 11804661 | TMEM38B | intron | 11+kb intron |
| 25 | 11804684 | TMEM38B | intron | 11+kb intron |
| 25 | 11805040 | TMEM38B | intron | 11+kb intron |
| 25 | 11805076 | TMEM38B | intron | 11+kb intron |
| 25 | 11805422 | TMEM38B | intron | 11+kb intron |
| 25 | 11805536 | TMEM38B | intron | 11+kb intron |
| 25 | 11805654 | TMEM38B | intron | 11+kb intron |
| 25 | 11806145 | TMEM38B | intron | 11+kb intron |
| 25 | 11806756 | TMEM38B | intron | 11+kb intron |
| 25 | 11806933 | TMEM38B | intron | 11+kb intron |
| 25 | 11806948 | TMEM38B | intron | 11+kb intron |
| 25 | 11807466 | TMEM38B | intron | 11+kb intron |
| 25 | 11807561 | TMEM38B | intron | 11+kb intron |
| 25 | 11807689 | TMEM38B | intron | 11+kb intron |
| 25 | 11808066 | TMEM38B | intron | 11+kb intron |
| 25 | 11808081 | TMEM38B | intron | 11+kb intron |
| 25 | 11808126 | TMEM38B | intron | 11+kb intron |
| 25 | 11808586 | TMEM38B | intron | 11+kb intron |
| 25 | 11808704 | TMEM38B | intron | 11+kb intron |
| 25 | 11809151 | TMEM38B | intron | 11+kb intron |
| 25 | 11809796 | TMEM38B | intron | 11+kb intron |
| 25 | 11810216 | TMEM38B | intron | 11+kb intron |
| 25 | 11810288 | TMEM38B | intron | 11+kb intron |
| 25 | 11810565 | TMEM38B | intron | 11+kb intron |
| 25 | 11810640 | TMEM38B | intron | 11+kb intron |
| 25 | 11810764 | TMEM38B | intron | 11+kb intron |
| 25 | 11811652 | TMEM38B | intron | 22bp from jxn |
| 25 | 11811829 | TMEM38B | exon | exon 5 |
| 25 | 11812413 | TMEM38B | downstream | 300bp downstream |
| 25 | 11812456 | TMEM38B | downstream | 300bp downstream |
| 25 | 11812483 | TMEM38B | downstream | 300bp downstream |
| 25 | 11813036 | TMEM38B | downstream | ~1kb downstream |
| 25 | 11813117 | TMEM38B | downstream | ~1kb downstream |
| 25 | 11813165 | TMEM38B | downstream | ~1kb downstream |
| 25 | 11813491 | TMEM38B | downstream | ~1kb downstream |
| 25 | 11813946 | TMEM38B | downstream | ~1kb downstream |
| 25 | 11814094 | TMEM38B | downstream | ~2kb downstream |
| 25 | 11814126 | TMEM38B | downstream | ~2kb downstream |
| 25 | 11814405 | TMEM38B | downstream | ~2kb downstream |
| 25 | 11814792 | TMEM38B | downstream | ~2kb downstream |
| 25 | 11814820 | TMEM38B | downstream | ~2kb downstream |
| 25 | 11815439 | TMEM38B | downstream | ~3kb downstream |
| 25 | 11815449 | TMEM38B | downstream | ~3kb downstream |
| 25 | 11815477 | TMEM38B | downstream | ~3kb downstream |
| 25 | 11815495 | TMEM38B | downstream | ~3kb downstream |
| 25 | 11815516 | TMEM38B | downstream | ~3kb downstream |
| 25 | 11815723 | TMEM38B | downstream | ~3kb downstream |
| 25 | 11816285 | TMEM38B | downstream | ~4kb downstream |
| 25 | 11816366 | TMEM38B | downstream | ~4kb downstream |
| 25 | 11816587 | TMEM38B | downstream | ~4kb downstream |
| 25 | 11818147 | TMEM38B | downstream | ~6kb downstream |
| 25 | 11818431 | TMEM38B | downstream | ~6kb downstream |
| 25 | 11841473 |  | intergenic |  |
| 25 | 11842403 |  | intergenic |  |
| 25 | 11843909 |  | intergenic |  |
| 25 | 11843953 |  | intergenic |  |
| 25 | 11843961 |  | intergenic |  |
| 25 | 11844181 |  | intergenic |  |
| 25 | 11844759 |  | intergenic |  |
| 25 | 11845467 |  | intergenic |  |
| 25 | 11845603 |  | intergenic |  |
| 25 | 15026761 |  | intergenic |  |
| 25 | 15033135 |  | intergenic |  |
| 25 | 15033359 |  | intergenic |  |
| 25 | 15034274 |  | intergenic |  |
| 25 | 15038211 |  | intergenic |  |
| 25 | 15038634 |  | intergenic |  |
| 25 | 15038640 |  | intergenic |  |
| 25 | 15040118 |  | intergenic |  |
| 25 | 15040276 |  | intergenic |  |
| 25 | 15040480 |  | intergenic |  |
| 25 | 15040925 |  | intergenic |  |
| 25 | 15041276 |  | intergenic |  |
| 25 | 15041982 |  | intergenic |  |
| 25 | 15042151 |  | intergenic |  |
| 25 | 15042636 |  | intergenic |  |
| 25 | 15043248 |  | intergenic |  |
| 25 | 15043424 |  | intergenic |  |
| 25 | 15044375 |  | intergenic |  |
| 25 | 15044553 |  | intergenic |  |
| 25 | 15055145 |  | intergenic |  |
| 25 | 15063700 |  | intergenic |  |
| 25 | 15064027 |  | intergenic |  |
| 25 | 15066566 |  | intergenic |  |
| 29 | 3188987 |  | intergenic |  |
| 29 | 3192353 |  | intergenic |  |
| 29 | 3203575 |  | intergenic |  |
| 29 | 3259807 | CUL2 | intron | 9kb intron |
| 29 | 3281313 | CUL2 | intron | 19kb intron |
| 29 | 3291497 | CUL2 | intron | 82bp from jxn |
| 29 | 3326059 |  | intergenic |  |
| 29 | 3326764 |  | intergenic |  |
| 29 | 3327733 |  | intergenic |  |
| 29 | 3327914 |  | intergenic |  |
| 29 | 3328294 |  | intergenic |  |
| 29 | 3328368 |  | intergenic |  |
| 29 | 3329490 |  | intergenic |  |
| 29 | 3329496 |  | intergenic |  |
| 29 | 3340343 |  | intergenic |  |
| 29 | 3349746 |  | intergenic |  |
| 29 | 3361183 |  | intergenic |  |
| 29 | 3361569 |  | intergenic |  |
| 29 | 3367198 |  | intergenic |  |
| 29 | 3380287 |  | intergenic |  |
| 29 | 3406312 |  | intergenic |  |
| 29 | 3407229 |  | intergenic |  |
| 29 | 3432936 |  | intergenic |  |
| 29 | 3447596 |  | intergenic |  |
| 29 | 3447706 |  | intergenic |  |
| 29 | 3447708 |  | intergenic |  |
| 29 | 3447766 |  | intergenic |  |
| 29 | 3447795 |  | intergenic |  |
| 29 | 3447992 |  | intergenic |  |
| 29 | 3451750 |  | intergenic |  |
| 29 | 3454214 |  | intergenic |  |
| 29 | 3454653 |  | intergenic |  |
| 29 | 3454674 |  | intergenic |  |
| 29 | 3468920 |  | intergenic |  |
| 29 | 3469807 |  | intergenic |  |
| 29 | 3470001 |  | intergenic |  |
| 29 | 3470018 |  | intergenic |  |
| 29 | 3471092 |  | intergenic |  |
| 29 | 3471572 |  | intergenic |  |
| 29 | 10054372 |  | intergenic |  |
| 29 | 10074576 | ABI1 | intron | 450bp from jxn |
| 29 | 10074859 | ABI1 | intron | 750bp from jxn |
| 29 | 10086818 | ABI1 | intron | 38kb intron |
| 29 | 10087117 | ABI1 | intron | 38kb intron |
| 29 | 10088749 | ABI1 | intron | 38kb intron |
| 29 | 10109015 | ABI1 | intron | 38kb intron |
| 30 | 14059751 | RRP15 | intron | 13kb intron |
| 30 | 14064781 | RRP15 | intron | 13kb intron |
| 30 | 14067984 | RRP15 | upstream | 750bp upstream |
| 30 | 14071641 | RRP15 | upstream | ~4kb upstream |
| 30 | 14075072 |  | intergenic |  |
| 30 | 14077332 |  | intergenic |  |
| 30 | 14078203 |  | intergenic |  |
| 30 | 14079314 |  | intergenic |  |
| 30 | 14079515 |  | intergenic |  |
| 30 | 14079516 |  | intergenic |  |
| 30 | 14079517 |  | intergenic |  |
| 30 | 14079688 |  | intergenic |  |
| 30 | 14079801 |  | intergenic |  |
| 30 | 14079847 |  | intergenic |  |
| 30 | 14079859 |  | intergenic |  |
| 30 | 14079872 |  | intergenic |  |
| 30 | 14079896 |  | intergenic |  |
| 30 | 14081214 |  | intergenic |  |
| 30 | 14082037 |  | intergenic |  |
| 30 | 14085786 |  | intergenic |  |
| 30 | 14086058 |  | intergenic |  |
| 30 | 14086261 |  | intergenic |  |
| 30 | 14089518 |  | intergenic |  |
| 30 | 14092124 |  | intergenic |  |
| 30 | 14093539 |  | intergenic |  |
| 30 | 14097309 |  | intergenic |  |
| 30 | 14105600 |  | intergenic |  |
| 30 | 14107178 |  | intergenic |  |
| 30 | 14108607 |  | intergenic |  |
| 30 | 14109560 |  | intergenic |  |
| 30 | 14110483 |  | intergenic |  |
| 30 | 14111432 |  | intergenic |  |
| 30 | 14112632 |  | intergenic |  |
| 30 | 14112770 |  | intergenic |  |
| 30 | 14121252 |  | intergenic |  |
| 30 | 14122738 |  | intergenic |  |
| 30 | 14124917 |  | intergenic |  |
| 30 | 14901107 |  | intergenic |  |
| 30 | 14903544 |  | intergenic |  |
| 30 | 14910466 |  | intergenic |  |
| 30 | 14910467 |  | intergenic |  |
| 30 | 14910620 |  | intergenic |  |
| 30 | 14913294 |  | intergenic |  |
| 30 | 14920073 |  | intergenic |  |
| 30 | 14921812 |  | intergenic |  |
| 30 | 14922459 |  | intergenic |  |
| 30 | 14922728 |  | intergenic |  |
| 30 | 14931350 |  | intergenic |  |
| 30 | 14932156 |  | intergenic |  |
| 30 | 14933228 |  | intergenic |  |
| 30 | 14933898 |  | intergenic |  |
| 30 | 14934049 |  | intergenic |  |
| 30 | 14935293 |  | intergenic |  |
| 30 | 14936139 |  | intergenic |  |
| 30 | 14937189 |  | intergenic |  |
| 30 | 14937630 |  | intergenic |  |
| 30 | 14937934 |  | intergenic |  |
| 30 | 14938148 |  | intergenic |  |
| 30 | 14938377 |  | intergenic |  |
| 30 | 14939137 |  | intergenic |  |
| 30 | 14939416 |  | intergenic |  |
| 30 | 14939422 |  | intergenic |  |
| 30 | 14940520 |  | intergenic |  |
| 30 | 14940634 |  | intergenic |  |
| 30 | 14940945 |  | intergenic |  |
| 30 | 14941667 |  | intergenic |  |
| 30 | 14942713 |  | intergenic |  |
| 30 | 14943174 |  | intergenic |  |
| 30 | 14944096 |  | intergenic |  |
| 30 | 14944397 |  | intergenic |  |
| 30 | 14944668 |  | intergenic |  |
| 30 | 14944670 |  | intergenic |  |
| 30 | 14944742 |  | intergenic |  |
| 30 | 14946411 |  | intergenic |  |
| 30 | 14947260 |  | intergenic |  |
| 30 | 14947386 |  | intergenic |  |
| 30 | 14947398 |  | intergenic |  |
| 30 | 14947399 |  | intergenic |  |
| 30 | 14947424 |  | intergenic |  |
| 30 | 14947444 |  | intergenic |  |
| 30 | 14947553 |  | intergenic |  |
| 30 | 14947850 |  | intergenic |  |
| 30 | 14947886 |  | intergenic |  |
| 30 | 14948778 |  | intergenic |  |
| 30 | 14950352 |  | intergenic |  |
| 30 | 14950947 |  | intergenic |  |
| 30 | 14951559 |  | intergenic |  |
| 30 | 14951633 |  | intergenic |  |
| 30 | 14953064 |  | intergenic |  |
| 30 | 14953633 |  | intergenic |  |
| 30 | 14953646 |  | intergenic |  |
| 30 | 14953989 |  | intergenic |  |
| 30 | 14954702 |  | intergenic |  |
| 30 | 14955631 |  | intergenic |  |
| 30 | 14955684 |  | intergenic |  |
| 30 | 14957094 |  | intergenic |  |
| 30 | 14957468 |  | intergenic |  |
| 30 | 14957813 |  | intergenic |  |
| 30 | 14958679 |  | intergenic |  |
| 30 | 14959634 |  | intergenic |  |
| 30 | 14966802 |  | intergenic |  |
| 30 | 14968084 |  | intergenic |  |
| 30 | 14968208 |  | intergenic |  |
| 30 | 14968345 |  | intergenic |  |
| 30 | 14969489 |  | intergenic |  |
| 30 | 14969793 |  | intergenic |  |
| 30 | 14971185 |  | intergenic |  |
| 30 | 14974990 |  | intergenic |  |
| 30 | 14975505 |  | intergenic |  |
| 30 | 14975635 |  | intergenic |  |
| 30 | 14975647 |  | intergenic |  |
| 30 | 14975870 |  | intergenic |  |
| 30 | 14975937 |  | intergenic |  |
| 30 | 14976135 |  | intergenic |  |
| 30 | 14976253 |  | intergenic |  |
| 30 | 14976260 |  | intergenic |  |
| 30 | 14977353 |  | intergenic |  |
| 30 | 14977437 |  | intergenic |  |
| 30 | 14977522 |  | intergenic |  |
| 30 | 14977917 |  | intergenic |  |
| 30 | 14978003 |  | intergenic |  |
| 30 | 14978013 |  | intergenic |  |
| 30 | 14978121 |  | intergenic |  |
| 30 | 14978135 |  | intergenic |  |
| 30 | 14978523 |  | intergenic |  |
| 30 | 14979754 |  | intergenic |  |
| 30 | 14981933 |  | intergenic |  |
| 30 | 14984512 |  | intergenic |  |
| 30 | 14984827 |  | intergenic |  |
| 30 | 14988013 |  | intergenic |  |
| 30 | 14990682 |  | intergenic |  |
| 30 | 14991990 |  | intergenic |  |
| 30 | 14992221 |  | intergenic |  |
| 30 | 14992763 |  | intergenic |  |
| 30 | 14993739 |  | intergenic |  |
| 30 | 14993793 |  | intergenic |  |
| 30 | 14998469 |  | intergenic |  |
| 30 | 14998665 |  | intergenic |  |
| 30 | 14999013 |  | intergenic |  |
| 30 | 15050706 |  | intergenic |  |
| 30 | 15050933 |  | intergenic |  |
| 30 | 15050946 |  | intergenic |  |
| 30 | 15052072 |  | intergenic |  |
| 30 | 15052347 |  | intergenic |  |
| 30 | 15052387 |  | intergenic |  |
| 30 | 15052589 |  | intergenic |  |
| 30 | 15052637 |  | intergenic |  |
| 30 | 15052892 |  | intergenic |  |
| 30 | 15053100 |  | intergenic |  |
| 30 | 15053101 |  | intergenic |  |
| 30 | 15053601 |  | intergenic |  |
| 30 | 15053939 |  | intergenic |  |
| 30 | 15055259 |  | intergenic |  |
| 30 | 15055711 |  | intergenic |  |
| 30 | 15055716 |  | intergenic |  |
| 30 | 15055793 |  | intergenic |  |
| 30 | 15055805 |  | intergenic |  |
| 30 | 15055838 |  | intergenic |  |
| 30 | 15056048 |  | intergenic |  |
| 30 | 15056520 |  | intergenic |  |
| 30 | 15057299 |  | intergenic |  |
| 30 | 15057326 |  | intergenic |  |
| 30 | 15057340 |  | intergenic |  |
| 30 | 15057392 |  | intergenic |  |
| 30 | 15058243 |  | intergenic |  |
| 30 | 15058448 |  | intergenic |  |
| 30 | 15060048 |  | intergenic |  |
| 30 | 15060135 |  | intergenic |  |
| 30 | 15061000 |  | intergenic |  |
| 30 | 15063569 |  | intergenic |  |
| 30 | 15064842 |  | intergenic |  |
| 30 | 15067185 |  | intergenic |  |
| 30 | 15068782 |  | intergenic |  |
| 30 | 15069661 |  | intergenic |  |
| 30 | 15070109 |  | intergenic |  |
| 30 | 15070425 |  | intergenic |  |
| 30 | 15070482 |  | intergenic |  |
| 30 | 15071169 |  | intergenic |  |
| 30 | 15071362 |  | intergenic |  |
| 30 | 15071589 |  | intergenic |  |
| 30 | 15071789 |  | intergenic |  |
| 30 | 15071848 |  | intergenic |  |
| 30 | 15072347 |  | intergenic |  |
| 30 | 15072710 |  | intergenic |  |
| 30 | 15073300 |  | intergenic |  |
| 30 | 15076073 |  | intergenic |  |
| 30 | 15076957 |  | intergenic |  |
| 30 | 15077321 |  | intergenic |  |
| 30 | 15079103 |  | intergenic |  |
| 30 | 15079295 |  | intergenic |  |
| 30 | 15080376 |  | intergenic |  |
| 30 | 15081491 |  | intergenic |  |
| 30 | 15084304 |  | intergenic |  |
| 30 | 15087772 |  | intergenic |  |
| 30 | 15088703 |  | intergenic |  |
| 30 | 15090133 |  | intergenic |  |
| 30 | 15091257 |  | intergenic |  |
| 30 | 15093121 |  | intergenic |  |
| 30 | 15093201 |  | intergenic |  |
| 30 | 15093464 |  | intergenic |  |
| 30 | 15093731 |  | intergenic |  |
| 30 | 15094795 |  | intergenic |  |
| 30 | 15098919 |  | intergenic |  |
| 30 | 15099440 |  | intergenic |  |
| 30 | 15099814 |  | intergenic |  |
| 30 | 15103249 |  | intergenic |  |
| 30 | 15105294 |  | intergenic |  |
| 30 | 15107129 |  | intergenic |  |
| 30 | 15108664 |  | intergenic |  |
| 30 | 15109043 |  | intergenic |  |
| 30 | 15111612 |  | intergenic |  |
| 30 | 15112727 |  | intergenic |  |
| 30 | 15113529 |  | intergenic |  |
| 30 | 15115462 |  | intergenic |  |
| 30 | 15115513 |  | intergenic |  |
| 30 | 15115920 |  | intergenic |  |
| 30 | 15116154 |  | intergenic |  |
| 30 | 15116214 |  | intergenic |  |
| 30 | 15117819 |  | intergenic |  |
| 30 | 15119404 |  | intergenic |  |
| 30 | 15119414 |  | intergenic |  |
| 30 | 15119699 |  | intergenic |  |
| 30 | 15120606 |  | intergenic |  |
| 30 | 15122001 |  | intergenic |  |
| 30 | 15122089 |  | intergenic |  |
| 30 | 15124747 |  | intergenic |  |
