# Supplementary material for: Identification and validation of genetic variants predictive of gait in standardbred horses
Source: PLoS Genet. 2019 May 28;15(5):e1008146. doi: 10.1371/journal.pgen.1008146 (PMC6555539; doi:10.1371/journal.pgen.1008146)
Supplement: S4 Table — (DOCX) [file pgen.1008146.s004.docx]

**Supplemental Table 4:** Summary of 303 SNPs putatively associated with gait that were selected for inclusion in the Sequenom assay. The 190 SNPs selected from whole-genome sequencing were multiplexed with ancestry informative markers (AIMs) into groups of 48 in six wells; the 113 SNPs selected from pooled sequencing were multiplexed into two wells with 48 samples each and a third well with 17 samples (well assignments not shown). CHR = chromosome. SNPs were subsequently remapped to EquCab3.0 using BLAST (NCBI). Three SNPs that mapped uniquely to EquCab2 did not map uniquely to EquCab3.0; the most likely location for these SNPs in the new assembly is ECA17 at 37.07Mb.

| **SNPs selected from whole-genome sequencing** | | |  | **SNPs selected from pooled sequencing** | | |
| --- | --- | --- | --- | --- | --- | --- |
| **CHR** | **EquCab2** | **EquCab3** |  | **CHR** | **EquCab2** | **EquCab3** |
| 1 | 5322242 | 5348911 |  | 1 | 38563255 | 38812901 |
| 1 | 5532596 | 5560318 |  | 1 | 38573734 | 38823333 |
| 1 | 5532686 | 5560408 |  | 1 | 38837069 | 39087917 |
| 1 | 5632465 | 5660049 |  | 1 | 106883816 | 107798370 |
| 1 | 17548101 | 17666705 |  | 1 | 106928205 | 107842831 |
| 1 | 17552161 | 17670765 |  | 3 | 2489598 | 2622322 |
| 1 | 17617018 | 17736218 |  | 3 | 2506253 | 2638903 |
| 1 | 17945265 | 18064350 |  | 3 | 2521561 | 2654198 |
| 1 | 17955548 | 18074630 |  | 3 | 52318025 | 53791546 |
| 1 | 18065598 | 18183804 |  | 4 | 8996717 | 8997297 |
| 1 | 18109069 | 18227275 |  | 4 | 9091283 | 9091899 |
| 1 | 35670985 | 35919792 |  | 4 | 9116614 | 9117218 |
| 1 | 35720250 | 35969132 |  | 5 | 55291788 | 51871390 |
| 1 | 35721326 | 35970208 |  | 5 | 55301141 | 51880744 |
| 1 | 35726345 | 35975239 |  | 5 | 55317127 | 51896741 |
| 1 | 35729338 | 35978275 |  | 5 | 55333664 | 51913278 |
| 1 | 35731283 | 35980220 |  | 5 | 61126642 | 57708820 |
| 1 | 35731849 | 35980730 |  | 5 | 61144680 | 57726856 |
| 1 | 38306816 | 38556327 |  | 5 | 61163899 | 57746075 |
| 1 | 38591441 | 38841042 |  | 5 | 66187039 | 63016418 |
| 1 | 38592096 | 38841697 |  | 5 | 66199885 | 63029263 |
| 1 | 38592542 | 38842143 |  | 5 | 66221515 | 63050893 |
| 1 | 38599532 | 38849135 |  | 6 | 81299480 | 82463648 |
| 1 | 38646291 | 38895902 |  | 6 | 81651604 | 82815419 |
| 1 | 38987953 | 39324018 |  | 6 | 81668230 | 82832046 |
| 1 | 38988003 | 39324068 |  | 9 | 29141611 | 29922738 |
| 1 | 39070730 | 39406841 |  | 9 | 29155106 | 29936252 |
| 1 | 39102187 | 39438222 |  | 9 | 29211591 | 29992734 |
| 1 | 39376366 | 39712143 |  | 11 | 29532466 | 29792244 |
| 1 | 39376415 | 39712192 |  | 11 | 29564206 | 29824000 |
| 1 | 39560619 | 39896654 |  | 11 | 29599837 | 29860613 |
| 1 | 39589062 | 39925101 |  | 11 | 31303355 | 31602010 |
| 1 | 39691699 | 40027814 |  | 11 | 31319004 | 31617654 |
| 1 | 39772282 | 40111224 |  | 11 | 31470618 | 31769335 |
| 1 | 42307163 | 42648296 |  | 11 | 36608682 | 36903905 |
| 1 | 43245721 | 43587143 |  | 11 | 36669528 | 36964714 |
| 1 | 43245806 | 43587228 |  | 11 | 36714823 | 37009997 |
| 1 | 48293517 | 48656338 |  | 11 | 36775489 | 37070657 |
| 1 | 48896092 | 49259272 |  | 11 | 36796850 | 37092017 |
| 1 | 49602985 | 49965948 |  | 12 | 16262259 | 19692637 |
| 1 | 50226814 | 50590273 |  | 12 | 16270318 | 19700697 |
| 1 | 50226817 | 50590276 |  | 12 | 16380392 | 19810785 |
| 1 | 55106029 | 55552606 |  | 12 | 16412374 | 19842721 |
| 2 | 17358298 | 17406005 |  | 14 | 1368081 | 609235 |
| 2 | 17616769 | 17664266 |  | 14 | 1388861 | 630035 |
| 2 | 17690098 | 17737614 |  | 14 | 1403046 | 644220 |
| 2 | 18282822 | 18328458 |  | 14 | 1427118 | 668291 |
| 2 | 18364832 | 18410480 |  | 14 | 1570169 | 811504 |
| 2 | 18538576 | 18583657 |  | 14 | 5442438 | 4676988 |
| 2 | 18622200 | 18667236 |  | 15 | 10100242 | 10371732 |
| 2 | 18987527 | 19034724 |  | 15 | 10103035 | 10374525 |
| 2 | 19016749 | 19063948 |  | 15 | 10108201 | 10379691 |
| 2 | 19326982 | 19376230 |  | 16 | 59352686 | 60932530 |
| 2 | 19327652 | 19376900 |  | 16 | 59382124 | 60961901 |
| 2 | 19327821 | 19377069 |  | 16 | 59391893 | 60971671 |
| 2 | 19327827 | 19377075 |  | 17 | 50983052 | 50861533 |
| 2 | 19698739 | 19746752 |  | 17 | 51017875 | 50896356 |
| 2 | 19714056 | 19761885 |  | 17 | 51044268 | 50922749 |
| 2 | 19724672 | 19772501 |  | 17 | 61717590 | 61610225 |
| 2 | 19731591 | 19779420 |  | 17 | 61721785 | 61614420 |
| 2 | 19775173 | 19822898 |  | 17 | 61728019 | 61620654 |
| 3 | 2384676 | 2517303 |  | 17 | 61744016 | 61636653 |
| 3 | 2494992 | 2627642 |  | 17 | 61749334 | 61641971 |
| 3 | 3051017 | 3178607 |  | 17 | 65640738 | 65541767 |
| 3 | 3581003 | 3707443 |  | 17 | 65645147 | 65546176 |
| 3 | 3581548 | 3707987 |  | 17 | 65659694 | 65560722 |
| 3 | 3581676 | 3708115 |  | 20 | 25103556 | 25966714 |
| 3 | 3977000 | 4103035 |  | 20 | 25113918 | 25977075 |
| 3 | 46785415 | multi-map |  | 20 | 25127274 | 25990431 |
| 3 | 46785561 | multi-map |  | 20 | 27650699 | 28555103 |
| 3 | 46785621 | multi-map |  | 20 | 27691110 | 28595503 |
| 3 | 47817508 | 49197787 |  | 20 | 27711111 | 28615503 |
| 3 | 48135062 | 49515691 |  | 20 | 27727105 | 28631497 |
| 3 | 48816809 | 50197770 |  | 20 | 27768899 | 28673289 |
| 3 | 49488838 | 50869555 |  | 20 | 46929785 | 47924101 |
| 3 | 49530033 | 50910750 |  | 20 | 47062579 | 48056952 |
| 3 | 49601762 | 50982476 |  | 20 | 47092658 | 48087031 |
| 3 | 49601886 | 50982600 |  | 23 | 14640812 | 14008017 |
| 3 | 49601896 | 50982610 |  | 23 | 14645077 | 14012301 |
| 3 | 49785110 | 51165739 |  | 23 | 14649864 | 14017081 |
| 3 | 49857337 | 51237922 |  | 23 | 20639151 | 20022885 |
| 3 | 49857369 | 51237954 |  | 23 | 20652865 | 20036611 |
| 3 | 49857478 | 51238063 |  | 23 | 20658789 | 20042543 |
| 3 | 52563310 | 54036919 |  | 23 | 20662320 | 20046132 |
| 3 | 52680823 | 54154434 |  | 24 | 6712987 | 6575460 |
| 3 | 53257281 | 54732913 |  | 24 | 6736928 | 6599357 |
| 3 | 53721793 | 55197236 |  | 24 | 10276151 | 10133116 |
| 3 | 53834511 | 55309769 |  | 24 | 10285906 | 10142871 |
| 3 | 54166034 | 55639036 |  | 24 | 10296168 | 10153133 |
| 3 | 56561263 | 58034316 |  | 24 | 10299566 | 10156532 |
| 3 | 56755586 | 58228638 |  | 25 | 3657454 | 3704589 |
| 3 | 57629621 | 59104513 |  | 25 | 3666056 | 3713191 |
| 3 | 57929520 | 59404415 |  | 25 | 3694284 | 3741417 |
| 3 | 58044431 | 59519375 |  | 25 | 3724550 | 3771668 |
| 3 | 58070704 | 59545645 |  | 25 | 3860478 | 3909047 |
| 3 | 58077312 | 59552253 |  | 25 | 11783623 | 11832785 |
| 3 | 58174699 | 59649589 |  | 25 | 11800074 | 11849233 |
| 3 | 58434545 | 59909227 |  | 25 | 15026761 | 15426560 |
| 3 | 58903953 | 60646592 |  | 25 | 15044553 | 15446284 |
| 3 | 76844764 | 78652890 |  | 29 | 3291497 | 4306133 |
| 3 | 76844777 | 78652903 |  | 29 | 3447596 | 4462225 |
| 3 | 77739534 | 79548220 |  | 29 | 3471572 | 4486156 |
| 6 | 6609510 | 6384625 |  | 29 | 10074576 | 11097656 |
| 6 | 7832752 | 7607933 |  | 29 | 10087117 | 11110196 |
| 6 | 7841413 | 7616448 |  | 29 | 10109015 | 11132094 |
| 6 | 7881374 | 7656410 |  | 30 | 14059751 | 14895359 |
| 9 | 75715699 | 77822306 |  | 30 | 14067984 | 14903592 |
| 9 | 75803120 | 77909647 |  | 30 | 14107178 | 14942789 |
| 9 | 75813719 | 77920245 |  | 30 | 14936139 | 15771790 |
| 9 | 75816548 | 77923070 |  | 30 | 14947553 | 15783205 |
| 9 | 75896366 | 78003195 |  | 30 | 15055793 | 15891465 |
| 11 | 46845778 | 47167577 |  | 30 | 15068782 | 15904456 |
| 11 | 47003926 | 47323245 |  | 30 | 15124747 | 15960395 |
| 11 | 47016874 | 47336228 |  |  | | |
| 11 | 47016889 | 47336243 |  |  |  |  |
| 11 | 48341945 | 48676593 |  |  |  |  |
| 11 | 50634935 | 50978172 |  |  |  |  |
| 11 | 50846315 | 51191559 |  |  |  |  |
| 11 | 50918059 | 51263232 |  |  |  |  |
| 11 | 52424214 | 52774841 |  |  |  |  |
| 11 | 57422057 | 57773672 |  |  |  |  |
| 11 | 58376457 | 58728978 |  |  |  |  |
| 16 | 25722825 | 27185509 |  |  |  |  |
| 16 | 28197056 | 29659368 |  |  |  |  |
| 16 | 28198996 | 29661308 |  |  |  |  |
| 16 | 28199539 | 29661851 |  |  |  |  |
| 16 | 28786474 | 30289885 |  |  |  |  |
| 16 | 29331872 | 30835212 |  |  |  |  |
| 16 | 30331912 | 31834644 |  |  |  |  |
| 17 | 27685585 | 27588118 |  |  |  |  |
| 17 | 28054635 | 27956967 |  |  |  |  |
| 17 | 28293289 | 28195431 |  |  |  |  |
| 17 | 28347510 | 28249699 |  |  |  |  |
| 17 | 28361747 | 28263936 |  |  |  |  |
| 17 | 28458432 | 28360659 |  |  |  |  |
| 17 | 28485796 | 28388023 |  |  |  |  |
| 17 | 28540291 | 28442518 |  |  |  |  |
| 17 | 28658850 | 28560918 |  |  |  |  |
| 17 | 28658966 | 28561034 |  |  |  |  |
| 17 | 28711958 | 28613971 |  |  |  |  |
| 17 | 29271555 | 29173660 |  |  |  |  |
| 17 | 29274637 | 29176662 |  |  |  |  |
| 17 | 39403989 | 39307680 |  |  |  |  |
| 17 | 39404292 | 39307983 |  |  |  |  |
| 17 | 60460198 | 60351049 |  |  |  |  |
| 18 | 77603381 | 77713741 |  |  |  |  |
| 19 | 21446218 | 23822276 |  |  |  |  |
| 19 | 31393832 | 33925893 |  |  |  |  |
| 19 | 37986794 | 40570466 |  |  |  |  |
| 23 | 14182456 | 13549629 |  |  |  |  |
| 23 | 14211839 | 13579030 |  |  |  |  |
| 23 | 14648590 | 14015807 |  |  |  |  |
| 23 | 14714942 | 14082030 |  |  |  |  |
| 23 | 14813929 | 14181047 |  |  |  |  |
| 23 | 14814635 | 14181753 |  |  |  |  |
| 23 | 14980071 | 14347130 |  |  |  |  |
| 25 | 11689674 | 11694268 |  |  |  |  |
| 25 | 11691308 | 11738643 |  |  |  |  |
| 25 | 11793191 | 11740277 |  |  |  |  |
| 25 | 11811829 | 11842351 |  |  |  |  |
| 25 | 12758770 | 12771053 |  |  |  |  |
| 25 | 12791659 | 12808675 |  |  |  |  |
| 25 | 13016645 | 13066298 |  |  |  |  |
| 25 | 13021802 | 13071456 |  |  |  |  |
| 25 | 13021844 | 13071498 |  |  |  |  |
| 25 | 13031250 | 13080925 |  |  |  |  |
| 25 | 13031714 | 13081389 |  |  |  |  |
| 25 | 13036645 | 13086322 |  |  |  |  |
| 25 | 13047191 | 13096870 |  |  |  |  |
| 25 | 13050176 | 13099856 |  |  |  |  |
| 25 | 13052616 | 13102296 |  |  |  |  |
| 25 | 14418173 | 14757120 |  |  |  |  |
| 25 | 14531198 | 14757120 |  |  |  |  |
| 25 | 14557888 | 14896761 |  |  |  |  |
| 25 | 14735220 | 14870137 |  |  |  |  |
| 25 | 14737344 | 15074102 |  |  |  |  |
| 25 | 14760167 | 15076230 |  |  |  |  |
| 25 | 15576483 | 15661922 |  |  |  |  |
| 25 | 15621763 | 16001917 |  |  |  |  |
| 25 | 15829342 | 16084882 |  |  |  |  |
| 25 | 15839070 | 16233979 |  |  |  |  |
| 25 | 15845420 | 16243707 |  |  |  |  |
| 25 | 16817685 | 16344818 |  |  |  |  |
| 25 | 16820893 | 17226844 |  |  |  |  |
| 26 | 3315794 | 3711151 |  |  |  |  |
| 26 | 3315939 | 3711296 |  |  |  |  |
| 26 | 3315959 | 3711316 |  |  |  |  |
| 26 | 3315992 | 3711349 |  |  |  |  |
| 26 | 3641990 | 4037579 |  |  |  |  |
| 26 | 3642510 | 4038099 |  |  |  |  |
