# Supplementary material for: Identification and validation of genetic variants predictive of gait in standardbred horses
Source: PLoS Genet. 2019 May 28;15(5):e1008146. doi: 10.1371/journal.pgen.1008146 (PMC6555539; doi:10.1371/journal.pgen.1008146)
Supplement: S5 Table — (DOCX) [file pgen.1008146.s005.docx]

**Supplemental Table 5:** Summary of 177 statistically significant SNPs from GEMMA mixed model analysis in 720 Standardbred pacers and trotters genotyped on the custom Sequenom assay. After pruning, analysis included 245 SNPs. Uncorrected p-values are presented for the Wald test, the Likelihood ratio test (lrt) and the Score test. CHR = chromosome. SNPs were subsequently remapped to EquCab3.0 using BLAST (NCBI).

| Rank | CHR | EquCab2 | EquCab3 | p_wald | p_lrt | p_score |
| --- | --- | --- | --- | --- | --- | --- |
| 1 | 30 | 14067984 | 14903592 | 4.9E-39 | 1.4E-38 | 3.1E-31 |
| 2 | 30 | 14107178 | 14942789 | 1.2E-38 | 2.2E-38 | 3.5E-31 |
| 3 | 17 | 28540291 | 28442518 | 1.3E-38 | 2.3E-38 | 3.6E-31 |
| 4 | 17 | 28361747 | 28263936 | 2.3E-37 | 4.0E-37 | 2.2E-30 |
| 5 | 23 | 14640812 | 14008017 | 1.9E-37 | 1.0E-36 | 7.5E-30 |
| 6 | 23 | 14648590 | 14015807 | 2.0E-37 | 1.1E-36 | 8.2E-30 |
| 7 | 23 | 14649864 | 14017081 | 2.6E-37 | 1.2E-36 | 7.9E-30 |
| 8 | 30 | 14947553 | 15783205 | 2.1E-33 | 3.7E-33 | 1.1E-27 |
| 9 | 1 | 35729338 | 35978275 | 4.8E-33 | 9.7E-33 | 2.4E-27 |
| 10 | 1 | 35731849 | 35980730 | 5.5E-33 | 1.1E-32 | 2.6E-27 |
| 11 | 1 | 35731283 | 35980220 | 6.7E-33 | 1.3E-32 | 3.0E-27 |
| 12 | 1 | 35726345 | 35975239 | 9.8E-33 | 1.9E-32 | 3.7E-27 |
| 13 | 1 | 35720250 | 35969132 | 9.5E-33 | 1.9E-32 | 3.9E-27 |
| 14 | 30 | 15055793 | 15891465 | 1.3E-32 | 2.3E-32 | 4.0E-27 |
| 15 | 30 | 15068782 | 15904456 | 1.4E-32 | 2.5E-32 | 4.3E-27 |
| 16 | 17 | 28347510 | 28249699 | 4.2E-31 | 8.1E-31 | 5.3E-26 |
| 17 | 30 | 14936139 | 15771790 | 6.0E-30 | 1.1E-29 | 3.1E-25 |
| 18 | 1 | 17945265 | 18064350 | 1.1E-29 | 2.7E-29 | 7.6E-25 |
| 19 | 1 | 35721326 | 35970208 | 2.8E-29 | 5.3E-29 | 1.1E-24 |
| 20 | 17 | 28458432 | 28360659 | 1.9E-28 | 1.9E-28 | 1.8E-24 |
| 21 | 30 | 15124747 | 15960395 | 1.2E-27 | 1.4E-27 | 8.9E-24 |
| 22 | 3 | 3051017 | 3178607 | 8.6E-27 | 1.2E-26 | 4.9E-23 |
| 23 | 17 | 28658966 | 28561034 | 2.4E-26 | 2.5E-26 | 6.8E-23 |
| 24 | 23 | 20652865 | 20036611 | 1.4E-26 | 4.0E-26 | 1.8E-22 |
| 25 | 23 | 20662320 | 20046132 | 2.1E-26 | 6.6E-26 | 2.7E-22 |
| 26 | 20 | 27691110 | 28595503 | 1.4E-24 | 2.1E-24 | 2.5E-21 |
| 27 | 1 | 48896092 | 49259272 | 6.7E-24 | 6.6E-24 | 4.4E-21 |
| 28 | 25 | 11811829 | 11842351 | 4.5E-22 | 7.2E-22 | 2.3E-19 |
| 29 | 1 | 38573734 | 38823333 | 5.6E-22 | 9.2E-22 | 2.9E-19 |
| 30 | 17 | 29271555 | 29173660 | 9.4E-22 | 1.3E-21 | 3.4E-19 |
| 31 | 25 | 11783623 | 11832785 | 1.1E-21 | 1.6E-21 | 4.3E-19 |
| 32 | 30 | 14059751 | 14895359 | 3.4E-21 | 3.6E-21 | 6.1E-19 |
| 33 | 17 | 28658850 | 28560918 | 4.0E-21 | 4.5E-21 | 7.7E-19 |
| 34 | 25 | 11800074 | 11849233 | 4.8E-21 | 7.5E-21 | 1.5E-18 |
| 35 | 1 | 38591441 | 38841042 | 1.9E-20 | 2.6E-20 | 3.6E-18 |
| 36 | 1 | 38592542 | 38842143 | 3.2E-19 | 4.2E-19 | 3.4E-17 |
| 37 | 23 | 14645077 | 14012301 | 1.1E-18 | 1.8E-18 | 1.3E-16 |
| 38 | 25 | 15839070 | 16233979 | 1.7E-18 | 3.0E-18 | 1.9E-16 |
| 39 | 1 | 38306816 | 38556327 | 5.1E-18 | 7.0E-18 | 3.4E-16 |
| 40 | 3 | 2494992 | 2627642 | 8.0E-18 | 9.0E-18 | 3.6E-16 |
| 41 | 1 | 38592096 | 38841697 | 7.7E-18 | 1.0E-17 | 4.6E-16 |
| 42 | 16 | 28786474 | 30289885 | 9.3E-18 | 1.1E-17 | 4.3E-16 |
| 43 | 1 | 49602985 | 49965948 | 1.0E-16 | 1.0E-16 | 2.5E-15 |
| 44 | 25 | 15044553 | 15446284 | 1.0E-16 | 1.5E-16 | 4.6E-15 |
| 45 | 25 | 13031714 | 13081389 | 2.9E-16 | 2.9E-16 | 5.7E-15 |
| 46 | 1 | 38563255 | 38812901 | 4.1E-16 | 5.2E-16 | 1.1E-14 |
| 47 | 16 | 59391893 | 60971671 | 1.2E-15 | 1.2E-15 | 2.0E-14 |
| 48 | 17 | 51017875 | 50896356 | 1.2E-15 | 1.4E-15 | 2.4E-14 |
| 49 | 25 | 15845420 | 15845420 | 1.1E-15 | 1.6E-15 | 3.3E-14 |
| 50 | 16 | 59382124 | 60961901 | 2.1E-15 | 2.2E-15 | 3.3E-14 |
| 51 | 16 | 59352686 | 60932530 | 2.9E-15 | 3.0E-15 | 4.2E-14 |
| 52 | 20 | 27711111 | 28615503 | 3.3E-15 | 3.6E-15 | 5.1E-14 |
| 53 | 17 | 27685585 | 27588118 | 4.5E-15 | 4.7E-15 | 6.3E-14 |
| 54 | 25 | 16820893 | 17226844 | 5.3E-15 | 6.2E-15 | 8.7E-14 |
| 55 | 25 | 11793191 | 11740277 | 6.6E-15 | 8.5E-15 | 1.2E-13 |
| 56 | 20 | 25113918 | 25977075 | 1.9E-14 | 2.2E-14 | 2.6E-13 |
| 57 | 25 | 11689674 | 11694268 | 1.8E-14 | 2.3E-14 | 2.8E-13 |
| 58 | 3 | 2521561 | 2654198 | 2.5E-14 | 2.7E-14 | 2.9E-13 |
| 59 | 20 | 27650699 | 28555103 | 3.3E-14 | 3.6E-14 | 3.6E-13 |
| 60 | 3 | 53721793 | 55197236 | 6.1E-14 | 6.7E-14 | 6.3E-13 |
| 61 | 11 | 57422057 | 57773672 | 6.4E-14 | 7.1E-14 | 6.7E-13 |
| 62 | 11 | 36714823 | 37009997 | 8.0E-14 | 8.4E-14 | 7.4E-13 |
| 63 | 25 | 14418173 | 14757120 | 7.5E-14 | 9.4E-14 | 9.3E-13 |
| 64 | 11 | 36608682 | 36903905 | 1.4E-13 | 1.5E-13 | 1.3E-12 |
| 65 | 1 | 106928205 | 107842831 | 1.6E-13 | 1.7E-13 | 1.4E-12 |
| 66 | 4 | 9116614 | 9117218 | 1.6E-13 | 2.0E-13 | 1.8E-12 |
| 67 | 1 | 18065598 | 18183804 | 2.4E-13 | 2.5E-13 | 1.8E-12 |
| 68 | 25 | 11691308 | 11738643 | 1.9E-13 | 2.5E-13 | 2.3E-12 |
| 69 | 1 | 38646291 | 38895902 | 2.5E-13 | 2.7E-13 | 2.0E-12 |
| 70 | 23 | 14980071 | 14347130 | 3.7E-13 | 3.5E-13 | 2.3E-12 |
| 71 | 25 | 3694284 | 3741417 | 4.8E-13 | 4.7E-13 | 3.1E-12 |
| 72 | 25 | 3657454 | 3704589 | 6.6E-13 | 6.4E-13 | 4.0E-12 |
| 73 | 1 | 18109069 | 18227275 | 9.9E-13 | 9.9E-13 | 6.0E-12 |
| 74 | 25 | 3724550 | 3771668 | 1.2E-12 | 1.1E-12 | 6.7E-12 |
| 75 | 25 | 16817685 | 16344818 | 1.3E-12 | 1.4E-12 | 8.2E-12 |
| 76 | 15 | 10100242 | 10371732 | 1.5E-12 | 1.6E-12 | 9.6E-12 |
| 77 | 20 | 27727105 | 28631497 | 2.7E-12 | 2.7E-12 | 1.5E-11 |
| 78 | 20 | 25103556 | 25966714 | 2.3E-12 | 2.8E-12 | 1.7E-11 |
| 79 | 6 | 81651604 | 82815419 | 5.8E-12 | 6.3E-12 | 3.2E-11 |
| 80 | 6 | 81299480 | 82463648 | 9.0E-12 | 9.4E-12 | 4.5E-11 |
| 81 | 20 | 27768899 | 28673289 | 9.9E-12 | 1.1E-11 | 5.1E-11 |
| 82 | 3 | 2506253 | 2638903 | 1.1E-11 | 1.1E-11 | 4.7E-11 |
| 83 | 11 | 36669528 | 36964714 | 1.1E-11 | 1.2E-11 | 5.5E-11 |
| 84 | 23 | 20658789 | 20042543 | 1.2E-11 | 1.3E-11 | 5.7E-11 |
| 85 | 25 | 15829342 | 16084882 | 1.2E-11 | 1.5E-11 | 7.7E-11 |
| 86 | 9 | 75816548 | 77923070 | 1.9E-11 | 2.0E-11 | 8.3E-11 |
| 87 | 6 | 81668230 | 82832046 | 2.0E-11 | 2.1E-11 | 9.2E-11 |
| 88 | 14 | 1388861 | 630035 | 2.2E-11 | 2.1E-11 | 8.6E-11 |
| 89 | 1 | 17548101 | 17666705 | 3.3E-11 | 3.4E-11 | 1.3E-10 |
| 90 | 25 | 3666056 | 3713191 | 4.5E-11 | 4.2E-11 | 1.6E-10 |
| 91 | 25 | 3860478 | 3909047 | 5.6E-11 | 5.5E-11 | 2.0E-10 |
| 92 | 23 | 14814635 | 14181753 | 6.8E-11 | 6.5E-11 | 2.3E-10 |
| 93 | 14 | 1427118 | 668291 | 7.1E-11 | 6.8E-11 | 2.4E-10 |
| 94 | 3 | 2489598 | 2622322 | 8.1E-11 | 7.6E-11 | 2.6E-10 |
| 95 | 1 | 17552161 | 17670765 | 1.1E-10 | 1.2E-10 | 4.0E-10 |
| 96 | 2 | 19698739 | 19746752 | 7.1E-11 | 1.4E-10 | 7.6E-10 |
| 97 | 2 | 19714056 | 19761885 | 8.1E-11 | 1.6E-10 | 9.2E-10 |
| 98 | 16 | 25722825 | 27185509 | 1.7E-10 | 1.7E-10 | 5.7E-10 |
| 99 | 11 | 50634935 | 50978172 | 1.6E-10 | 1.8E-10 | 6.5E-10 |
| 100 | 3 | 58070704 | 59545645 | 1.9E-10 | 1.9E-10 | 6.1E-10 |
| 101 | 25 | 15026761 | 15426560 | 2.0E-10 | 2.3E-10 | 8.1E-10 |
| 102 | 1 | 50226817 | 50590276 | 2.7E-10 | 2.7E-10 | 8.3E-10 |
| 103 | 24 | 10296168 | 10153133 | 4.5E-10 | 5.1E-10 | 1.7E-09 |
| 104 | 20 | 47062579 | 48056952 | 6.6E-10 | 6.7E-10 | 1.9E-09 |
| 105 | 14 | 1570169 | 811504 | 7.0E-10 | 6.9E-10 | 1.9E-09 |
| 106 | 3 | 58077312 | 59552253 | 1.1E-09 | 1.3E-09 | 3.8E-09 |
| 107 | 1 | 35670985 | 35919792 | 1.4E-09 | 1.6E-09 | 4.4E-09 |
| 108 | 25 | 14531198 | 14757120 | 1.6E-09 | 1.7E-09 | 4.6E-09 |
| 109 | 24 | 10299566 | 10156532 | 1.6E-09 | 1.8E-09 | 5.2E-09 |
| 110 | 14 | 1368081 | 609235 | 2.1E-09 | 2.0E-09 | 5.0E-09 |
| 111 | 3 | 53834511 | 55309769 | 2.1E-09 | 2.0E-09 | 5.0E-09 |
| 112 | 20 | 47092658 | 48087031 | 2.8E-09 | 2.8E-09 | 7.0E-09 |
| 113 | 9 | 75803120 | 77909647 | 2.9E-09 | 3.0E-09 | 7.7E-09 |
| 114 | 11 | 36775489 | 37070657 | 3.1E-09 | 3.2E-09 | 7.9E-09 |
| 115 | 29 | 3291497 | 4306133 | 4.4E-09 | 4.9E-09 | 1.2E-08 |
| 116 | 19 | 31393832 | 33925893 | 5.8E-09 | 6.3E-09 | 1.6E-08 |
| 117 | 2 | 19724672 | 19772501 | 3.6E-09 | 6.5E-09 | 2.4E-08 |
| 118 | 19 | 21446218 | 23822276 | 7.4E-09 | 7.7E-09 | 1.8E-08 |
| 119 | 24 | 10285906 | 10142871 | 1.6E-08 | 1.7E-08 | 3.8E-08 |
| 120 | 1 | 39589062 | 39925101 | 2.3E-08 | 2.5E-08 | 5.4E-08 |
| 121 | 11 | 58376457 | 58728978 | 2.3E-08 | 2.5E-08 | 5.5E-08 |
| 122 | 23 | 14813929 | 14181047 | 7.7E-08 | 7.4E-08 | 1.3E-07 |
| 123 | 5 | 66187039 | 63016418 | 9.0E-08 | 1.1E-07 | 2.3E-07 |
| 124 | 17 | 61744016 | 61636653 | 1.7E-07 | 1.7E-07 | 3.1E-07 |
| 125 | 3 | 49601762 | 50982476 | 2.5E-07 | 2.5E-07 | 4.4E-07 |
| 126 | 3 | 49488838 | 50869555 | 4.1E-07 | 4.1E-07 | 6.8E-07 |
| 127 | 2 | 18987527 | 19034724 | 5.4E-07 | 5.2E-07 | 8.2E-07 |
| 128 | 3 | 49857369 | 51237954 | 6.3E-07 | 6.1E-07 | 9.6E-07 |
| 129 | 11 | 29599837 | 29860613 | 6.6E-07 | 6.7E-07 | 1.1E-06 |
| 130 | 3 | 49601886 | 50982600 | 6.6E-07 | 6.8E-07 | 1.1E-06 |
| 131 | 3 | 49530033 | 50910750 | 6.8E-07 | 7.2E-07 | 1.2E-06 |
| 132 | 25 | 12791659 | 12808675 | 7.6E-07 | 7.4E-07 | 1.2E-06 |
| 133 | 2 | 18364832 | 18410480 | 8.3E-07 | 8.9E-07 | 1.5E-06 |
| 134 | 17 | 61728019 | 61620654 | 8.9E-07 | 9.3E-07 | 1.5E-06 |
| 135 | 11 | 29564206 | 29824000 | 9.4E-07 | 9.6E-07 | 1.5E-06 |
| 136 | 25 | 14737344 | 15074102 | 1.1E-06 | 1.1E-06 | 1.7E-06 |
| 137 | 3 | 49857478 | 51238063 | 1.1E-06 | 1.1E-06 | 1.8E-06 |
| 138 | 2 | 19775173 | 19822898 | 1.2E-06 | 1.1E-06 | 1.7E-06 |
| 139 | 17 | 61749334 | 61641971 | 1.3E-06 | 1.3E-06 | 2.0E-06 |
| 140 | 11 | 29532466 | 29792244 | 1.6E-06 | 1.6E-06 | 2.5E-06 |
| 141 | 25 | 12758770 | 12771053 | 1.7E-06 | 1.7E-06 | 2.5E-06 |
| 142 | 17 | 61717590 | 61610225 | 2.0E-06 | 2.1E-06 | 3.2E-06 |
| 143 | 3 | 56755586 | 58228638 | 2.1E-06 | 2.1E-06 | 3.1E-06 |
| 144 | 3 | 52318025 | 53791546 | 2.0E-06 | 2.2E-06 | 3.5E-06 |
| 145 | 3 | 57929520 | 59404415 | 2.3E-06 | 2.3E-06 | 3.3E-06 |
| 146 | 23 | 14182456 | 13549629 | 2.6E-06 | 2.5E-06 | 3.6E-06 |
| 147 | 3 | 58434545 | 59909227 | 2.5E-06 | 2.5E-06 | 3.7E-06 |
| 148 | 25 | 14760167 | 15076230 | 3.6E-06 | 3.7E-06 | 5.4E-06 |
| 149 | 25 | 14735220 | 14870137 | 5.2E-06 | 5.3E-06 | 7.6E-06 |
| 150 | 3 | 52680823 | 54154434 | 6.7E-06 | 6.7E-06 | 9.4E-06 |
| 151 | 12 | 16262259 | 19692637 | 7.9E-06 | 7.7E-06 | 1.0E-05 |
| 152 | 1 | 43245806 | 43587228 | 9.6E-06 | 9.3E-06 | 1.2E-05 |
| 153 | 25 | 13050176 | 13099856 | 1.1E-05 | 1.1E-05 | 1.5E-05 |
| 154 | 25 | 13047191 | 13096870 | 1.2E-05 | 1.2E-05 | 1.6E-05 |
| 155 | 2 | 18538576 | 18583657 | 1.3E-05 | 1.2E-05 | 1.6E-05 |
| 156 | 25 | 13021802 | 13071456 | 1.2E-05 | 1.2E-05 | 1.6E-05 |
| 157 | 25 | 13052616 | 13102296 | 1.4E-05 | 1.3E-05 | 1.8E-05 |
| 158 | 17 | 28485796 | 28388023 | 1.4E-05 | 1.3E-05 | 1.8E-05 |
| 159 | 3 | 56561263 | 58034316 | 1.4E-05 | 1.4E-05 | 1.9E-05 |
| 160 | 1 | 50226814 | 50590273 | 1.7E-05 | 1.6E-05 | 2.1E-05 |
| 161 | 25 | 13031250 | 13080925 | 1.6E-05 | 1.6E-05 | 2.1E-05 |
| 162 | 12 | 16270318 | 19700697 | 1.7E-05 | 1.7E-05 | 2.2E-05 |
| 163 | 26 | 3315939 | 3711296 | 1.8E-05 | 1.7E-05 | 2.2E-05 |
| 164 | 24 | 10276151 | 10133116 | 1.7E-05 | 1.8E-05 | 2.5E-05 |
| 165 | 5 | 66199885 | 63029263 | 2.5E-05 | 2.7E-05 | 3.8E-05 |
| 166 | 1 | 5322242 | 5348911 | 3.7E-05 | 3.7E-05 | 4.6E-05 |
| 167 | 3 | 57629621 | 59104513 | 3.9E-05 | 3.9E-05 | 4.8E-05 |
| 168 | 26 | 3315959 | 3711316 | 4.1E-05 | 4.1E-05 | 5.1E-05 |
| 169 | 26 | 3315992 | 3711349 | 6.7E-05 | 6.6E-05 | 8.0E-05 |
| 170 | 25 | 13016645 | 13066298 | 7.3E-05 | 7.2E-05 | 8.8E-05 |
| 171 | 26 | 3315794 | 3711151 | 7.7E-05 | 7.6E-05 | 9.2E-05 |
| 172 | 11 | 31319004 | 31617654 | 1.0E-04 | 1.0E-04 | 1.2E-04 |
| 173 | 11 | 31303355 | 31602010 | 1.2E-04 | 1.2E-04 | 1.5E-04 |
| 174 | 9 | 75896366 | 78003195 | 1.3E-04 | 1.4E-04 | 1.7E-04 |
| 175 | 25 | 15621763 | 16001917 | 1.6E-04 | 1.5E-04 | 1.8E-04 |
| 176 | 6 | 6609510 | 6384625 | 1.9E-04 | 1.8E-04 | 2.1E-04 |
| 177 | 3 | 49785110 | 51165739 | 1.9E-04 | 1.9E-04 | 2.2E-04 |
