# Supplementary material for: Identification and validation of genetic variants predictive of gait in standardbred horses
Source: PLoS Genet. 2019 May 28;15(5):e1008146. doi: 10.1371/journal.pgen.1008146 (PMC6555539; doi:10.1371/journal.pgen.1008146)

**Supplemental Figure 1:** Footfall patterns for the trot (2-beat, symmetrical, diagonal), pace (2-beat, symmetrical, lateral), and tölt (4-beat, symmetrical lateral). The tölt is shown in 8 phases to incorporate the half-suspension phases of the forelimbs and hind limbs seen at this gait. The hind limb half-suspension phase is eliminated at slow speeds, resulting in weightbearing on three limbs instead of one. Hoofprints that are filled in black are supporting limbs (on the ground), those with light grey fill and a dashed outline are suspended (in the air).


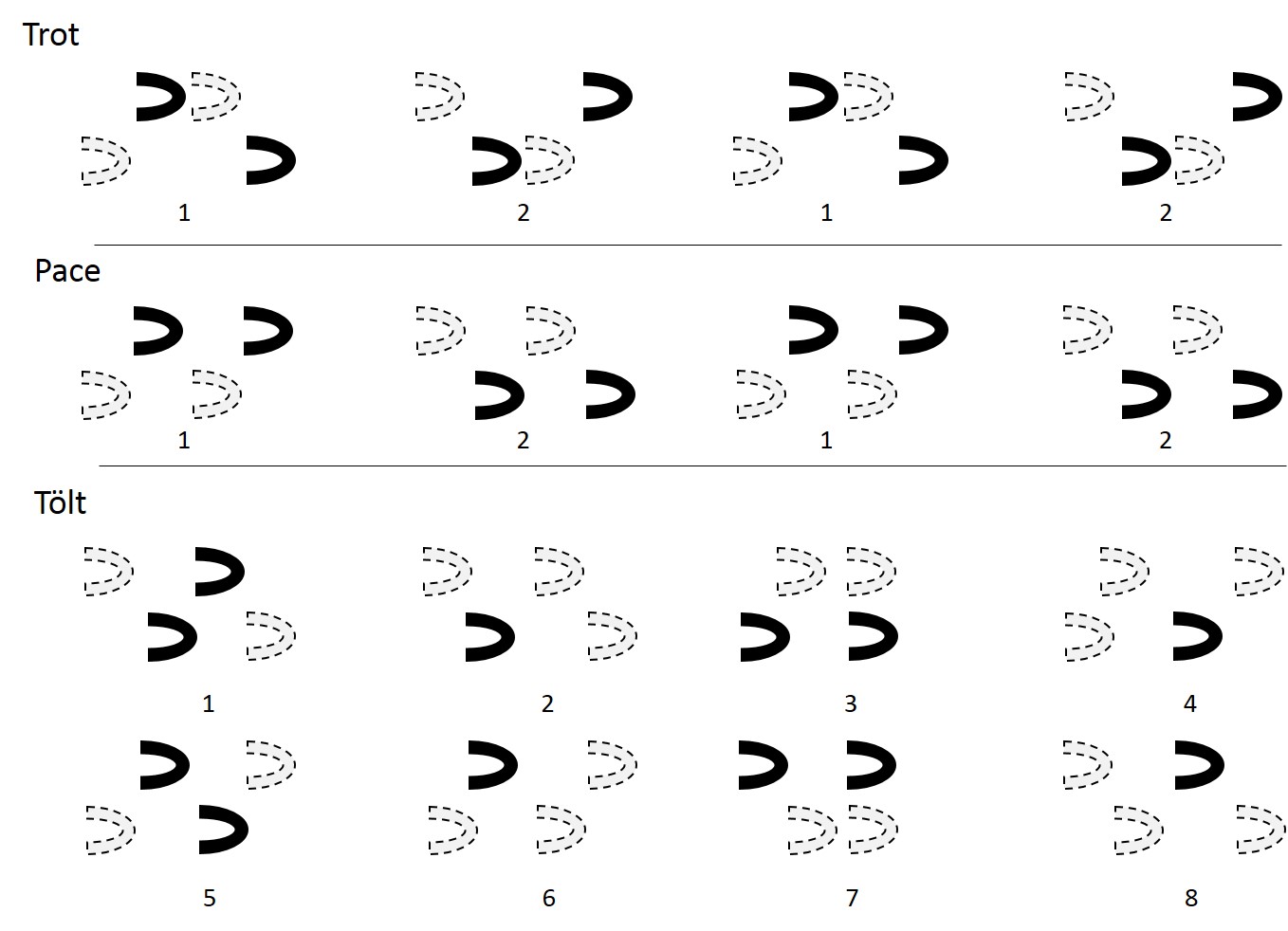

Supplement: S1 Fig — (DOCX) [file pgen.1008146.s006.docx]
