# Supplementary material for: Identification and validation of genetic variants predictive of gait in standardbred horses
Source: PLoS Genet. 2019 May 28;15(5):e1008146. doi: 10.1371/journal.pgen.1008146 (PMC6555539; doi:10.1371/journal.pgen.1008146)
Supplement: S2 Fig — (DOCX) [file pgen.1008146.s007.docx]

**Supplemental Figure 2:** Multi-dimensional scaling (MDS) plot of 542 Standardbred horses (366 trotters, 176 pacers) based on genome-wide genotyping data. The two groups are genetically distinct, with minimal admixture.


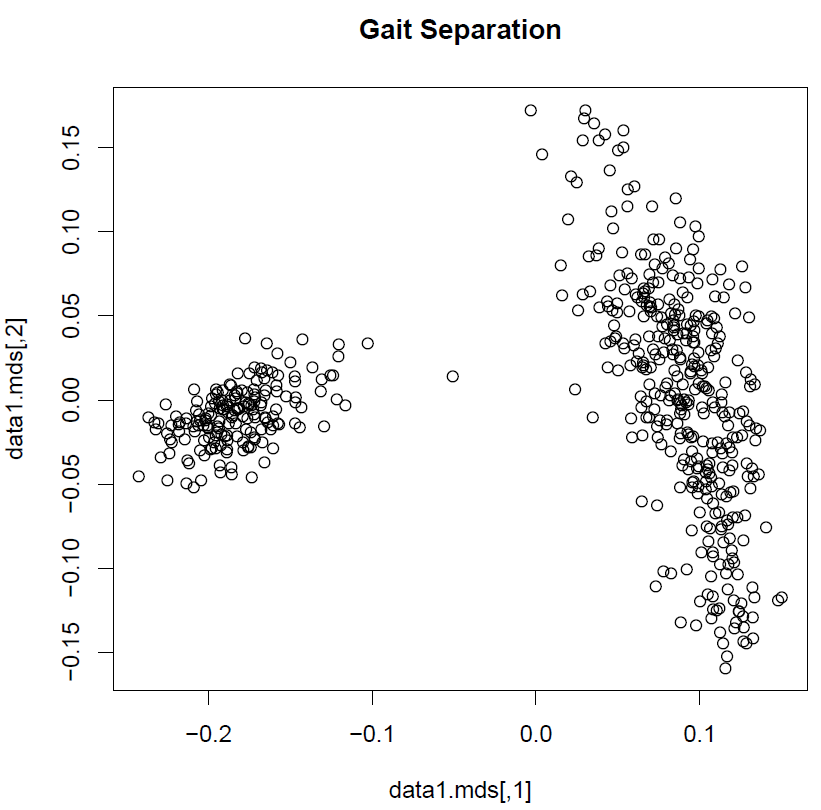


Pacers

Trotters
